# Supplementary material for: Development of an optimized method for processing peripheral blood mononuclear cells for 1H-nuclear magnetic resonance-based metabolomic profiling
Source: PLoS One. 2021 Feb 25;16(2):e0247668. doi: 10.1371/journal.pone.0247668 (PMC7906414; doi:10.1371/journal.pone.0247668)
Supplement: S1 Table — (PDF) [file pone.0247668.s001.pdf]

	Method	Patient	Replicated	Date							
**Spectrum 1**	UUM	1	1	4/09/20	0,0428	-0,0466	0,669768	38742,6	1,058	1,0337	0,0228039
**Spectrum 2**	UUM	2	1	4/09/20	0,0428	-0,0466	0,379276	39355	1,058	1,0337	0,00870788
**Spectrum 3**	UUM	3	1	4/09/20	0,0428	-0,0466	0,466371	37019,1	1,058	1,0337	0,0141657
**Spectrum 4**	UUM	1	2	5/09/20	0,0428	-0,0466	0,805273	40322,2	1,058	1,0337	0,0266864
**Spectrum 5**	UUM	2	2	5/09/20	0,0428	-0,0466	0,880212	40746,5	1,058	1,0337	0,0257667
**Spectrum 6**	UUM	3	2	5/09/20	0,0428	-0,0466	0,718798	40137,1	1,058	1,0337	0,0183641
**Spectrum 7**	UM	1	1	4/09/20	0,0428	-0,0466	1,08906	11,4786	1,058	1,0337	0,106961
**Spectrum 8**	UM	2	1	4/09/20	0,0428	-0,0466	1,05051	10,7792	1,058	1,0337	0,100102
**Spectrum 9**	UM	3	1	4/09/20	0,0428	-0,0466	1,02399	11,7008	1,058	1,0337	0,0805803
**Spectrum 10**	UM	1	2	5/09/20	0,0428	-0,0466	1,04959	10,8352	1,058	1,0337	0,100218
**Spectrum 11**	UM	2	2	5/09/20	0,0428	-0,0466	1,0059	12,7506	1,058	1,0337	0,0755193
**Spectrum 12**	UM	3	2	5/09/20	0,0428	-0,0466	1,02089	12,7713	1,058	1,0337	0,0601884
**Spectrum 13**	FM	1	2	17/09/20	0,0428	-0,0466	1,02528	38846,4	1,058	1,0337	0,00647437
**Spectrum 14**	FM	1	1	17/09/20	0,0428	-0,0466	1,16681	38952,6	1,058	1,0337	0,0130396
**Spectrum 15**	FM	2	1	17/09/20	0,0428	-0,0466	1,0858	39037,1	1,058	1,0337	0,00787668
**Spectrum 16**	FM	2	2	17/09/20	0,0428	-0,0466	1,05606	39333,2	1,058	1,0337	0,00738306
**Spectrum 17**	FM	3	1	17/09/20	0,0428	-0,0466	1,29517	39081,1	1,058	1,0337	0,00787262
**Spectrum 18**	FM	3	2	17/09/20	0,0428	-0,0466	1,27465	38755,7	1,058	1,0337	0,00899654
**Spectrum 19**	UM	0	3	20/12/19	0,0428	-0,0466	1,0879	31146,3	1,058	1,0337	0,138726
**Spectrum 20**	UM	0	2	20/12/19	0,0428	-0,0466	0,950872	32374,1	1,058	1,0337	0,184812
**Spectrum 21**	UM	0	1	20/12/19	0,0428	-0,0466	1,01386	34852,7	1,058	1,0337	0,132121
**Spectrum 22**	UUM	0	3	27/12/19	0,0428	-0,0466	0,122182	42318,4	1,058	1,0337	0,0129333
**Spectrum 23**	UUM	0	2	27/12/19	0,0428	-0,0466	0,152941	43615,7	1,058	1,0337	0,0125114
**Spectrum 24**	UUM	0	1	27/12/19	0,0428	-0,0466	0,127508	43660,5	1,058	1,0337	0,00975333
**Spectrum 25**	FM	0	3	4/02/20	0,0428	-0,0466	1,11093	45224,4	1,058	1,0337	0,0080433
**Spectrum 26**	FM	0	2	28/12/19	0,0428	-0,0466	1,0951	52833,4	1,058	1,0337	0,0183904
**Spectrum 27**	FM	0	1	28/12/19	0,0428	-0,0466	1,1282	51617,6	1,058	1,0337	0,0311048

	Method	Patient	Replicated	Date							
**Spectrum 1**	UUM	1	1	4/09/20	1319,08	1,0248	1,0016	0,0140099	810,396	1,0016	0,9799
**Spectrum 2**	UUM	2	1	4/09/20	903,559	1,0248	1,0016	0,00519436	538,984	1,0016	0,9799
**Spectrum 3**	UUM	3	1	4/09/20	1124,43	1,0248	1,0016	0,0085303	677,108	1,0016	0,9799
**Spectrum 4**	UUM	1	2	5/09/20	1336,26	1,0248	1,0016	0,0164859	825,496	1,0016	0,9799
**Spectrum 5**	UUM	2	2	5/09/20	1192,79	1,0248	1,0016	0,0157529	729,229	1,0016	0,9799
**Spectrum 6**	UUM	3	2	5/09/20	1025,44	1,0248	1,0016	0,012677	707,875	1,0016	0,9799
**Spectrum 7**	UM	1	1	4/09/20	1,12737	1,0248	1,0016	0,0799664	0,842839	1,0016	0,9799
**Spectrum 8**	UM	2	1	4/09/20	1,02714	1,0248	1,0016	0,0807449	0,828517	1,0016	0,9799
**Spectrum 9**	UM	3	1	4/09/20	0,920765	1,0248	1,0016	0,0684976	0,782701	1,0016	0,9799
**Spectrum 10**	UM	1	2	5/09/20	1,03457	1,0248	1,0016	0,0825447	0,852127	1,0016	0,9799
**Spectrum 11**	UM	2	2	5/09/20	0,957264	1,0248	1,0016	0,0643095	0,815171	1,0016	0,9799
**Spectrum 12**	UM	3	2	5/09/20	0,752953	1,0248	1,0016	0,064611	0,808279	1,0016	0,9799
**Spectrum 13**	FM	1	2	17/09/20	245,305	1,0248	1,0016	0,00454604	172,244	1,0016	0,9799
**Spectrum 14**	FM	1	1	17/09/20	435,311	1,0248	1,0016	0,0103604	345,87	1,0016	0,9799
**Spectrum 15**	FM	2	1	17/09/20	283,187	1,0248	1,0016	0,00734875	264,206	1,0016	0,9799
**Spectrum 16**	FM	2	2	17/09/20	274,983	1,0248	1,0016	0,00604871	225,285	1,0016	0,9799
**Spectrum 17**	FM	3	1	17/09/20	237,552	1,0248	1,0016	0,00392718	118,5	1,0016	0,9799
**Spectrum 18**	FM	3	2	17/09/20	273,54	1,0248	1,0016	0,0064914	197,371	1,0016	0,9799
**Spectrum 19**	UM	0	3	20/12/19	3971,67	1,0248	1,0016	0,0884958	2533,61	1,0016	0,9799
**Spectrum 20**	UM	0	2	20/12/19	6292,24	1,0248	1,0016	0,116608	3970,1	1,0016	0,9799
**Spectrum 21**	UM	0	1	20/12/19	4541,83	1,0248	1,0016	0,0866485	2978,66	1,0016	0,9799
**Spectrum 22**	UUM	0	3	27/12/19	4479,49	1,0248	1,0016	0,00878317	3042,09	1,0016	0,9799
**Spectrum 23**	UUM	0	2	27/12/19	3568	1,0248	1,0016	0,00785003	2238,68	1,0016	0,9799
**Spectrum 24**	UUM	0	1	27/12/19	3339,68	1,0248	1,0016	0,00600423	2055,93	1,0016	0,9799
**Spectrum 25**	FM	0	3	4/02/20	327,431	1,0248	1,0016	0,00440986	179,519	1,0016	0,9799
**Spectrum 26**	FM	0	2	28/12/19	887,253	1,0248	1,0016	0,0142497	687,483	1,0016	0,9799
**Spectrum 27**	FM	0	1	28/12/19	1423,11	1,0248	1,0016	0,029305	1340,76	1,0016	0,9799

	Method	Patient	Replicated	Date							
**Spectrum 1**	UUM	1	1	4/09/20	0,0243451	1408,24	0,9799	0,9503	0,0680934	3938,84	0,9354
**Spectrum 2**	UUM	2	1	4/09/20	0,00892887	926,489	0,9799	0,9503	0,0277436	2878,78	0,9354
**Spectrum 3**	UUM	3	1	4/09/20	0,016124	1279,87	0,9799	0,9503	0,044283	3515,05	0,9354
**Spectrum 4**	UUM	1	2	5/09/20	0,0314997	1577,28	0,9799	0,9503	0,0861676	4314,65	0,9354
**Spectrum 5**	UUM	2	2	5/09/20	0,0296213	1371,22	0,9799	0,9503	0,0855969	3962,43	0,9354
**Spectrum 6**	UUM	3	2	5/09/20	0,019916	1112,09	0,9799	0,9503	0,0552877	3087,22	0,9354
**Spectrum 7**	UM	1	1	4/09/20	0,141116	1,48735	0,9799	0,9503	0,385022	4,0581	0,9354
**Spectrum 8**	UM	2	1	4/09/20	0,135976	1,39524	0,9799	0,9503	0,384729	3,94767	0,9354
**Spectrum 9**	UM	3	1	4/09/20	0,105462	1,20509	0,9799	0,9503	0,283521	3,2397	0,9354
**Spectrum 10**	UM	1	2	5/09/20	0,137053	1,41483	0,9799	0,9503	0,378015	3,90233	0,9354
**Spectrum 11**	UM	2	2	5/09/20	0,105712	1,33997	0,9799	0,9503	0,297976	3,77707	0,9354
**Spectrum 12**	UM	3	2	5/09/20	0,0981241	1,22753	0,9799	0,9503	0,28976	3,62488	0,9354
**Spectrum 13**	FM	1	2	17/09/20	0,0100187	379,595	0,9799	0,9503	0,0280061	1061,11	0,9354
**Spectrum 14**	FM	1	1	17/09/20	0,0144598	482,721	0,9799	0,9503	0,041619	1389,4	0,9354
**Spectrum 15**	FM	2	1	17/09/20	0,0126935	456,364	0,9799	0,9503	0,0293669	1055,81	0,9354
**Spectrum 16**	FM	2	2	17/09/20	0,00845404	314,872	0,9799	0,9503	0,0227765	848,313	0,9354
**Spectrum 17**	FM	3	1	17/09/20	0,0106995	322,852	0,9799	0,9503	0,0271335	818,739	0,9354
**Spectrum 18**	FM	3	2	17/09/20	0,0108785	330,762	0,9799	0,9503	0,0251242	763,903	0,9354
**Spectrum 19**	UM	0	3	20/12/19	0,17785	5091,8	0,9799	0,9503	0,458194	13118	0,9354
**Spectrum 20**	UM	0	2	20/12/19	0,225386	7673,66	0,9799	0,9503	0,556585	18949,9	0,9354
**Spectrum 21**	UM	0	1	20/12/19	0,168567	5794,72	0,9799	0,9503	0,426251	14653	0,9354
**Spectrum 22**	UUM	0	3	27/12/19	0,0119631	4143,48	0,9799	0,9503	0,0240102	8316,03	0,9354
**Spectrum 23**	UUM	0	2	27/12/19	0,0113871	3247,37	0,9799	0,9503	0,0263688	7519,88	0,9354
**Spectrum 24**	UUM	0	1	27/12/19	0,00957939	3280,12	0,9799	0,9503	0,0229167	7846,98	0,9354
**Spectrum 25**	FM	0	3	4/02/20	0,0125993	512,898	0,9799	0,9503	0,0361607	1472,05	0,9354
**Spectrum 26**	FM	0	2	28/12/19	0,027907	1346,38	0,9799	0,9503	0,0838998	4047,77	0,9354
**Spectrum 27**	FM	0	1	28/12/19	0,0555255	2540,4	0,9799	0,9503	0,150573	6889,01	0,9354

	Method	Patient	Replicated	Date							
**Spectrum 1**	UUM	1	1	4/09/20	0,9247	0,00563459	325,931	1,6294	1,5908	0,0554302	3206,35
**Spectrum 2**	UUM	2	1	4/09/20	0,9247	0,00187115	194,157	1,6294	1,5908	0,0311591	3233,17
**Spectrum 3**	UUM	3	1	4/09/20	0,9247	0,00285729	226,803	1,6294	1,5908	0,0313729	2490,28
**Spectrum 4**	UUM	1	2	5/09/20	0,9247	0,00471158	235,922	1,6294	1,5908	0,0666856	3339,13
**Spectrum 5**	UUM	2	2	5/09/20	0,9247	0,00597303	276,502	1,6294	1,5908	0,0733809	3396,93
**Spectrum 6**	UUM	3	2	5/09/20	0,9247	0,00447683	249,983	1,6294	1,5908	0,06216	3470,96
**Spectrum 7**	UM	1	1	4/09/20	0,9247	0,123165	1,29815	1,6294	1,5908	0,142518	1,50213
**Spectrum 8**	UM	2	1	4/09/20	0,9247	0,124904	1,28163	1,6294	1,5908	0,142172	1,45882
**Spectrum 9**	UM	3	1	4/09/20	0,9247	0,0989555	1,13073	1,6294	1,5908	0,118163	1,35021
**Spectrum 10**	UM	1	2	5/09/20	0,9247	0,130818	1,35046	1,6294	1,5908	0,152525	1,57455
**Spectrum 11**	UM	2	2	5/09/20	0,9247	0,0983019	1,24605	1,6294	1,5908	0,116748	1,47986
**Spectrum 12**	UM	3	2	5/09/20	0,9247	0,110532	1,38275	1,6294	1,5908	0,122556	1,53316
**Spectrum 13**	FM	1	2	17/09/20	0,9247	0,0107219	406,24	1,6294	1,5908	0,0120129	455,152
**Spectrum 14**	FM	1	1	17/09/20	0,9247	0,0161988	540,778	1,6294	1,5908	0,0200958	670,873
**Spectrum 15**	FM	2	1	17/09/20	0,9247	0,0124087	446,124	1,6294	1,5908	0,0141013	506,978
**Spectrum 16**	FM	2	2	17/09/20	0,9247	0,00965383	359,558	1,6294	1,5908	0,011285	420,311
**Spectrum 17**	FM	3	1	17/09/20	0,9247	0,0101596	306,561	1,6294	1,5908	0,00830414	250,573
**Spectrum 18**	FM	3	2	17/09/20	0,9247	0,00923988	280,939	1,6294	1,5908	0,0100508	305,596
**Spectrum 19**	UM	0	3	20/12/19	0,9247	0,123024	3522,15	1,6294	1,5908	0,1083	3100,6
**Spectrum 20**	UM	0	2	20/12/19	0,9247	0,126686	4313,24	1,6294	1,5908	0,110327	3756,29
**Spectrum 21**	UM	0	1	20/12/19	0,9247	0,106421	3658,38	1,6294	1,5908	0,0964874	3316,89
**Spectrum 22**	UUM	0	3	27/12/19	0,9247	0,00231047	800,241	1,6294	1,5908	0,00670934	2323,81
**Spectrum 23**	UUM	0	2	27/12/19	0,9247	0,00219452	625,835	1,6294	1,5908	0,00508462	1450,04
**Spectrum 24**	UUM	0	1	27/12/19	0,9247	0,0018341	628,021	1,6294	1,5908	0,00689573	2361,2
**Spectrum 25**	FM	0	3	4/02/20	0,9247	0,011741	477,959	1,6294	1,5908	0,0122	496,642
**Spectrum 26**	FM	0	2	28/12/19	0,9247	0,0223124	1076,47	1,6294	1,5908	0,0242828	1171,53
**Spectrum 27**	FM	0	1	28/12/19	0,9247	0,0423459	1937,41	1,6294	1,5908	0,0499395	2284,83

	Method	Patient	Replicated	Date							
**Spectrum 1**	UUM	1	1	4/09/20	1,6653	1,6299	0,0587726	3399,68	0,9479	0,9359	0,0113277
**Spectrum 2**	UUM	2	1	4/09/20	1,6653	1,6299	0,032068	3327,49	0,9479	0,9359	0,00420763
**Spectrum 3**	UUM	3	1	4/09/20	1,6653	1,6299	0,0338182	2684,38	0,9479	0,9359	0,00543167
**Spectrum 4**	UUM	1	2	5/09/20	1,6653	1,6299	0,0736925	3689,99	0,9479	0,9359	0,01158
**Spectrum 5**	UUM	2	2	5/09/20	1,6653	1,6299	0,0767117	3551,11	0,9479	0,9359	0,011826
**Spectrum 6**	UUM	3	2	5/09/20	1,6653	1,6299	0,061291	3422,44	0,9479	0,9359	0,00837579
**Spectrum 7**	UM	1	1	4/09/20	1,6653	1,6299	0,173422	1,82786	0,9479	0,9359	0,156862
**Spectrum 8**	UM	2	1	4/09/20	1,6653	1,6299	0,175876	1,80465	0,9479	0,9359	0,157796
**Spectrum 9**	UM	3	1	4/09/20	1,6653	1,6299	0,143111	1,63528	0,9479	0,9359	0,121606
**Spectrum 10**	UM	1	2	5/09/20	1,6653	1,6299	0,186188	1,92206	0,9479	0,9359	0,164306
**Spectrum 11**	UM	2	2	5/09/20	1,6653	1,6299	0,143816	1,82298	0,9479	0,9359	0,125173
**Spectrum 12**	UM	3	2	5/09/20	1,6653	1,6299	0,152633	1,90942	0,9479	0,9359	0,140483
**Spectrum 13**	FM	1	2	17/09/20	1,6653	1,6299	0,0159076	602,717	0,9479	0,9359	0,015096
**Spectrum 14**	FM	1	1	17/09/20	1,6653	1,6299	0,0270371	902,6	0,9479	0,9359	0,0216067
**Spectrum 15**	FM	2	1	17/09/20	1,6653	1,6299	0,0178141	640,463	0,9479	0,9359	0,0158187
**Spectrum 16**	FM	2	2	17/09/20	1,6653	1,6299	0,0154869	576,812	0,9479	0,9359	0,0112642
**Spectrum 17**	FM	3	1	17/09/20	1,6653	1,6299	0,0140382	423,594	0,9479	0,9359	0,0129642
**Spectrum 18**	FM	3	2	17/09/20	1,6653	1,6299	0,0126794	385,518	0,9479	0,9359	0,0120031
**Spectrum 19**	UM	0	3	20/12/19	1,6653	1,6299	0,158433	4535,88	0,9479	0,9359	0,165004
**Spectrum 20**	UM	0	2	20/12/19	1,6653	1,6299	0,170465	5803,79	0,9479	0,9359	0,173154
**Spectrum 21**	UM	0	1	20/12/19	1,6653	1,6299	0,141254	4855,81	0,9479	0,9359	0,144175
**Spectrum 22**	UUM	0	3	27/12/19	1,6653	1,6299	0,00814131	2819,78	0,9479	0,9359	0,00385382
**Spectrum 23**	UUM	0	2	27/12/19	1,6653	1,6299	0,00708837	2021,47	0,9479	0,9359	0,00397782
**Spectrum 24**	UUM	0	1	27/12/19	1,6653	1,6299	0,00827032	2831,87	0,9479	0,9359	0,00309133
**Spectrum 25**	FM	0	3	4/02/20	1,6653	1,6299	0,0148387	604,063	0,9479	0,9359	0,0172509
**Spectrum 26**	FM	0	2	28/12/19	1,6653	1,6299	0,0295475	1425,53	0,9479	0,9359	0,0319779
**Spectrum 27**	FM	0	1	28/12/19	1,6653	1,6299	0,0570424	2609,81	0,9479	0,9359	0,0594106

	Method	Patient	Replicated	Date							
**Spectrum 1**	UUM	1	1	4/09/20	655,246	1,2478	1,1354	1,78423	103208	3,6236	3,6086
**Spectrum 2**	UUM	2	1	4/09/20	436,598	1,2478	1,1354	1,0334	107229	3,6236	3,6086
**Spectrum 3**	UUM	3	1	4/09/20	431,149	1,2478	1,1354	1,77109	140583	3,6236	3,6086
**Spectrum 4**	UUM	1	2	5/09/20	579,843	1,2478	1,1354	1,85615	92942,7	3,6236	3,6086
**Spectrum 5**	UUM	2	2	5/09/20	547,444	1,2478	1,1354	1,93275	89470,2	3,6236	3,6086
**Spectrum 6**	UUM	3	2	5/09/20	467,697	1,2478	1,1354	1,23803	69130,8	3,6236	3,6086
**Spectrum 7**	UM	1	1	4/09/20	1,65332	1,2478	1,1354	0,468703	4,94009	3,6236	3,6086
**Spectrum 8**	UM	2	1	4/09/20	1,61913	1,2478	1,1354	0,435472	4,46834	3,6236	3,6086
**Spectrum 9**	UM	3	1	4/09/20	1,38955	1,2478	1,1354	0,393531	4,49675	3,6236	3,6086
**Spectrum 10**	UM	1	2	5/09/20	1,69617	1,2478	1,1354	0,41577	4,29209	3,6236	3,6086
**Spectrum 11**	UM	2	2	5/09/20	1,58666	1,2478	1,1354	0,368364	4,66929	3,6236	3,6086
**Spectrum 12**	UM	3	2	5/09/20	1,75743	1,2478	1,1354	0,35975	4,50044	3,6236	3,6086
**Spectrum 13**	FM	1	2	17/09/20	571,966	1,2478	1,1354	0,0552063	2091,7	3,6236	3,6086
**Spectrum 14**	FM	1	1	17/09/20	721,315	1,2478	1,1354	0,116603	3892,65	3,6236	3,6086
**Spectrum 15**	FM	2	1	17/09/20	568,724	1,2478	1,1354	0,109221	3926,77	3,6236	3,6086
**Spectrum 16**	FM	2	2	17/09/20	419,538	1,2478	1,1354	0,0576901	2148,68	3,6236	3,6086
**Spectrum 17**	FM	3	1	17/09/20	391,186	1,2478	1,1354	0,056366	1700,81	3,6236	3,6086
**Spectrum 18**	FM	3	2	17/09/20	364,955	1,2478	1,1354	0,0793943	2413,99	3,6236	3,6086
**Spectrum 19**	UM	0	3	20/12/19	4724	1,2478	1,1354	0,355358	10173,8	3,6236	3,6086
**Spectrum 20**	UM	0	2	20/12/19	5895,32	1,2478	1,1354	0,337734	11498,7	3,6236	3,6086
**Spectrum 21**	UM	0	1	20/12/19	4956,22	1,2478	1,1354	0,293035	10073,5	3,6236	3,6086
**Spectrum 22**	UUM	0	3	27/12/19	1334,79	1,2478	1,1354	7,08992	2,46E+06	3,6236	3,6086
**Spectrum 23**	UUM	0	2	27/12/19	1134,4	1,2478	1,1354	7,0339	2,01E+06	3,6236	3,6086
**Spectrum 24**	UUM	0	1	27/12/19	1058,51	1,2478	1,1354	6,30658	2,16E+06	3,6236	3,6086
**Spectrum 25**	FM	0	3	4/02/20	702,258	1,2478	1,1354	0,152946	6226,21	3,6236	3,6086
**Spectrum 26**	FM	0	2	28/12/19	1542,78	1,2478	1,1354	0,126102	6083,81	3,6236	3,6086
**Spectrum 27**	FM	0	1	28/12/19	2718,16	1,2478	1,1354	0,175871	8046,45	3,6236	3,6086

	Method	Patient	Replicated	Date							
**Spectrum 1**	UUM	1	1	4/09/20	0,0218375	1263,19	1,2576	1,2508	0,00322738	186,687	1,3503
**Spectrum 2**	UUM	2	1	4/09/20	0,0111159	1153,42	1,2576	1,2508	0,00158461	164,424	1,3503
**Spectrum 3**	UUM	3	1	4/09/20	0,0170172	1350,77	1,2576	1,2508	0,00260031	206,404	1,3503
**Spectrum 4**	UUM	1	2	5/09/20	0,0218463	1093,91	1,2576	1,2508	0,00472472	236,58	1,3503
**Spectrum 5**	UUM	2	2	5/09/20	0,0196647	910,31	1,2576	1,2508	0,00296711	137,353	1,3503
**Spectrum 6**	UUM	3	2	5/09/20	0,0172963	965,812	1,2576	1,2508	0,00241407	134,8	1,3503
**Spectrum 7**	UM	1	1	4/09/20	0,0408265	0,430308	1,2576	1,2508	0,0197988	0,208677	1,3503
**Spectrum 8**	UM	2	1	4/09/20	0,046202	0,474075	1,2576	1,2508	0,0196209	0,201328	1,3503
**Spectrum 9**	UM	3	1	4/09/20	0,0374517	0,427949	1,2576	1,2508	0,020784	0,237492	1,3503
**Spectrum 10**	UM	1	2	5/09/20	0,0376353	0,388517	1,2576	1,2508	0,021539	0,222351	1,3503
**Spectrum 11**	UM	2	2	5/09/20	0,0348903	0,442261	1,2576	1,2508	0,0171203	0,217012	1,3503
**Spectrum 12**	UM	3	2	5/09/20	0,0122681	0,153472	1,2576	1,2508	0,0168256	0,210486	1,3503
**Spectrum 13**	FM	1	2	17/09/20	0,00706016	267,5	1,2576	1,2508	0,00634038	240,229	1,3503
**Spectrum 14**	FM	1	1	17/09/20	0,0062133	207,424	1,2576	1,2508	0,0125122	417,706	1,3503
**Spectrum 15**	FM	2	1	17/09/20	0,00332699	119,614	1,2576	1,2508	0,0102215	367,49	1,3503
**Spectrum 16**	FM	2	2	17/09/20	0,00798851	297,533	1,2576	1,2508	0,00586259	218,353	1,3503
**Spectrum 17**	FM	3	1	17/09/20	0,00297947	89,9038	1,2576	1,2508	0,00610968	184,356	1,3503
**Spectrum 18**	FM	3	2	17/09/20	0,00577117	175,473	1,2576	1,2508	0,00714879	217,359	1,3503
**Spectrum 19**	UM	0	3	20/12/19	0,0205878	589,423	1,2576	1,2508	0,0161615	462,698	1,3503
**Spectrum 20**	UM	0	2	20/12/19	0,028526	971,215	1,2576	1,2508	0,0165535	563,594	1,3503
**Spectrum 21**	UM	0	1	20/12/19	0,019757	679,172	1,2576	1,2508	0,0131733	452,852	1,3503
**Spectrum 22**	UUM	0	3	27/12/19	0,00196553	680,772	1,2576	1,2508	0,0134751	4667,16	1,3503
**Spectrum 23**	UUM	0	2	27/12/19	0,00048895	139,438	1,2576	1,2508	0,00315477	899,679	1,3503
**Spectrum 24**	UUM	0	1	27/12/19	0,00130974	448,473	1,2576	1,2508	0,00340986	1167,58	1,3503
**Spectrum 25**	FM	0	3	4/02/20	0,00934001	380,218	1,2576	1,2508	0,00599168	243,912	1,3503
**Spectrum 26**	FM	0	2	28/12/19	0,00925307	446,417	1,2576	1,2508	0,00646616	311,962	1,3503
**Spectrum 27**	FM	0	1	28/12/19	0,0207488	949,299	1,2576	1,2508	0,00952701	435,88	1,3503

	Method	Patient	Replicated	Date							
**Spectrum 1**	UUM	1	1	4/09/20	1,3136	0,187872	10867,4	1,5001	1,4645	0,0921387	5329,73
**Spectrum 2**	UUM	2	1	4/09/20	1,3136	0,0596435	6188,81	1,5001	1,4645	0,0291899	3028,84
**Spectrum 3**	UUM	3	1	4/09/20	1,3136	0,084584	6714,01	1,5001	1,4645	0,0518776	4117,88
**Spectrum 4**	UUM	1	2	5/09/20	1,3136	0,168106	8417,51	1,5001	1,4645	0,116215	5819,21
**Spectrum 5**	UUM	2	2	5/09/20	1,3136	0,0914476	4233,27	1,5001	1,4645	0,089487	4142,5
**Spectrum 6**	UUM	3	2	5/09/20	1,3136	0,106886	5968,45	1,5001	1,4645	0,0669342	3737,55
**Spectrum 7**	UM	1	1	4/09/20	1,3136	0,253387	2,67068	1,5001	1,4645	0,383475	4,0418
**Spectrum 8**	UM	2	1	4/09/20	1,3136	0,187471	1,92362	1,5001	1,4645	0,365784	3,75328
**Spectrum 9**	UM	3	1	4/09/20	1,3136	0,217189	2,48175	1,5001	1,4645	0,263535	3,01133
**Spectrum 10**	UM	1	2	5/09/20	1,3136	0,25401	2,6222	1,5001	1,4645	0,375666	3,87808
**Spectrum 11**	UM	2	2	5/09/20	1,3136	0,156307	1,9813	1,5001	1,4645	0,277438	3,51674
**Spectrum 12**	UM	3	2	5/09/20	1,3136	0,191743	2,3987	1,5001	1,4645	0,247205	3,09252
**Spectrum 13**	FM	1	2	17/09/20	1,3136	0,125137	4741,26	1,5001	1,4645	0,0327814	1242,04
**Spectrum 14**	FM	1	1	17/09/20	1,3136	0,177782	5935,03	1,5001	1,4645	0,048794	1628,93
**Spectrum 15**	FM	2	1	17/09/20	1,3136	0,138077	4964,21	1,5001	1,4645	0,0335163	1205
**Spectrum 16**	FM	2	2	17/09/20	1,3136	0,0853963	3180,6	1,5001	1,4645	0,0260973	971,998
**Spectrum 17**	FM	3	1	17/09/20	1,3136	0,150576	4543,54	1,5001	1,4645	0,0577645	1743,01
**Spectrum 18**	FM	3	2	17/09/20	1,3136	0,178406	5424,45	1,5001	1,4645	0,0536286	1630,58
**Spectrum 19**	UM	0	3	20/12/19	1,3136	0,35009	10023	1,5001	1,4645	0,513937	14713,9
**Spectrum 20**	UM	0	2	20/12/19	1,3136	0,395622	13469,6	1,5001	1,4645	0,641644	21845,9
**Spectrum 21**	UM	0	1	20/12/19	1,3136	0,316124	10867,2	1,5001	1,4645	0,482853	16598,7
**Spectrum 22**	UUM	0	3	27/12/19	1,3136	0,0312465	10822,4	1,5001	1,4645	0,0296839	10281,2
**Spectrum 23**	UUM	0	2	27/12/19	1,3136	0,0336903	9607,84	1,5001	1,4645	0,0340334	9705,67
**Spectrum 24**	UUM	0	1	27/12/19	1,3136	0,031532	10797	1,5001	1,4645	0,032384	11088,7
**Spectrum 25**	FM	0	3	4/02/20	1,3136	0,144882	5897,94	1,5001	1,4645	0,0666153	2711,81
**Spectrum 26**	FM	0	2	28/12/19	1,3136	0,176864	8532,86	1,5001	1,4645	0,0903297	4357,98
**Spectrum 27**	FM	0	1	28/12/19	1,3136	0,24343	11137,4	1,5001	1,4645	0,152611	6982,25

	Method	Patient	Replicated	Date							
**Spectrum 1**	UUM	1	1	4/09/20	1,7974	1,6768	0,171918	9944,52	1,9177	1,8747	0,0422977
**Spectrum 2**	UUM	2	1	4/09/20	1,7974	1,6768	0,0731602	7591,35	1,9177	1,8747	0,0127885
**Spectrum 3**	UUM	3	1	4/09/20	1,7974	1,6768	0,0831983	6604,02	1,9177	1,8747	0,0172218
**Spectrum 4**	UUM	1	2	5/09/20	1,7974	1,6768	0,220281	11030,1	1,9177	1,8747	0,0500469
**Spectrum 5**	UUM	2	2	5/09/20	1,7974	1,6768	0,189391	8767,25	1,9177	1,8747	0,041074
**Spectrum 6**	UUM	3	2	5/09/20	1,7974	1,6768	0,121889	6806,18	1,9177	1,8747	0,0240108
**Spectrum 7**	UM	1	1	4/09/20	1,7974	1,6768	0,835674	8,80794	1,9177	1,8747	0,289513
**Spectrum 8**	UM	2	1	4/09/20	1,7974	1,6768	0,873586	8,9638	1,9177	1,8747	0,275218
**Spectrum 9**	UM	3	1	4/09/20	1,7974	1,6768	0,62291	7,11779	1,9177	1,8747	0,198097
**Spectrum 10**	UM	1	2	5/09/20	1,7974	1,6768	0,869144	8,97236	1,9177	1,8747	0,287423
**Spectrum 11**	UM	2	2	5/09/20	1,7974	1,6768	0,692558	8,77869	1,9177	1,8747	0,211582
**Spectrum 12**	UM	3	2	5/09/20	1,7974	1,6768	0,696993	8,71933	1,9177	1,8747	0,181252
**Spectrum 13**	FM	1	2	17/09/20	1,7974	1,6768	0,0685623	2597,74	1,9177	1,8747	0,0187632
**Spectrum 14**	FM	1	1	17/09/20	1,7974	1,6768	0,0946248	3158,93	1,9177	1,8747	0,0303842
**Spectrum 15**	FM	2	1	17/09/20	1,7974	1,6768	0,0595106	2139,56	1,9177	1,8747	0,0151317
**Spectrum 16**	FM	2	2	17/09/20	1,7974	1,6768	0,0581127	2164,41	1,9177	1,8747	0,0156417
**Spectrum 17**	FM	3	1	17/09/20	1,7974	1,6768	0,0626379	1890,06	1,9177	1,8747	0,0157295
**Spectrum 18**	FM	3	2	17/09/20	1,7974	1,6768	0,070918	2156,26	1,9177	1,8747	0,0177413
**Spectrum 19**	UM	0	3	20/12/19	1,7974	1,6768	1,00075	28651,1	1,9177	1,8747	0,352948
**Spectrum 20**	UM	0	2	20/12/19	1,7974	1,6768	1,13289	38571,3	1,9177	1,8747	0,419856
**Spectrum 21**	UM	0	1	20/12/19	1,7974	1,6768	0,903396	31055,5	1,9177	1,8747	0,322112
**Spectrum 22**	UUM	0	3	27/12/19	1,7974	1,6768	0,0462566	16021,2	1,9177	1,8747	0,01453
**Spectrum 23**	UUM	0	2	27/12/19	1,7974	1,6768	0,0479324	13669,4	1,9177	1,8747	0,015666
**Spectrum 24**	UUM	0	1	27/12/19	1,7974	1,6768	0,0479479	16418	1,9177	1,8747	0,0155219
**Spectrum 25**	FM	0	3	4/02/20	1,7974	1,6768	0,0858207	3493,63	1,9177	1,8747	0,0244059
**Spectrum 26**	FM	0	2	28/12/19	1,7974	1,6768	0,165586	7988,73	1,9177	1,8747	0,0478772
**Spectrum 27**	FM	0	1	28/12/19	1,7974	1,6768	0,319312	14609,2	1,9177	1,8747	0,0881614

	Method	Patient	Replicated	Date							
**Spectrum 1**	UUM	1	1	4/09/20	2446,7	1,9285	1,9179	0,0406238	2349,87	2,098	1,996
**Spectrum 2**	UUM	2	1	4/09/20	1326,98	1,9285	1,9179	0,0168281	1746,14	2,098	1,996
**Spectrum 3**	UUM	3	1	4/09/20	1367,01	1,9285	1,9179	0,0224319	1780,57	2,098	1,996
**Spectrum 4**	UUM	1	2	5/09/20	2505,99	1,9285	1,9179	0,0447277	2239,64	2,098	1,996
**Spectrum 5**	UUM	2	2	5/09/20	1901,38	1,9285	1,9179	0,0407093	1884,5	2,098	1,996
**Spectrum 6**	UUM	3	2	5/09/20	1340,74	1,9285	1,9179	0,0280185	1564,53	2,098	1,996
**Spectrum 7**	UM	1	1	4/09/20	3,05144	1,9285	1,9179	0,106173	1,11906	2,098	1,996
**Spectrum 8**	UM	2	1	4/09/20	2,82399	1,9285	1,9179	0,105596	1,08351	2,098	1,996
**Spectrum 9**	UM	3	1	4/09/20	2,2636	1,9285	1,9179	0,0819579	0,936507	2,098	1,996
**Spectrum 10**	UM	1	2	5/09/20	2,96714	1,9285	1,9179	0,106237	1,09671	2,098	1,996
**Spectrum 11**	UM	2	2	5/09/20	2,68196	1,9285	1,9179	0,0828294	1,04992	2,098	1,996
**Spectrum 12**	UM	3	2	5/09/20	2,26745	1,9285	1,9179	0,0724131	0,905883	2,098	1,996
**Spectrum 13**	FM	1	2	17/09/20	710,912	1,9285	1,9179	0,0260949	988,7	2,098	1,996
**Spectrum 14**	FM	1	1	17/09/20	1014,34	1,9285	1,9179	0,0312618	1043,64	2,098	1,996
**Spectrum 15**	FM	2	1	17/09/20	544,022	1,9285	1,9179	0,0261976	941,872	2,098	1,996
**Spectrum 16**	FM	2	2	17/09/20	582,576	1,9285	1,9179	0,025826	961,892	2,098	1,996
**Spectrum 17**	FM	3	1	17/09/20	474,627	1,9285	1,9179	0,0304855	919,883	2,098	1,996
**Spectrum 18**	FM	3	2	17/09/20	539,426	1,9285	1,9179	0,0308125	936,855	2,098	1,996
**Spectrum 19**	UM	0	3	20/12/19	10104,8	1,9285	1,9179	0,118312	3387,24	2,098	1,996
**Spectrum 20**	UM	0	2	20/12/19	14294,7	1,9285	1,9179	0,130678	4449,16	2,098	1,996
**Spectrum 21**	UM	0	1	20/12/19	11073	1,9285	1,9179	0,104197	3581,91	2,098	1,996
**Spectrum 22**	UUM	0	3	27/12/19	5032,54	1,9285	1,9179	0,00911637	3157,5	2,098	1,996
**Spectrum 23**	UUM	0	2	27/12/19	4467,64	1,9285	1,9179	0,0116234	3314,78	2,098	1,996
**Spectrum 24**	UUM	0	1	27/12/19	5314,93	1,9285	1,9179	0,00900152	3082,25	2,098	1,996
**Spectrum 25**	FM	0	3	4/02/20	993,53	1,9285	1,9179	0,0362738	1476,65	2,098	1,996
**Spectrum 26**	FM	0	2	28/12/19	2309,85	1,9285	1,9179	0,0432457	2086,4	2,098	1,996
**Spectrum 27**	FM	0	1	28/12/19	4033,57	1,9285	1,9179	0,0584652	2674,9	2,098	1,996

	Method	Patient	Replicated	Date							
**Spectrum 1**	UUM	1	1	4/09/20	0,0550366	3183,58	2,34	2,3302	0,00260215	150,521	2,4117
**Spectrum 2**	UUM	2	1	4/09/20	0,0198028	2054,81	2,34	2,3302	0,00084248	87,4183	2,4117
**Spectrum 3**	UUM	3	1	4/09/20	0,0338188	2684,43	2,34	2,3302	0,00113808	90,3369	2,4117
**Spectrum 4**	UUM	1	2	5/09/20	0,0573092	2869,63	2,34	2,3302	0,00137417	68,8086	2,4117
**Spectrum 5**	UUM	2	2	5/09/20	0,0546621	2530,4	2,34	2,3302	0,00175721	81,3444	2,4117
**Spectrum 6**	UUM	3	2	5/09/20	0,0558673	3119,58	2,34	2,3302	0,00203002	113,355	2,4117
**Spectrum 7**	UM	1	1	4/09/20	0,586871	6,18557	2,34	2,3302	0,0252954	0,266611	2,4117
**Spectrum 8**	UM	2	1	4/09/20	0,581233	5,96398	2,34	2,3302	0,0265239	0,27216	2,4117
**Spectrum 9**	UM	3	1	4/09/20	0,52181	5,96255	2,34	2,3302	0,0226144	0,258408	2,4117
**Spectrum 10**	UM	1	2	5/09/20	0,616717	6,3665	2,34	2,3302	0,0259142	0,267518	2,4117
**Spectrum 11**	UM	2	2	5/09/20	0,474414	6,01356	2,34	2,3302	0,0216145	0,27398	2,4117
**Spectrum 12**	UM	3	2	5/09/20	0,529075	6,61869	2,34	2,3302	0,0225821	0,282501	2,4117
**Spectrum 13**	FM	1	2	17/09/20	0,0809026	3065,29	2,34	2,3302	0,00242567	91,9056	2,4117
**Spectrum 14**	FM	1	1	17/09/20	0,11427	3814,77	2,34	2,3302	0,00529732	176,845	2,4117
**Spectrum 15**	FM	2	1	17/09/20	0,0691776	2487,11	2,34	2,3302	0,00243071	87,3902	2,4117
**Spectrum 16**	FM	2	2	17/09/20	0,0769189	2864,85	2,34	2,3302	0,00310988	115,828	2,4117
**Spectrum 17**	FM	3	1	17/09/20	0,114805	3464,18	2,34	2,3302	0,00250554	75,6031	2,4117
**Spectrum 18**	FM	3	2	17/09/20	0,10284	3126,86	2,34	2,3302	0,00359265	109,235	2,4117
**Spectrum 19**	UM	0	3	20/12/19	0,613902	17575,8	2,34	2,3302	0,0211236	604,763	2,4117
**Spectrum 20**	UM	0	2	20/12/19	0,682421	23234,2	2,34	2,3302	0,0257114	875,39	2,4117
**Spectrum 21**	UM	0	1	20/12/19	0,544656	18723,3	2,34	2,3302	0,0175797	604,327	2,4117
**Spectrum 22**	UUM	0	3	27/12/19	0,0122891	4256,4	2,34	2,3302	0,0006391	221,356	2,4117
**Spectrum 23**	UUM	0	2	27/12/19	0,0133616	3810,46	2,34	2,3302	0,00062911	179,409	2,4117
**Spectrum 24**	UUM	0	1	27/12/19	0,0142965	4895,33	2,34	2,3302	0,00055377	189,618	2,4117
**Spectrum 25**	FM	0	3	4/02/20	0,114089	4644,38	2,34	2,3302	0,00263065	107,09	2,4117
**Spectrum 26**	FM	0	2	28/12/19	0,136382	6579,79	2,34	2,3302	0,0026159	126,205	2,4117
**Spectrum 27**	FM	0	1	28/12/19	0,252789	11565,6	2,34	2,3302	0,0108334	495,649	2,4117

	Method	Patient	Replicated	Date							
**Spectrum 1**	UUM	1	1	4/09/20	2,4035	0,00383239	221,683	2,5612	2,5522	0,00389948	225,564
**Spectrum 2**	UUM	2	1	4/09/20	2,4035	0,00111422	115,615	2,5612	2,5522	0,00166243	172,499
**Spectrum 3**	UUM	3	1	4/09/20	2,4035	0,00110719	87,8856	2,5612	2,5522	0,00230636	183,072
**Spectrum 4**	UUM	1	2	5/09/20	2,4035	0,00194031	97,1566	2,5612	2,5522	0,00222958	111,641
**Spectrum 5**	UUM	2	2	5/09/20	2,4035	0,00134763	62,3842	2,5612	2,5522	0,00281123	130,137
**Spectrum 6**	UUM	3	2	5/09/20	2,4035	0,00107933	60,2689	2,5612	2,5522	0,00583463	325,801
**Spectrum 7**	UM	1	1	4/09/20	2,4035	0,011358	0,119713	2,5612	2,5522	0,014844	0,156455
**Spectrum 8**	UM	2	1	4/09/20	2,4035	0,0134242	0,137744	2,5612	2,5522	0,0139149	0,14278
**Spectrum 9**	UM	3	1	4/09/20	2,4035	0,012758	0,145782	2,5612	2,5522	0,0164201	0,187627
**Spectrum 10**	UM	1	2	5/09/20	2,4035	0,0123379	0,127367	2,5612	2,5522	0,0159896	0,165064
**Spectrum 11**	UM	2	2	5/09/20	2,4035	0,0109819	0,139204	2,5612	2,5522	0,0128441	0,162809
**Spectrum 12**	UM	3	2	5/09/20	2,4035	0,0128137	0,160298	2,5612	2,5522	0,0165943	0,207594
**Spectrum 13**	FM	1	2	17/09/20	2,4035	0,00354617	134,36	2,5612	2,5522	0,007312	277,042
**Spectrum 14**	FM	1	1	17/09/20	2,4035	0,00542531	181,117	2,5612	2,5522	0,0100626	335,928
**Spectrum 15**	FM	2	1	17/09/20	2,4035	0,00192076	69,0561	2,5612	2,5522	0,00658245	236,656
**Spectrum 16**	FM	2	2	17/09/20	2,4035	0,00315293	117,431	2,5612	2,5522	0,00682278	254,115
**Spectrum 17**	FM	3	1	17/09/20	2,4035	0,00226603	68,3762	2,5612	2,5522	0,0168498	508,434
**Spectrum 18**	FM	3	2	17/09/20	2,4035	0,00359671	109,358	2,5612	2,5522	0,0121224	368,583
**Spectrum 19**	UM	0	3	20/12/19	2,4035	0,0104901	300,329	2,5612	2,5522	0,0200201	573,17
**Spectrum 20**	UM	0	2	20/12/19	2,4035	0,0112191	381,974	2,5612	2,5522	0,0221206	753,133
**Spectrum 21**	UM	0	1	20/12/19	2,4035	0,00861505	296,154	2,5612	2,5522	0,0181509	623,963
**Spectrum 22**	UUM	0	3	27/12/19	2,4035	0,00024776	85,8123	2,5612	2,5522	0,00084164	291,506
**Spectrum 23**	UUM	0	2	27/12/19	2,4035	0,00027456	78,2978	2,5612	2,5522	0,00099517	283,804
**Spectrum 24**	UUM	0	1	27/12/19	2,4035	0,000232	79,4413	2,5612	2,5522	0,00105079	359,804
**Spectrum 25**	FM	0	3	4/02/20	2,4035	0,00451856	183,944	2,5612	2,5522	0,0169526	690,117
**Spectrum 26**	FM	0	2	28/12/19	2,4035	0,00367624	177,361	2,5612	2,5522	0,00923022	445,315
**Spectrum 27**	FM	0	1	28/12/19	2,4035	0,0061123	279,65	2,5612	2,5522	0,0170034	777,942

	Method	Patient	Replicated	Date								
**Spectrum 1**	UUM	1	1	4/09/20	2,6434	2,6285	0,0026322	152,259	2,5952	2,5224	0,0173707
**Spectrum 2**	UUM	2	1	4/09/20	2,6434	2,6285	0,00053709	55,7302	2,5952	2,5224	0,00716736
**Spectrum 3**	UUM	3	1	4/09/20	2,6434	2,6285	0,00128054	101,645	2,5952	2,5224	0,00936705
**Spectrum 4**	UUM	1	2	5/09/20	2,6434	2,6285	-0,0007318	-36,6448	2,5952	2,5224	0,00156396
**Spectrum 5**	UUM	2	2	5/09/20	2,6434	2,6285	0,0021898	101,37	2,5952	2,5224	0,00945377
**Spectrum 6**	UUM	3	2	5/09/20	2,6434	2,6285	0,00334512	186,789	2,5952	2,5224	0,020532
**Spectrum 7**	UM	1	1	4/09/20	2,6434	2,6285	0,0230665	0,243119	2,5952	2,5224	0,0774156
**Spectrum 8**	UM	2	1	4/09/20	2,6434	2,6285	0,0233621	0,239716	2,5952	2,5224	0,0870868
**Spectrum 9**	UM	3	1	4/09/20	2,6434	2,6285	0,0206437	0,235889	2,5952	2,5224	0,0933216
**Spectrum 10**	UM	1	2	5/09/20	2,6434	2,6285	0,0229408	0,236823	2,5952	2,5224	0,0839658
**Spectrum 11**	UM	2	2	5/09/20	2,6434	2,6285	0,0200449	0,254084	2,5952	2,5224	0,0758668
**Spectrum 12**	UM	3	2	5/09/20	2,6434	2,6285	0,0166307	0,208048	2,5952	2,5224	0,076305
**Spectrum 13**	FM	1	2	17/09/20	2,6434	2,6285	0,00192653	72,9939	2,5952	2,5224	0,0270019
**Spectrum 14**	FM	1	1	17/09/20	2,6434	2,6285	0,00452726	151,137	2,5952	2,5224	0,0395764
**Spectrum 15**	FM	2	1	17/09/20	2,6434	2,6285	0,00069211	24,883	2,5952	2,5224	0,017547
**Spectrum 16**	FM	2	2	17/09/20	2,6434	2,6285	0,00391662	145,875	2,5952	2,5224	0,0249443
**Spectrum 17**	FM	3	1	17/09/20	2,6434	2,6285	0,00117584	35,4802	2,5952	2,5224	0,0500021
**Spectrum 18**	FM	3	2	17/09/20	2,6434	2,6285	0,00090302	27,4565	2,5952	2,5224	0,0455563
**Spectrum 19**	UM	0	3	20/12/19	2,6434	2,6285	0,0156867	449,105	2,5952	2,5224	0,080928
**Spectrum 20**	UM	0	2	20/12/19	2,6434	2,6285	0,0168656	574,219	2,5952	2,5224	0,0937148
**Spectrum 21**	UM	0	1	20/12/19	2,6434	2,6285	0,012391	425,958	2,5952	2,5224	0,0700028
**Spectrum 22**	UUM	0	3	27/12/19	2,6434	2,6285	0,00038529	133,448	2,5952	2,5224	0,00326499
**Spectrum 23**	UUM	0	2	27/12/19	2,6434	2,6285	0,00032872	93,7437	2,5952	2,5224	0,00270235
**Spectrum 24**	UUM	0	1	27/12/19	2,6434	2,6285	0,00056006	191,772	2,5952	2,5224	0,00380932
**Spectrum 25**	FM	0	3	4/02/20	2,6434	2,6285	0,0014716	59,9067	2,5952	2,5224	0,0551701
**Spectrum 26**	FM	0	2	28/12/19	2,6434	2,6285	0,00119074	57,4475	2,5952	2,5224	0,0317814
**Spectrum 27**	FM	0	1	28/12/19	2,6434	2,6285	0,00518952	237,431	2,5952	2,5224	0,0648026

	Method	Patient	Replicated	Date								
**Spectrum 1**	UUM	1	1	4/09/20	1004,8	2,663	2,6435	0,0107404	621,278	3,8593	3,8349
**Spectrum 2**	UUM	2	1	4/09/20	743,71	2,663	2,6435	0,00454427	471,529	3,8593	3,8349
**Spectrum 3**	UUM	3	1	4/09/20	743,527	2,663	2,6435	0,00787925	625,43	3,8593	3,8349
**Spectrum 4**	UUM	1	2	5/09/20	78,3117	2,663	2,6435	0,00883885	442,585	3,8593	3,8349
**Spectrum 5**	UUM	2	2	5/09/20	437,631	2,663	2,6435	0,0110366	510,903	3,8593	3,8349
**Spectrum 6**	UUM	3	2	5/09/20	1146,49	2,663	2,6435	0,0130349	727,86	3,8593	3,8349
**Spectrum 7**	UM	1	1	4/09/20	0,815954	2,663	2,6435	0,0443582	0,467532	3,8593	3,8349
**Spectrum 8**	UM	2	1	4/09/20	0,893591	2,663	2,6435	0,0444529	0,456127	3,8593	3,8349
**Spectrum 9**	UM	3	1	4/09/20	1,06636	2,663	2,6435	0,0454002	0,518773	3,8593	3,8349
**Spectrum 10**	UM	1	2	5/09/20	0,866797	2,663	2,6435	0,0441612	0,455886	3,8593	3,8349
**Spectrum 11**	UM	2	2	5/09/20	0,961669	2,663	2,6435	0,0387807	0,491574	3,8593	3,8349
**Spectrum 12**	UM	3	2	5/09/20	0,95457	2,663	2,6435	0,0391567	0,489847	3,8593	3,8349
**Spectrum 13**	FM	1	2	17/09/20	1023,07	2,663	2,6435	0,0109421	414,582	3,8593	3,8349
**Spectrum 14**	FM	1	1	17/09/20	1321,21	2,663	2,6435	0,0161664	539,697	3,8593	3,8349
**Spectrum 15**	FM	2	1	17/09/20	630,859	2,663	2,6435	0,00937674	337,118	3,8593	3,8349
**Spectrum 16**	FM	2	2	17/09/20	929,051	2,663	2,6435	0,0116368	433,413	3,8593	3,8349
**Spectrum 17**	FM	3	1	17/09/20	1508,79	2,663	2,6435	0,017346	523,405	3,8593	3,8349
**Spectrum 18**	FM	3	2	17/09/20	1385,14	2,663	2,6435	0,0166385	505,895	3,8593	3,8349
**Spectrum 19**	UM	0	3	20/12/19	2316,94	2,663	2,6435	0,0385809	1104,56	3,8593	3,8349
**Spectrum 20**	UM	0	2	20/12/19	3190,68	2,663	2,6435	0,043389	1477,25	3,8593	3,8349
**Spectrum 21**	UM	0	1	20/12/19	2406,44	2,663	2,6435	0,0328433	1129,03	3,8593	3,8349
**Spectrum 22**	UUM	0	3	27/12/19	1130,85	2,663	2,6435	0,00200535	694,562	3,8593	3,8349
**Spectrum 23**	UUM	0	2	27/12/19	770,658	2,663	2,6435	0,00222455	634,399	3,8593	3,8349
**Spectrum 24**	UUM	0	1	27/12/19	1304,36	2,663	2,6435	0,00241566	827,156	3,8593	3,8349
**Spectrum 25**	FM	0	3	4/02/20	2245,89	2,663	2,6435	0,0148092	602,862	3,8593	3,8349
**Spectrum 26**	FM	0	2	28/12/19	1533,3	2,663	2,6435	0,0117989	569,24	3,8593	3,8349
**Spectrum 27**	FM	0	1	28/12/19	2964,85	2,663	2,6435	0,0190356	870,918	3,8593	3,8349

	Method	Patient	Replicated	Date							
**Spectrum 1**	UUM	1	1	4/09/20	0,0255117	1475,72	2,7038	2,6726	0,00966047	558,807	2,673
**Spectrum 2**	UUM	2	1	4/09/20	0,0133514	1385,38	2,7038	2,6726	0,00541569	561,951	2,673
**Spectrum 3**	UUM	3	1	4/09/20	0,0252586	2004,95	2,7038	2,6726	0,00871221	691,548	2,673
**Spectrum 4**	UUM	1	2	5/09/20	0,0208313	1043,08	2,7038	2,6726	0,00399909	200,245	2,673
**Spectrum 5**	UUM	2	2	5/09/20	0,0256603	1187,86	2,7038	2,6726	0,0120437	557,523	2,673
**Spectrum 6**	UUM	3	2	5/09/20	0,0309186	1726,47	2,7038	2,6726	0,0113759	635,222	2,673
**Spectrum 7**	UM	1	1	4/09/20	0,105058	1,10731	2,7038	2,6726	0,0502025	0,52913	2,673
**Spectrum 8**	UM	2	1	4/09/20	0,110407	1,13288	2,7038	2,6726	0,0566601	0,581384	2,673
**Spectrum 9**	UM	3	1	4/09/20	0,116538	1,33164	2,7038	2,6726	0,0538792	0,615661	2,673
**Spectrum 10**	UM	1	2	5/09/20	0,101868	1,0516	2,7038	2,6726	0,0513462	0,530058	2,673
**Spectrum 11**	UM	2	2	5/09/20	0,0871285	1,10442	2,7038	2,6726	0,0497105	0,630118	2,673
**Spectrum 12**	UM	3	2	5/09/20	0,102968	1,28812	2,7038	2,6726	0,0483879	0,605329	2,673
**Spectrum 13**	FM	1	2	17/09/20	0,0191545	725,737	2,7038	2,6726	0,0128748	487,808	2,673
**Spectrum 14**	FM	1	1	17/09/20	0,029159	973,439	2,7038	2,6726	0,015535	518,616	2,673
**Spectrum 15**	FM	2	1	17/09/20	0,0173806	624,877	2,7038	2,6726	0,0100866	362,639	2,673
**Spectrum 16**	FM	2	2	17/09/20	0,0193166	719,447	2,7038	2,6726	0,0158987	592,147	2,673
**Spectrum 17**	FM	3	1	17/09/20	0,0364035	1098,45	2,7038	2,6726	0,0210239	634,385	2,673
**Spectrum 18**	FM	3	2	17/09/20	0,0362567	1102,39	2,7038	2,6726	0,0180852	549,881	2,673
**Spectrum 19**	UM	0	3	20/12/19	0,0556821	1594,16	2,7038	2,6726	0,0339711	972,581	2,673
**Spectrum 20**	UM	0	2	20/12/19	0,0874754	2978,25	2,7038	2,6726	0,0407688	1388,04	2,673
**Spectrum 21**	UM	0	1	20/12/19	0,062245	2139,76	2,7038	2,6726	0,0321474	1105,11	2,673
**Spectrum 22**	UUM	0	3	27/12/19	0,00164237	568,842	2,7038	2,6726	0,00141594	490,418	2,673
**Spectrum 23**	UUM	0	2	27/12/19	0,00146406	417,52	2,7038	2,6726	0,00122948	350,625	2,673
**Spectrum 24**	UUM	0	1	27/12/19	0,00157366	538,844	2,7038	2,6726	0,00163391	559,473	2,673
**Spectrum 25**	FM	0	3	4/02/20	0,0398641	1622,81	2,7038	2,6726	0,0124332	506,136	2,673
**Spectrum 26**	FM	0	2	28/12/19	0,0311325	1502	2,7038	2,6726	0,0116294	561,063	2,673
**Spectrum 27**	FM	0	1	28/12/19	0,0663627	3036,23	2,7038	2,6726	0,0238819	1092,65	2,673

	Method	Patient	Replicated	Date							
**Spectrum 1**	UUM	1	1	4/09/20	2,6631	0,00387622	224,219	2,7196	2,7089	0,0004905	28,373
**Spectrum 2**	UUM	2	1	4/09/20	2,6631	0,00203222	210,87	2,7196	2,7089	-5,95E-05	-6,17905
**Spectrum 3**	UUM	3	1	4/09/20	2,6631	0,00388686	308,527	2,7196	2,7089	-0,0006758	-53,642
**Spectrum 4**	UUM	1	2	5/09/20	2,6631	0,00199562	99,9261	2,7196	2,7089	-0,0013157	-65,8813
**Spectrum 5**	UUM	2	2	5/09/20	2,6631	0,00441488	204,372	2,7196	2,7089	-0,0005067	-23,4559
**Spectrum 6**	UUM	3	2	5/09/20	2,6631	0,00489241	273,188	2,7196	2,7089	-0,0022547	-125,903
**Spectrum 7**	UM	1	1	4/09/20	2,6631	0,0162076	0,170827	2,7196	2,7089	0,0150038	0,158139
**Spectrum 8**	UM	2	1	4/09/20	2,6631	0,019969	0,204901	2,7196	2,7089	0,0168139	0,172526
**Spectrum 9**	UM	3	1	4/09/20	2,6631	0,018659	0,21321	2,7196	2,7089	0,0137495	0,157111
**Spectrum 10**	UM	1	2	5/09/20	2,6631	0,0182802	0,18871	2,7196	2,7089	0,0146859	0,151606
**Spectrum 11**	UM	2	2	5/09/20	2,6631	0,0163027	0,206649	2,7196	2,7089	0,0146972	0,186299
**Spectrum 12**	UM	3	2	5/09/20	2,6631	0,0177849	0,222488	2,7196	2,7089	0,0112421	0,140638
**Spectrum 13**	FM	1	2	17/09/20	2,6631	0,00467224	177,025	2,7196	2,7089	0,0007515	28,4735
**Spectrum 14**	FM	1	1	17/09/20	2,6631	0,00745679	248,936	2,7196	2,7089	0,00087583	29,2385
**Spectrum 15**	FM	2	1	17/09/20	2,6631	0,00479873	172,527	2,7196	2,7089	-0,0009383	-33,7336
**Spectrum 16**	FM	2	2	17/09/20	2,6631	0,00632129	235,437	2,7196	2,7089	3,13E-05	1,1641
**Spectrum 17**	FM	3	1	17/09/20	2,6631	0,0108127	326,266	2,7196	2,7089	-0,0008088	-24,4058
**Spectrum 18**	FM	3	2	17/09/20	2,6631	0,0078356	238,242	2,7196	2,7089	-0,000322	-9,7913
**Spectrum 19**	UM	0	3	20/12/19	2,6631	0,0108118	309,539	2,7196	2,7089	0,0106276	304,265
**Spectrum 20**	UM	0	2	20/12/19	2,6631	0,0149533	509,11	2,7196	2,7089	0,012806	436,002
**Spectrum 21**	UM	0	1	20/12/19	2,6631	0,011937	410,351	2,7196	2,7089	0,00834496	286,87
**Spectrum 22**	UUM	0	3	27/12/19	2,6631	0,00059596	206,415	2,7196	2,7089	2,32E-05	8,02186
**Spectrum 23**	UUM	0	2	27/12/19	2,6631	0,00080998	230,989	2,7196	2,7089	1,36E-05	3,89148
**Spectrum 24**	UUM	0	1	27/12/19	2,6631	0,00065212	223,294	2,7196	2,7089	0,00015482	53,0133
**Spectrum 25**	FM	0	3	4/02/20	2,6631	0,00616771	251,078	2,7196	2,7089	-3,90E-05	-1,58841
**Spectrum 26**	FM	0	2	28/12/19	2,6631	0,00424241	204,676	2,7196	2,7089	0,00040296	19,4408
**Spectrum 27**	FM	0	1	28/12/19	2,6631	0,00855629	391,468	2,7196	2,7089	0,00319765	146,299

	Method	Patient	Replicated	Date							
**Spectrum 1**	UUM	1	1	4/09/20	2,8541	2,7938	0,015676	906,774	3,9218	3,9141	0,00625218
**Spectrum 2**	UUM	2	1	4/09/20	2,8541	2,7938	0,00704263	730,768	3,9218	3,9141	0,00426827
**Spectrum 3**	UUM	3	1	4/09/20	2,8541	2,7938	0,0109917	872,486	3,9218	3,9141	0,0064694
**Spectrum 4**	UUM	1	2	5/09/20	2,8541	2,7938	-0,0038213	-191,342	3,9218	3,9141	0,00398899
**Spectrum 5**	UUM	2	2	5/09/20	2,8541	2,7938	0,0139982	648	3,9218	3,9141	0,00614411
**Spectrum 6**	UUM	3	2	5/09/20	2,8541	2,7938	0,0186212	1039,79	3,9218	3,9141	0,00908881
**Spectrum 7**	UM	1	1	4/09/20	2,8541	2,7938	0,0632534	0,666686	3,9218	3,9141	0,0283623
**Spectrum 8**	UM	2	1	4/09/20	2,8541	2,7938	0,0813812	0,835045	3,9218	3,9141	0,0296024
**Spectrum 9**	UM	3	1	4/09/20	2,8541	2,7938	0,0703644	0,804032	3,9218	3,9141	0,0306089
**Spectrum 10**	UM	1	2	5/09/20	2,8541	2,7938	0,0658384	0,679665	3,9218	3,9141	0,0293251
**Spectrum 11**	UM	2	2	5/09/20	2,8541	2,7938	0,0693907	0,87958	3,9218	3,9141	0,0237989
**Spectrum 12**	UM	3	2	5/09/20	2,8541	2,7938	0,0520839	0,651565	3,9218	3,9141	0,0208316
**Spectrum 13**	FM	1	2	17/09/20	2,8541	2,7938	0,0120947	458,254	3,9218	3,9141	0,0075068
**Spectrum 14**	FM	1	1	17/09/20	2,8541	2,7938	0,0220162	734,984	3,9218	3,9141	0,00777929
**Spectrum 15**	FM	2	1	17/09/20	2,8541	2,7938	0,00775913	278,961	3,9218	3,9141	0,00738915
**Spectrum 16**	FM	2	2	17/09/20	2,8541	2,7938	0,0191495	713,225	3,9218	3,9141	0,00769887
**Spectrum 17**	FM	3	1	17/09/20	2,8541	2,7938	0,0197308	595,365	3,9218	3,9141	0,00987497
**Spectrum 18**	FM	3	2	17/09/20	2,8541	2,7938	0,0227591	691,992	3,9218	3,9141	0,00840741
**Spectrum 19**	UM	0	3	20/12/19	2,8541	2,7938	0,0378192	1082,75	3,9218	3,9141	0,0179122
**Spectrum 20**	UM	0	2	20/12/19	2,8541	2,7938	0,0474593	1615,83	3,9218	3,9141	0,0215665
**Spectrum 21**	UM	0	1	20/12/19	2,8541	2,7938	0,0319305	1097,65	3,9218	3,9141	0,0162223
**Spectrum 22**	UUM	0	3	27/12/19	2,8541	2,7938	0,00166373	576,242	3,9218	3,9141	0,00052088
**Spectrum 23**	UUM	0	2	27/12/19	2,8541	2,7938	0,00157938	450,408	3,9218	3,9141	0,00053175
**Spectrum 24**	UUM	0	1	27/12/19	2,8541	2,7938	0,0021719	743,688	3,9218	3,9141	0,00044388
**Spectrum 25**	FM	0	3	4/02/20	2,8541	2,7938	0,00882384	359,205	3,9218	3,9141	0,00953612
**Spectrum 26**	FM	0	2	28/12/19	2,8541	2,7938	0,00403628	194,731	3,9218	3,9141	0,00779686
**Spectrum 27**	FM	0	1	28/12/19	2,8541	2,7938	0,0151841	694,703	3,9218	3,9141	0,0146835

	Method	Patient	Replicated	Date							
**Spectrum 1**	UUM	1	1	4/09/20	361,656	3,9138	3,8929	0,0230266	1331,97	3,1376	3,1285
**Spectrum 2**	UUM	2	1	4/09/20	442,89	3,9138	3,8929	0,0147896	1534,62	3,1376	3,1285
**Spectrum 3**	UUM	3	1	4/09/20	513,521	3,9138	3,8929	0,0189063	1500,72	3,1376	3,1285
**Spectrum 4**	UUM	1	2	5/09/20	199,74	3,9138	3,8929	0,0139424	698,133	3,1376	3,1285
**Spectrum 5**	UUM	2	2	5/09/20	284,421	3,9138	3,8929	0,0219797	1017,48	3,1376	3,1285
**Spectrum 6**	UUM	3	2	5/09/20	507,511	3,9138	3,8929	0,0320213	1788,04	3,1376	3,1285
**Spectrum 7**	UM	1	1	4/09/20	0,298936	3,9138	3,8929	0,0930004	0,980217	3,1376	3,1285
**Spectrum 8**	UM	2	1	4/09/20	0,303748	3,9138	3,8929	0,0963585	0,988727	3,1376	3,1285
**Spectrum 9**	UM	3	1	4/09/20	0,349759	3,9138	3,8929	0,0957377	1,09396	3,1376	3,1285
**Spectrum 10**	UM	1	2	5/09/20	0,302729	3,9138	3,8929	0,094686	0,977465	3,1376	3,1285
**Spectrum 11**	UM	2	2	5/09/20	0,301669	3,9138	3,8929	0,0764616	0,969208	3,1376	3,1285
**Spectrum 12**	UM	3	2	5/09/20	0,260602	3,9138	3,8929	0,0720677	0,901561	3,1376	3,1285
**Spectrum 13**	FM	1	2	17/09/20	284,423	3,9138	3,8929	0,0224807	851,765	3,1376	3,1285
**Spectrum 14**	FM	1	1	17/09/20	259,702	3,9138	3,8929	0,0340438	1136,51	3,1376	3,1285
**Spectrum 15**	FM	2	1	17/09/20	265,659	3,9138	3,8929	0,0223906	805,001	3,1376	3,1285
**Spectrum 16**	FM	2	2	17/09/20	286,745	3,9138	3,8929	0,0278708	1038,05	3,1376	3,1285
**Spectrum 17**	FM	3	1	17/09/20	297,972	3,9138	3,8929	0,0425069	1282,62	3,1376	3,1285
**Spectrum 18**	FM	3	2	17/09/20	255,627	3,9138	3,8929	0,0384757	1169,86	3,1376	3,1285
**Spectrum 19**	UM	0	3	20/12/19	512,821	3,9138	3,8929	0,056873	1628,26	3,1376	3,1285
**Spectrum 20**	UM	0	2	20/12/19	734,268	3,9138	3,8929	0,0712794	2426,83	3,1376	3,1285
**Spectrum 21**	UM	0	1	20/12/19	557,664	3,9138	3,8929	0,0552017	1897,63	3,1376	3,1285
**Spectrum 22**	UUM	0	3	27/12/19	180,408	3,9138	3,8929	0,00170593	590,858	3,1376	3,1285
**Spectrum 23**	UUM	0	2	27/12/19	151,645	3,9138	3,8929	0,00193539	551,936	3,1376	3,1285
**Spectrum 24**	UUM	0	1	27/12/19	151,99	3,9138	3,8929	0,0016008	548,137	3,1376	3,1285
**Spectrum 25**	FM	0	3	4/02/20	388,201	3,9138	3,8929	0,0309033	1258,03	3,1376	3,1285
**Spectrum 26**	FM	0	2	28/12/19	376,162	3,9138	3,8929	0,0241143	1163,4	3,1376	3,1285
**Spectrum 27**	FM	0	1	28/12/19	671,802	3,9138	3,8929	0,0498174	2279,25	3,1376	3,1285

	Method	Patient	Replicated	Date							
**Spectrum 1**	UUM	1	1	4/09/20	0,00523674	302,918	3,1671	3,1378	0,0109349	632,527	3,3122
**Spectrum 2**	UUM	2	1	4/09/20	0,00230482	239,156	3,1671	3,1378	0,00363213	376,882	3,3122
**Spectrum 3**	UUM	3	1	4/09/20	0,00347735	276,021	3,1671	3,1378	0,027444	2178,42	3,3122
**Spectrum 4**	UUM	1	2	5/09/20	0,00088438	44,2835	3,1671	3,1378	-0,0019268	-96,4795	3,3122
**Spectrum 5**	UUM	2	2	5/09/20	0,00388716	179,943	3,1671	3,1378	0,00565636	261,843	3,3122
**Spectrum 6**	UUM	3	2	5/09/20	0,00563865	314,857	3,1671	3,1378	0,0399225	2229,24	3,3122
**Spectrum 7**	UM	1	1	4/09/20	0,0194073	0,204552	3,1671	3,1378	0,0458963	0,483744	3,3122
**Spectrum 8**	UM	2	1	4/09/20	0,0216875	0,222534	3,1671	3,1378	0,0558251	0,572816	3,3122
**Spectrum 9**	UM	3	1	4/09/20	0,0204269	0,233412	3,1671	3,1378	0,0954458	1,09063	3,3122
**Spectrum 10**	UM	1	2	5/09/20	0,0183809	0,18975	3,1671	3,1378	0,0474483	0,48982	3,3122
**Spectrum 11**	UM	2	2	5/09/20	0,0175107	0,221961	3,1671	3,1378	0,042631	0,54038	3,3122
**Spectrum 12**	UM	3	2	5/09/20	0,0161875	0,202504	3,1671	3,1378	0,0825743	1,033	3,3122
**Spectrum 13**	FM	1	2	17/09/20	0,00079884	30,2669	3,1671	3,1378	0,00388084	147,04	3,3122
**Spectrum 14**	FM	1	1	17/09/20	0,00167103	55,7854	3,1671	3,1378	0,00970407	323,959	3,3122
**Spectrum 15**	FM	2	1	17/09/20	-0,0003974	-14,2881	3,1671	3,1378	-0,0023816	-85,6236	3,3122
**Spectrum 16**	FM	2	2	17/09/20	0,00201023	74,8714	3,1671	3,1378	0,00455813	169,768	3,3122
**Spectrum 17**	FM	3	1	17/09/20	0,00249235	75,2053	3,1671	3,1378	0,0666934	2012,44	3,3122
**Spectrum 18**	FM	3	2	17/09/20	0,0021635	65,7813	3,1671	3,1378	0,0525943	1599,13	3,3122
**Spectrum 19**	UM	0	3	20/12/19	0,0205374	587,98	3,1671	3,1378	0,0398996	1142,31	3,3122
**Spectrum 20**	UM	0	2	20/12/19	0,0239567	815,648	3,1671	3,1378	0,049736	1693,35	3,3122
**Spectrum 21**	UM	0	1	20/12/19	0,0197127	677,653	3,1671	3,1378	0,03595	1235,83	3,3122
**Spectrum 22**	UUM	0	3	27/12/19	0,00130459	451,853	3,1671	3,1378	0,00229852	796,103	3,3122
**Spectrum 23**	UUM	0	2	27/12/19	0,00138305	394,419	3,1671	3,1378	0,00214271	611,06	3,3122
**Spectrum 24**	UUM	0	1	27/12/19	0,00136283	466,652	3,1671	3,1378	0,00255805	875,914	3,3122
**Spectrum 25**	FM	0	3	4/02/20	0,00389203	158,439	3,1671	3,1378	0,00906282	368,934	3,3122
**Spectrum 26**	FM	0	2	28/12/19	0,00273786	132,089	3,1671	3,1378	0,00627152	302,571	3,3122
**Spectrum 27**	FM	0	1	28/12/19	0,00677821	310,117	3,1671	3,1378	0,014558	666,059	3,3122

	Method	Patient	Replicated	Date							
**Spectrum 1**	UUM	1	1	4/09/20	3,2904	0,0112579	651,209	4,0133	4,0054	0,006747	390,278
**Spectrum 2**	UUM	2	1	4/09/20	3,2904	0,00302139	313,51	4,0133	4,0054	0,00318614	330,605
**Spectrum 3**	UUM	3	1	4/09/20	3,2904	0,00753165	597,839	4,0133	4,0054	0,0037521	297,83
**Spectrum 4**	UUM	1	2	5/09/20	3,2904	0,00943907	472,64	4,0133	4,0054	0,00606686	303,784
**Spectrum 5**	UUM	2	2	5/09/20	3,2904	0,00698709	323,444	4,0133	4,0054	0,00528436	244,622
**Spectrum 6**	UUM	3	2	5/09/20	3,2904	0,0115251	643,552	4,0133	4,0054	0,00677612	378,373
**Spectrum 7**	UM	1	1	4/09/20	3,2904	0,025361	0,267303	4,0133	4,0054	0,0270211	0,2848
**Spectrum 8**	UM	2	1	4/09/20	3,2904	0,0283081	0,290467	4,0133	4,0054	0,0295235	0,302938
**Spectrum 9**	UM	3	1	4/09/20	3,2904	0,030373	0,347062	4,0133	4,0054	0,0260586	0,297763
**Spectrum 10**	UM	1	2	5/09/20	3,2904	0,022899	0,236391	4,0133	4,0054	0,0274666	0,283544
**Spectrum 11**	UM	2	2	5/09/20	3,2904	0,0224824	0,284981	4,0133	4,0054	0,0195665	0,24802
**Spectrum 12**	UM	3	2	5/09/20	3,2904	0,0256862	0,321333	4,0133	4,0054	0,0185611	0,232198
**Spectrum 13**	FM	1	2	17/09/20	3,2904	0,00884186	335,007	4,0133	4,0054	0,00789243	299,034
**Spectrum 14**	FM	1	1	17/09/20	3,2904	0,0110615	369,274	4,0133	4,0054	0,0146755	489,923
**Spectrum 15**	FM	2	1	17/09/20	3,2904	0,00298711	107,394	4,0133	4,0054	0,00477742	171,76
**Spectrum 16**	FM	2	2	17/09/20	3,2904	0,00891609	332,081	4,0133	4,0054	0,00836671	311,619
**Spectrum 17**	FM	3	1	17/09/20	3,2904	0,0136972	413,305	4,0133	4,0054	0,00563649	170,078
**Spectrum 18**	FM	3	2	17/09/20	3,2904	0,014036	426,765	4,0133	4,0054	0,0075044	228,171
**Spectrum 19**	UM	0	3	20/12/19	3,2904	0,0235138	673,192	4,0133	4,0054	0,00394679	112,995
**Spectrum 20**	UM	0	2	20/12/19	3,2904	0,0326949	1113,15	4,0133	4,0054	0,00082524	28,0967
**Spectrum 21**	UM	0	1	20/12/19	3,2904	0,0216593	744,568	4,0133	4,0054	0,00050911	17,5013
**Spectrum 22**	UUM	0	3	27/12/19	3,2904	0,00281906	976,394	4,0133	4,0054	0,00019337	66,976
**Spectrum 23**	UUM	0	2	27/12/19	3,2904	0,00217649	620,693	4,0133	4,0054	0,00019247	54,888
**Spectrum 24**	UUM	0	1	27/12/19	3,2904	0,00213792	732,054	4,0133	4,0054	0,00017651	60,4397
**Spectrum 25**	FM	0	3	4/02/20	3,2904	0,0118936	484,17	4,0133	4,0054	-0,0004209	-17,1334
**Spectrum 26**	FM	0	2	28/12/19	3,2904	0,0108006	521,078	4,0133	4,0054	0,00056151	27,0904
**Spectrum 27**	FM	0	1	28/12/19	3,2904	0,0272824	1248,23	4,0133	4,0054	0,0021209	97,0356

	Method	Patient	Replicated	Date							
**Spectrum 1**	UUM	1	1	4/09/20	3,2082	3,1984	0,021125	1221,97	3,2143	3,2082	0,00374227
**Spectrum 2**	UUM	2	1	4/09/20	3,2082	3,1984	0,00781344	810,749	3,2143	3,2082	0,00110862
**Spectrum 3**	UUM	3	1	4/09/20	3,2082	3,1984	0,0211903	1682,02	3,2143	3,2082	0,00102287
**Spectrum 4**	UUM	1	2	5/09/20	3,2082	3,1984	0,0207612	1039,57	3,2143	3,2082	0,00168213
**Spectrum 5**	UUM	2	2	5/09/20	3,2082	3,1984	0,018429	853,11	3,2143	3,2082	0,00353343
**Spectrum 6**	UUM	3	2	5/09/20	3,2082	3,1984	0,0325663	1818,48	3,2143	3,2082	0,00264144
**Spectrum 7**	UM	1	1	4/09/20	3,2082	3,1984	0,0537257	0,566264	3,2143	3,2082	0,0186101
**Spectrum 8**	UM	2	1	4/09/20	3,2082	3,1984	0,044231	0,45385	3,2143	3,2082	0,0197317
**Spectrum 9**	UM	3	1	4/09/20	3,2082	3,1984	0,0619183	0,707521	3,2143	3,2082	0,0157192
**Spectrum 10**	UM	1	2	5/09/20	3,2082	3,1984	0,0572129	0,590622	3,2143	3,2082	0,0198678
**Spectrum 11**	UM	2	2	5/09/20	3,2082	3,1984	0,0388095	0,491939	3,2143	3,2082	0,016992
**Spectrum 12**	UM	3	2	5/09/20	3,2082	3,1984	0,0538745	0,673966	3,2143	3,2082	0,0180063
**Spectrum 13**	FM	1	2	17/09/20	3,2082	3,1984	0,0223067	845,174	3,2143	3,2082	0,00278938
**Spectrum 14**	FM	1	1	17/09/20	3,2082	3,1984	0,0260181	868,585	3,2143	3,2082	0,00441144
**Spectrum 15**	FM	2	1	17/09/20	3,2082	3,1984	0,0118063	424,466	3,2143	3,2082	0,00075838
**Spectrum 16**	FM	2	2	17/09/20	3,2082	3,1984	0,0144044	536,495	3,2143	3,2082	0,00351764
**Spectrum 17**	FM	3	1	17/09/20	3,2082	3,1984	0,0504772	1523,12	3,2143	3,2082	0,00198331
**Spectrum 18**	FM	3	2	17/09/20	3,2082	3,1984	0,0427812	1300,76	3,2143	3,2082	0,00276897
**Spectrum 19**	UM	0	3	20/12/19	3,2082	3,1984	0,0206531	591,29	3,2143	3,2082	0,00891849
**Spectrum 20**	UM	0	2	20/12/19	3,2082	3,1984	0,0208084	708,457	3,2143	3,2082	0,00959115
**Spectrum 21**	UM	0	1	20/12/19	3,2082	3,1984	0,0184149	633,036	3,2143	3,2082	0,00785613
**Spectrum 22**	UUM	0	3	27/12/19	3,2082	3,1984	0,00148432	514,101	3,2143	3,2082	0,0005271
**Spectrum 23**	UUM	0	2	27/12/19	3,2082	3,1984	0,00181653	518,038	3,2143	3,2082	0,00058071
**Spectrum 24**	UUM	0	1	27/12/19	3,2082	3,1984	0,00171051	585,701	3,2143	3,2082	0,000632
**Spectrum 25**	FM	0	3	4/02/20	3,2082	3,1984	0,0080809	328,961	3,2143	3,2082	0,00110596
**Spectrum 26**	FM	0	2	28/12/19	3,2082	3,1984	0,00813047	392,257	3,2143	3,2082	0,00293552
**Spectrum 27**	FM	0	1	28/12/19	3,2082	3,1984	0,0146549	670,491	3,2143	3,2082	0,00440536

	Method	Patient	Replicated	Date							
**Spectrum 1**	UUM	1	1	4/09/20	216,47	3,2245	3,2146	0,0310822	1797,94	3,2343	3,2247
**Spectrum 2**	UUM	2	1	4/09/20	115,034	3,2245	3,2146	0,0162282	1683,89	3,2343	3,2247
**Spectrum 3**	UUM	3	1	4/09/20	81,1923	3,2245	3,2146	0,00911457	723,486	3,2343	3,2247
**Spectrum 4**	UUM	1	2	5/09/20	84,2287	3,2245	3,2146	0,0315667	1580,63	3,2343	3,2247
**Spectrum 5**	UUM	2	2	5/09/20	163,569	3,2245	3,2146	0,0366753	1697,76	3,2343	3,2247
**Spectrum 6**	UUM	3	2	5/09/20	147,496	3,2245	3,2146	0,0201228	1123,64	3,2343	3,2247
**Spectrum 7**	UM	1	1	4/09/20	0,196149	3,2245	3,2146	0,0793069	0,835889	3,2343	3,2247
**Spectrum 8**	UM	2	1	4/09/20	0,202465	3,2245	3,2146	0,0809487	0,830608	3,2343	3,2247
**Spectrum 9**	UM	3	1	4/09/20	0,179619	3,2245	3,2146	0,0499525	0,570791	3,2343	3,2247
**Spectrum 10**	UM	1	2	5/09/20	0,2051	3,2245	3,2146	0,084241	0,869639	3,2343	3,2247
**Spectrum 11**	UM	2	2	5/09/20	0,215386	3,2245	3,2146	0,0691812	0,876923	3,2343	3,2247
**Spectrum 12**	UM	3	2	5/09/20	0,225257	3,2245	3,2146	0,0456404	0,570958	3,2343	3,2247
**Spectrum 13**	FM	1	2	17/09/20	105,686	3,2245	3,2146	0,0437951	1659,34	3,2343	3,2247
**Spectrum 14**	FM	1	1	17/09/20	147,271	3,2245	3,2146	0,0544578	1818,01	3,2343	3,2247
**Spectrum 15**	FM	2	1	17/09/20	27,2656	3,2245	3,2146	0,0408869	1469,99	3,2343	3,2247
**Spectrum 16**	FM	2	2	17/09/20	131,015	3,2245	3,2146	0,0443114	1650,38	3,2343	3,2247
**Spectrum 17**	FM	3	1	17/09/20	59,8452	3,2245	3,2146	0,0262269	791,383	3,2343	3,2247
**Spectrum 18**	FM	3	2	17/09/20	84,1907	3,2245	3,2146	0,0253575	770,996	3,2343	3,2247
**Spectrum 19**	UM	0	3	20/12/19	255,333	3,2245	3,2146	0,0563639	1613,68	3,2343	3,2247
**Spectrum 20**	UM	0	2	20/12/19	326,547	3,2245	3,2146	0,0647698	2205,2	3,2343	3,2247
**Spectrum 21**	UM	0	1	20/12/19	270,065	3,2245	3,2146	0,0537766	1848,64	3,2343	3,2247
**Spectrum 22**	UUM	0	3	27/12/19	182,562	3,2245	3,2146	0,0042729	1479,94	3,2343	3,2247
**Spectrum 23**	UUM	0	2	27/12/19	165,608	3,2245	3,2146	0,00480506	1370,31	3,2343	3,2247
**Spectrum 24**	UUM	0	1	27/12/19	216,405	3,2245	3,2146	0,00467109	1599,45	3,2343	3,2247
**Spectrum 25**	FM	0	3	4/02/20	45,0221	3,2245	3,2146	0,0397153	1616,75	3,2343	3,2247
**Spectrum 26**	FM	0	2	28/12/19	141,625	3,2245	3,2146	0,0266827	1287,32	3,2343	3,2247
**Spectrum 27**	FM	0	1	28/12/19	201,554	3,2245	3,2146	0,0480247	2197,23	3,2343	3,2247

	Method	Patient	Replicated	Date							
**Spectrum 1**	UUM	1	1	4/09/20	0,0339774	1965,41	3,2416	3,2342	0,0168948	977,277	3,8892
**Spectrum 2**	UUM	2	1	4/09/20	0,0181709	1885,47	3,2416	3,2342	0,00793336	823,193	3,8892
**Spectrum 3**	UUM	3	1	4/09/20	0,0220942	1753,77	3,2416	3,2342	0,00811842	644,415	3,8892
**Spectrum 4**	UUM	1	2	5/09/20	0,0374306	1874,25	3,2416	3,2342	0,0188998	946,363	3,8892
**Spectrum 5**	UUM	2	2	5/09/20	0,0420424	1946,21	3,2416	3,2342	0,0219003	1013,8	3,8892
**Spectrum 6**	UUM	3	2	5/09/20	0,0378086	2111,2	3,2416	3,2342	0,0144104	804,663	3,8892
**Spectrum 7**	UM	1	1	4/09/20	0,0777109	0,819066	3,2416	3,2342	0,0468988	0,494309	3,8892
**Spectrum 8**	UM	2	1	4/09/20	0,0803472	0,824436	3,2416	3,2342	0,0501821	0,514914	3,8892
**Spectrum 9**	UM	3	1	4/09/20	0,0837428	0,956902	3,2416	3,2342	0,0352163	0,402406	3,8892
**Spectrum 10**	UM	1	2	5/09/20	0,0844221	0,871508	3,2416	3,2342	0,047484	0,490188	3,8892
**Spectrum 11**	UM	2	2	5/09/20	0,0714425	0,905587	3,2416	3,2342	0,0412391	0,522736	3,8892
**Spectrum 12**	UM	3	2	5/09/20	0,0714435	0,893753	3,2416	3,2342	0,0343754	0,430034	3,8892
**Spectrum 13**	FM	1	2	17/09/20	0,0422067	1599,16	3,2416	3,2342	0,0151865	575,398	3,8892
**Spectrum 14**	FM	1	1	17/09/20	0,0504996	1685,87	3,2416	3,2342	0,0185957	620,796	3,8892
**Spectrum 15**	FM	2	1	17/09/20	0,0405488	1457,83	3,2416	3,2342	0,0160838	578,253	3,8892
**Spectrum 16**	FM	2	2	17/09/20	0,0425317	1584,1	3,2416	3,2342	0,0161826	602,723	3,8892
**Spectrum 17**	FM	3	1	17/09/20	0,0580238	1750,84	3,2416	3,2342	0,0137282	414,242	3,8892
**Spectrum 18**	FM	3	2	17/09/20	0,0516346	1569,95	3,2416	3,2342	0,0138698	421,712	3,8892
**Spectrum 19**	UM	0	3	20/12/19	0,065451	1873,84	3,2416	3,2342	0,0379243	1085,76	3,8892
**Spectrum 20**	UM	0	2	20/12/19	0,0769657	2620,43	3,2416	3,2342	0,0479488	1632,5	3,8892
**Spectrum 21**	UM	0	1	20/12/19	0,0625659	2150,79	3,2416	3,2342	0,0376579	1294,54	3,8892
**Spectrum 22**	UUM	0	3	27/12/19	0,00524863	1817,89	3,2416	3,2342	0,00310754	1076,31	3,8892
**Spectrum 23**	UUM	0	2	27/12/19	0,00645519	1840,9	3,2416	3,2342	0,00353905	1009,27	3,8892
**Spectrum 24**	UUM	0	1	27/12/19	0,00612277	2096,52	3,2416	3,2342	0,00346277	1185,7	3,8892
**Spectrum 25**	FM	0	3	4/02/20	0,0415214	1690,27	3,2416	3,2342	0,0155336	632,35	3,8892
**Spectrum 26**	FM	0	2	28/12/19	0,0346917	1673,71	3,2416	3,2342	0,0168689	813,843	3,8892
**Spectrum 27**	FM	0	1	28/12/19	0,0555632	2542,13	3,2416	3,2342	0,0270617	1238,13	3,8892

	Method	Patient	Replicated	Date							
**Spectrum 1**	UUM	1	1	4/09/20	3,8839	0,0038233	221,158	3,2803	3,2419	0,198789	11498,9
**Spectrum 2**	UUM	2	1	4/09/20	3,8839	0,00159029	165,014	3,2803	3,2419	0,0964126	10004,1
**Spectrum 3**	UUM	3	1	4/09/20	3,8839	0,00185596	147,321	3,2803	3,2419	0,181101	14375,2
**Spectrum 4**	UUM	1	2	5/09/20	3,8839	0,00109355	54,7571	3,2803	3,2419	0,237165	11875,5
**Spectrum 5**	UUM	2	2	5/09/20	3,8839	0,00219708	101,707	3,2803	3,2419	0,23722	10981,3
**Spectrum 6**	UUM	3	2	5/09/20	3,8839	0,00524166	292,69	3,2803	3,2419	0,284749	15900,2
**Spectrum 7**	UM	1	1	4/09/20	3,8839	0,0247539	0,260904	3,2803	3,2419	0,300525	3,16751
**Spectrum 8**	UM	2	1	4/09/20	3,8839	0,0254007	0,260635	3,2803	3,2419	0,270066	2,77112
**Spectrum 9**	UM	3	1	4/09/20	3,8839	0,0246894	0,282118	3,2803	3,2419	0,340369	3,88929
**Spectrum 10**	UM	1	2	5/09/20	3,8839	0,0258626	0,266986	3,2803	3,2419	0,303764	3,13582
**Spectrum 11**	UM	2	2	5/09/20	3,8839	0,0186991	0,237025	3,2803	3,2419	0,232066	2,94161
**Spectrum 12**	UM	3	2	5/09/20	3,8839	0,0191225	0,239221	3,2803	3,2419	0,328253	4,10642
**Spectrum 13**	FM	1	2	17/09/20	3,8839	0,00501013	189,827	3,2803	3,2419	0,163837	6207,57
**Spectrum 14**	FM	1	1	17/09/20	3,8839	0,00667525	222,845	3,2803	3,2419	0,189639	6330,86
**Spectrum 15**	FM	2	1	17/09/20	3,8839	0,00210015	75,5057	3,2803	3,2419	0,137047	4927,2
**Spectrum 16**	FM	2	2	17/09/20	3,8839	0,00543304	202,354	3,2803	3,2419	0,145169	5406,83
**Spectrum 17**	FM	3	1	17/09/20	3,8839	0,00485382	146,461	3,2803	3,2419	0,325913	9834,25
**Spectrum 18**	FM	3	2	17/09/20	3,8839	0,00549737	167,148	3,2803	3,2419	0,275182	8366,91
**Spectrum 19**	UM	0	3	20/12/19	3,8839	0,0136967	392,132	3,2803	3,2419	0,282535	8088,88
**Spectrum 20**	UM	0	2	20/12/19	3,8839	0,0176219	599,966	3,2803	3,2419	0,347978	11847,5
**Spectrum 21**	UM	0	1	20/12/19	3,8839	0,0139556	479,742	3,2803	3,2419	0,278815	9584,64
**Spectrum 22**	UUM	0	3	27/12/19	3,8839	0,00014022	48,5658	3,2803	3,2419	0,0300014	10391,1
**Spectrum 23**	UUM	0	2	27/12/19	3,8839	0,00021705	61,8974	3,2803	3,2419	0,0321041	9155,49
**Spectrum 24**	UUM	0	1	27/12/19	3,8839	0,0001803	61,7365	3,2803	3,2419	0,030634	10489,5
**Spectrum 25**	FM	0	3	4/02/20	3,8839	0,00497071	202,35	3,2803	3,2419	0,201718	8211,65
**Spectrum 26**	FM	0	2	28/12/19	3,8839	0,00470839	227,158	3,2803	3,2419	0,138804	6696,63
**Spectrum 27**	FM	0	1	28/12/19	3,8839	0,0115986	530,659	3,2803	3,2419	0,244027	11164,8

	Method	Patient	Replicated	Date							
**Spectrum 1**	UUM	1	1	4/09/20	7,4547	7,4125	0,00798308	461,779	7,4036	7,3753	0,0045477
**Spectrum 2**	UUM	2	1	4/09/20	7,4547	7,4125	0,00369013	382,901	7,4036	7,3753	0,0014752
**Spectrum 3**	UUM	3	1	4/09/20	7,4547	7,4125	0,00569089	451,725	7,4036	7,3753	0,0032399
**Spectrum 4**	UUM	1	2	5/09/20	7,4547	7,4125	0,00778558	389,845	7,4036	7,3753	0,0051074
**Spectrum 5**	UUM	2	2	5/09/20	7,4547	7,4125	0,00933601	432,18	7,4036	7,3753	0,00307934
**Spectrum 6**	UUM	3	2	5/09/20	7,4547	7,4125	0,00965413	539,078	7,4036	7,3753	0,00302403
**Spectrum 7**	UM	1	1	4/09/20	7,4547	7,4125	0,0348511	0,367327	7,4036	7,3753	0,0221564
**Spectrum 8**	UM	2	1	4/09/20	7,4547	7,4125	0,0360973	0,370392	7,4036	7,3753	0,0246151
**Spectrum 9**	UM	3	1	4/09/20	7,4547	7,4125	0,0295393	0,337537	7,4036	7,3753	0,0185411
**Spectrum 10**	UM	1	2	5/09/20	7,4547	7,4125	0,0334422	0,345231	7,4036	7,3753	0,0236818
**Spectrum 11**	UM	2	2	5/09/20	7,4547	7,4125	0,0240832	0,305272	7,4036	7,3753	0,0165839
**Spectrum 12**	UM	3	2	5/09/20	7,4547	7,4125	0,00580302	0,0725954	7,4036	7,3753	0,00673702
**Spectrum 13**	FM	1	2	17/09/20	7,4547	7,4125	-0,0054795	-207,612	7,4036	7,3753	5,84E-06
**Spectrum 14**	FM	1	1	17/09/20	7,4547	7,4125	-0,0114663	-382,787	7,4036	7,3753	-0,0039273
**Spectrum 15**	FM	2	1	17/09/20	7,4547	7,4125	0,00054925	19,747	7,4036	7,3753	0,00448707
**Spectrum 16**	FM	2	2	17/09/20	7,4547	7,4125	-0,0100559	-374,534	7,4036	7,3753	-0,0058943
**Spectrum 17**	FM	3	1	17/09/20	7,4547	7,4125	-0,0019711	-59,4768	7,4036	7,3753	-0,0040785
**Spectrum 18**	FM	3	2	17/09/20	7,4547	7,4125	-0,0075368	-229,155	7,4036	7,3753	-0,0020779
**Spectrum 19**	UM	0	3	20/12/19	7,4547	7,4125	0,0251548	720,173	7,4036	7,3753	0,0188515
**Spectrum 20**	UM	0	2	20/12/19	7,4547	7,4125	0,0397822	1354,45	7,4036	7,3753	0,0262358
**Spectrum 21**	UM	0	1	20/12/19	7,4547	7,4125	0,0259541	892,206	7,4036	7,3753	0,0168345
**Spectrum 22**	UUM	0	3	27/12/19	7,4547	7,4125	0,00268089	928,539	7,4036	7,3753	0,00123363
**Spectrum 23**	UUM	0	2	27/12/19	7,4547	7,4125	0,00252845	721,064	7,4036	7,3753	0,00113681
**Spectrum 24**	UUM	0	1	27/12/19	7,4547	7,4125	0,00202334	692,819	7,4036	7,3753	0,00088093
**Spectrum 25**	FM	0	3	4/02/20	7,4547	7,4125	0,00273397	111,296	7,4036	7,3753	0,00036054
**Spectrum 26**	FM	0	2	28/12/19	7,4547	7,4125	0,00693986	334,816	7,4036	7,3753	0,00458
**Spectrum 27**	FM	0	1	28/12/19	7,4547	7,4125	0,00768421	351,569	7,4036	7,3753	0,00672995

	Method	Patient	Replicated	Date							
**Spectrum 1**	UUM	1	1	4/09/20	263,06	7,3525	7,2973	0,0110174	637,301	3,377	3,3531
**Spectrum 2**	UUM	2	1	4/09/20	153,071	7,3525	7,2973	0,00446891	463,709	3,377	3,3531
**Spectrum 3**	UUM	3	1	4/09/20	257,173	7,3525	7,2973	0,00778955	618,31	3,377	3,3531
**Spectrum 4**	UUM	1	2	5/09/20	255,741	7,3525	7,2973	0,0134882	675,39	3,377	3,3531
**Spectrum 5**	UUM	2	2	5/09/20	142,548	7,3525	7,2973	0,0119393	552,692	3,377	3,3531
**Spectrum 6**	UUM	3	2	5/09/20	168,859	7,3525	7,2973	0,0129335	722,198	3,377	3,3531
**Spectrum 7**	UM	1	1	4/09/20	0,233526	7,3525	7,2973	0,0807474	0,851072	3,377	3,3531
**Spectrum 8**	UM	2	1	4/09/20	0,252573	7,3525	7,2973	0,0778574	0,798889	3,377	3,3531
**Spectrum 9**	UM	3	1	4/09/20	0,211863	7,3525	7,2973	0,0628228	0,717856	3,377	3,3531
**Spectrum 10**	UM	1	2	5/09/20	0,244472	7,3525	7,2973	0,0808541	0,834675	3,377	3,3531
**Spectrum 11**	UM	2	2	5/09/20	0,210214	7,3525	7,2973	0,058266	0,738566	3,377	3,3531
**Spectrum 12**	UM	3	2	5/09/20	0,0842796	7,3525	7,2973	0,0452047	0,565507	3,377	3,3531
**Spectrum 13**	FM	1	2	17/09/20	0,221137	7,3525	7,2973	0,00249152	94,4003	3,377	3,3531
**Spectrum 14**	FM	1	1	17/09/20	-131,107	7,3525	7,2973	-0,0021436	-71,5625	3,377	3,3531
**Spectrum 15**	FM	2	1	17/09/20	161,322	7,3525	7,2973	0,00897283	322,596	3,377	3,3531
**Spectrum 16**	FM	2	2	17/09/20	-219,533	7,3525	7,2973	-0,0040237	-149,861	3,377	3,3531
**Spectrum 17**	FM	3	1	17/09/20	-123,066	7,3525	7,2973	0,00327574	98,8437	3,377	3,3531
**Spectrum 18**	FM	3	2	17/09/20	-63,1779	7,3525	7,2973	-0,0020772	-63,1569	3,377	3,3531
**Spectrum 19**	UM	0	3	20/12/19	539,714	7,3525	7,2973	0,116955	3348,37	3,377	3,3531
**Spectrum 20**	UM	0	2	20/12/19	893,242	7,3525	7,2973	0,12536	4268,1	3,377	3,3531
**Spectrum 21**	UM	0	1	20/12/19	578,707	7,3525	7,2973	0,100763	3463,85	3,377	3,3531
**Spectrum 22**	UUM	0	3	27/12/19	427,274	7,3525	7,2973	0,00537884	1862,99	3,377	3,3531
**Spectrum 23**	UUM	0	2	27/12/19	324,195	7,3525	7,2973	0,00523447	1492,77	3,377	3,3531
**Spectrum 24**	UUM	0	1	27/12/19	301,644	7,3525	7,2973	0,00442653	1515,71	3,377	3,3531
**Spectrum 25**	FM	0	3	4/02/20	14,677	7,3525	7,2973	0,0238178	969,587	3,377	3,3531
**Spectrum 26**	FM	0	2	28/12/19	220,964	7,3525	7,2973	0,0321991	1553,46	3,377	3,3531
**Spectrum 27**	FM	0	1	28/12/19	307,909	7,3525	7,2973	0,0415787	1902,31	3,377	3,3531

	Method	Patient	Replicated	Date							
**Spectrum 1**	UUM	1	1	4/09/20	0,0894237	5172,68	3,818	3,7665	1,07184	62000,5	3,5415
**Spectrum 2**	UUM	2	1	4/09/20	0,0487975	5063,4	3,818	3,7665	0,808716	83915,1	3,5415
**Spectrum 3**	UUM	3	1	4/09/20	0,0665192	5280,09	3,818	3,7665	1,07255	85135,9	3,5415
**Spectrum 4**	UUM	1	2	5/09/20	0,235511	11792,7	3,818	3,7665	0,735764	36841,7	3,5415
**Spectrum 5**	UUM	2	2	5/09/20	0,282934	13097,5	3,818	3,7665	0,620836	28739,6	3,5415
**Spectrum 6**	UUM	3	2	5/09/20	0,211854	11829,8	3,818	3,7665	0,825403	46089,8	3,5415
**Spectrum 7**	UM	1	1	4/09/20	0,050817	0,535607	3,818	3,7665	0,28498	3,00366	3,5415
**Spectrum 8**	UM	2	1	4/09/20	0,0537201	0,551217	3,818	3,7665	0,279944	2,87248	3,5415
**Spectrum 9**	UM	3	1	4/09/20	0,0488555	0,558257	3,818	3,7665	0,296276	3,38546	3,5415
**Spectrum 10**	UM	1	2	5/09/20	0,0424592	0,438315	3,818	3,7665	0,279357	2,88387	3,5415
**Spectrum 11**	UM	2	2	5/09/20	0,0465859	0,590512	3,818	3,7665	0,222046	2,81459	3,5415
**Spectrum 12**	UM	3	2	5/09/20	0,0370751	0,463806	3,818	3,7665	0,232421	2,90757	3,5415
**Spectrum 13**	FM	1	2	17/09/20	0,0253245	959,512	3,818	3,7665	0,0818202	3100,06	3,5415
**Spectrum 14**	FM	1	1	17/09/20	0,0442448	1477,06	3,818	3,7665	0,102987	3438,11	3,5415
**Spectrum 15**	FM	2	1	17/09/20	0,090517	3254,32	3,818	3,7665	0,0769664	2767,14	3,5415
**Spectrum 16**	FM	2	2	17/09/20	0,0379327	1412,81	3,818	3,7665	0,0825345	3074,01	3,5415
**Spectrum 17**	FM	3	1	17/09/20	0,0351577	1060,86	3,818	3,7665	0,148686	4486,51	3,5415
**Spectrum 18**	FM	3	2	17/09/20	0,0633892	1927,35	3,818	3,7665	0,137608	4183,97	3,5415
**Spectrum 19**	UM	0	3	20/12/19	0,115157	3296,9	3,818	3,7665	0,144476	4136,29	3,5415
**Spectrum 20**	UM	0	2	20/12/19	0,0346597	1180,05	3,818	3,7665	0,130319	4436,94	3,5415
**Spectrum 21**	UM	0	1	20/12/19	0,274922	9450,82	3,818	3,7665	0,100898	3468,5	3,5415
**Spectrum 22**	UUM	0	3	27/12/19	0,00355512	1231,33	3,818	3,7665	0,0314466	10891,7	3,5415
**Spectrum 23**	UUM	0	2	27/12/19	0,00357128	1018,46	3,818	3,7665	0,026286	7496,26	3,5415
**Spectrum 24**	UUM	0	1	27/12/19	0,00378667	1296,61	3,818	3,7665	0,0175824	6020,47	3,5415
**Spectrum 25**	FM	0	3	4/02/20	0,0479266	1951,02	3,818	3,7665	0,0414886	1688,94	3,5415
**Spectrum 26**	FM	0	2	28/12/19	0,0128838	621,584	3,818	3,7665	0,0468844	2261,95	3,5415
**Spectrum 27**	FM	0	1	28/12/19	0,0162269	742,412	3,818	3,7665	0,0750282	3432,69	3,5415

	Method	Patient	Replicated	Date							
**Spectrum 1**	UUM	1	1	4/09/20	3,5165	0,0316829	1832,69	4,2436	4,1836	0,0137221	793,753
**Spectrum 2**	UUM	2	1	4/09/20	3,5165	0,015581	1616,74	4,2436	4,1836	0,00870759	903,529
**Spectrum 3**	UUM	3	1	4/09/20	3,5165	0,0260352	2066,59	4,2436	4,1836	0,00145278	115,317
**Spectrum 4**	UUM	1	2	5/09/20	3,5165	0,0229411	1148,72	4,2436	4,1836	-0,0028505	-142,732
**Spectrum 5**	UUM	2	2	5/09/20	3,5165	0,0221617	1025,9	4,2436	4,1836	0,00978127	452,791
**Spectrum 6**	UUM	3	2	5/09/20	3,5165	0,0297116	1659,07	4,2436	4,1836	0,0159337	889,723
**Spectrum 7**	UM	1	1	4/09/20	3,5165	0,0154241	0,162569	4,2436	4,1836	0,159869	1,685
**Spectrum 8**	UM	2	1	4/09/20	3,5165	0,022025	0,225997	4,2436	4,1836	0,175446	1,80024
**Spectrum 9**	UM	3	1	4/09/20	3,5165	0,0248763	0,284254	4,2436	4,1836	0,139088	1,58931
**Spectrum 10**	UM	1	2	5/09/20	3,5165	0,0156166	0,161213	4,2436	4,1836	0,152862	1,57802
**Spectrum 11**	UM	2	2	5/09/20	3,5165	0,0171058	0,216829	4,2436	4,1836	0,133202	1,68843
**Spectrum 12**	UM	3	2	5/09/20	3,5165	0,0149508	0,187033	4,2436	4,1836	0,0612694	0,766476
**Spectrum 13**	FM	1	2	17/09/20	3,5165	0,00730306	276,703	4,2436	4,1836	0,0230051	871,634
**Spectrum 14**	FM	1	1	17/09/20	3,5165	0,0117736	393,048	4,2436	4,1836	0,054243	1810,84
**Spectrum 15**	FM	2	1	17/09/20	3,5165	0,00261753	94,1071	4,2436	4,1836	0,00792789	285,028
**Spectrum 16**	FM	2	2	17/09/20	3,5165	0,0110644	412,094	4,2436	4,1836	0,0343553	1279,57
**Spectrum 17**	FM	3	1	17/09/20	3,5165	0,010039	302,921	4,2436	4,1836	0,0296567	894,874
**Spectrum 18**	FM	3	2	17/09/20	3,5165	0,0135146	410,912	4,2436	4,1836	0,0495269	1505,87
**Spectrum 19**	UM	0	3	20/12/19	3,5165	0,0185764	531,836	4,2436	4,1836	0,0195335	559,237
**Spectrum 20**	UM	0	2	20/12/19	3,5165	0,0188236	640,883	4,2436	4,1836	0,00936041	318,691
**Spectrum 21**	UM	0	1	20/12/19	3,5165	0,0129816	446,261	4,2436	4,1836	0,00489382	168,232
**Spectrum 22**	UUM	0	3	27/12/19	3,5165	0,00919812	3185,81	4,2436	4,1836	-0,0002964	-102,647
**Spectrum 23**	UUM	0	2	27/12/19	3,5165	0,00890227	2538,76	4,2436	4,1836	-0,0008579	-244,66
**Spectrum 24**	UUM	0	1	27/12/19	3,5165	0,0085662	2933,19	4,2436	4,1836	-0,0004885	-167,28
**Spectrum 25**	FM	0	3	4/02/20	3,5165	0,0119386	486,004	4,2436	4,1836	-0,0014565	-59,291
**Spectrum 26**	FM	0	2	28/12/19	3,5165	0,00779666	376,152	4,2436	4,1836	-0,0084371	-407,052
**Spectrum 27**	FM	0	1	28/12/19	3,5165	0,0159843	731,314	4,2436	4,1836	0,00283194	129,567

	Method	Patient	Replicated	Date							
**Spectrum 1**	UUM	1	1	4/09/20	3,6347	3,6244	0,0183845	1063,44	3,9759	3,9389	0,0300154
**Spectrum 2**	UUM	2	1	4/09/20	3,6347	3,6244	0,0108192	1122,64	3,9759	3,9389	0,0143086
**Spectrum 3**	UUM	3	1	4/09/20	3,6347	3,6244	0,0160387	1273,1	3,9759	3,9389	0,0184756
**Spectrum 4**	UUM	1	2	5/09/20	3,6347	3,6244	0,0145639	729,255	3,9759	3,9389	0,031271
**Spectrum 5**	UUM	2	2	5/09/20	3,6347	3,6244	0,0157474	728,973	3,9759	3,9389	0,0304311
**Spectrum 6**	UUM	3	2	5/09/20	3,6347	3,6244	0,0204578	1142,34	3,9759	3,9389	0,0226003
**Spectrum 7**	UM	1	1	4/09/20	3,6347	3,6244	0,0172731	0,182057	3,9759	3,9389	0,162845
**Spectrum 8**	UM	2	1	4/09/20	3,6347	3,6244	0,0230669	0,236688	3,9759	3,9389	0,172296
**Spectrum 9**	UM	3	1	4/09/20	3,6347	3,6244	0,0237931	0,271876	3,9759	3,9389	0,137215
**Spectrum 10**	UM	1	2	5/09/20	3,6347	3,6244	0,0193043	0,199283	3,9759	3,9389	0,161981
**Spectrum 11**	UM	2	2	5/09/20	3,6347	3,6244	0,0196902	0,249588	3,9759	3,9389	0,132228
**Spectrum 12**	UM	3	2	5/09/20	3,6347	3,6244	0,016079	0,201147	3,9759	3,9389	0,114672
**Spectrum 13**	FM	1	2	17/09/20	3,6347	3,6244	0,00588555	222,996	3,9759	3,9389	0,0342759
**Spectrum 14**	FM	1	1	17/09/20	3,6347	3,6244	0,00408873	136,497	3,9759	3,9389	0,0401797
**Spectrum 15**	FM	2	1	17/09/20	3,6347	3,6244	0,00462394	166,242	3,9759	3,9389	0,0154207
**Spectrum 16**	FM	2	2	17/09/20	3,6347	3,6244	0,00572795	213,338	3,9759	3,9389	0,0301001
**Spectrum 17**	FM	3	1	17/09/20	3,6347	3,6244	0,00600228	181,115	3,9759	3,9389	0,0160655
**Spectrum 18**	FM	3	2	17/09/20	3,6347	3,6244	0,00354576	107,809	3,9759	3,9389	0,0249957
**Spectrum 19**	UM	0	3	20/12/19	3,6347	3,6244	0,0126302	361,598	3,9759	3,9389	0,0793752
**Spectrum 20**	UM	0	2	20/12/19	3,6347	3,6244	0,0159848	544,231	3,9759	3,9389	0,0801639
**Spectrum 21**	UM	0	1	20/12/19	3,6347	3,6244	0,0130868	449,876	3,9759	3,9389	0,0595331
**Spectrum 22**	UUM	0	3	27/12/19	3,6347	3,6244	0,0002616	90,6072	3,9759	3,9389	0,00294159
**Spectrum 23**	UUM	0	2	27/12/19	3,6347	3,6244	-0,0007849	-223,833	3,9759	3,9389	0,00368129
**Spectrum 24**	UUM	0	1	27/12/19	3,6347	3,6244	0,00045957	157,365	3,9759	3,9389	0,00317813
**Spectrum 25**	FM	0	3	4/02/20	3,6347	3,6244	0,00986014	401,392	3,9759	3,9389	0,0159033
**Spectrum 26**	FM	0	2	28/12/19	3,6347	3,6244	0,00662021	319,394	3,9759	3,9389	0,0193496
**Spectrum 27**	FM	0	1	28/12/19	3,6347	3,6244	0,0148686	680,267	3,9759	3,9389	0,0329461

	Method	Patient	Replicated	Date								
**Spectrum 1**	UUM	1	1	4/09/20	1736,23	7,2147	7,1804	0,0076698	443,657	6,9311	6,8779
**Spectrum 2**	UUM	2	1	4/09/20	1484,71	7,2147	7,1804	0,00400199	415,26	6,9311	6,8779
**Spectrum 3**	UUM	3	1	4/09/20	1466,53	7,2147	7,1804	0,00733126	581,932	6,9311	6,8779
**Spectrum 4**	UUM	1	2	5/09/20	1565,82	7,2147	7,1804	0,01182	591,859	6,9311	6,8779
**Spectrum 5**	UUM	2	2	5/09/20	1408,71	7,2147	7,1804	0,0115417	534,286	6,9311	6,8779
**Spectrum 6**	UUM	3	2	5/09/20	1261,98	7,2147	7,1804	0,00819373	457,531	6,9311	6,8779
**Spectrum 7**	UM	1	1	4/09/20	1,71638	7,2147	7,1804	0,0443174	0,467102	6,9311	6,8779
**Spectrum 8**	UM	2	1	4/09/20	1,76791	7,2147	7,1804	0,0455419	0,467301	6,9311	6,8779
**Spectrum 9**	UM	3	1	4/09/20	1,56791	7,2147	7,1804	0,0359275	0,410532	6,9311	6,8779
**Spectrum 10**	UM	1	2	5/09/20	1,67216	7,2147	7,1804	0,0445649	0,460053	6,9311	6,8779
**Spectrum 11**	UM	2	2	5/09/20	1,67609	7,2147	7,1804	0,0339968	0,430935	6,9311	6,8779
**Spectrum 12**	UM	3	2	5/09/20	1,43454	7,2147	7,1804	0,0187186	0,234169	6,9311	6,8779
**Spectrum 13**	FM	1	2	17/09/20	1298,67	7,2147	7,1804	-0,0021452	-81,2801	6,9311	6,8779
**Spectrum 14**	FM	1	1	17/09/20	1341,35	7,2147	7,1804	-0,0090332	-301,562	6,9311	6,8779
**Spectrum 15**	FM	2	1	17/09/20	554,412	7,2147	7,1804	0,00075541	27,159	6,9311	6,8779
**Spectrum 16**	FM	2	2	17/09/20	1121,08	7,2147	7,1804	-0,0066612	-248,097	6,9311	6,8779
**Spectrum 17**	FM	3	1	17/09/20	484,768	7,2147	7,1804	-0,0036454	-109,999	6,9311	6,8779
**Spectrum 18**	FM	3	2	17/09/20	759,996	7,2147	7,1804	-0,0018557	-56,4228	6,9311	6,8779
**Spectrum 19**	UM	0	3	20/12/19	2272,49	7,2147	7,1804	0,0473243	1354,88	6,9311	6,8779
**Spectrum 20**	UM	0	2	20/12/19	2729,32	7,2147	7,1804	0,0643058	2189,4	6,9311	6,8779
**Spectrum 21**	UM	0	1	20/12/19	2046,53	7,2147	7,1804	0,0485653	1669,5	6,9311	6,8779
**Spectrum 22**	UUM	0	3	27/12/19	1018,83	7,2147	7,1804	0,00317781	1100,65	6,9311	6,8779
**Spectrum 23**	UUM	0	2	27/12/19	1049,83	7,2147	7,1804	0,0033643	959,433	6,9311	6,8779
**Spectrum 24**	UUM	0	1	27/12/19	1088,24	7,2147	7,1804	0,00310792	1064,2	6,9311	6,8779
**Spectrum 25**	FM	0	3	4/02/20	647,399	7,2147	7,1804	0,00140589	57,2318	6,9311	6,8779
**Spectrum 26**	FM	0	2	28/12/19	933,526	7,2147	7,1804	0,00487246	235,073	6,9311	6,8779
**Spectrum 27**	FM	0	1	28/12/19	1507,35	7,2147	7,1804	0,00507003	231,964	6,9311	6,8779

	Method	Patient	Replicated	Date							
**Spectrum 1**	UUM	1	1	4/09/20	0,0114	659,427	4,0051	3,9761	0,0570021	3297,27	7,1227
**Spectrum 2**	UUM	2	1	4/09/20	0,00454342	471,441	4,0051	3,9761	0,0296167	3073,13	7,1227
**Spectrum 3**	UUM	3	1	4/09/20	0,0106718	847,092	4,0051	3,9761	0,0303644	2410,23	7,1227
**Spectrum 4**	UUM	1	2	5/09/20	0,0249125	1247,44	4,0051	3,9761	0,0593983	2974,24	7,1227
**Spectrum 5**	UUM	2	2	5/09/20	0,0143344	663,564	4,0051	3,9761	0,0702274	3250,95	7,1227
**Spectrum 6**	UUM	3	2	5/09/20	0,0129395	722,53	4,0051	3,9761	0,0399296	2229,64	7,1227
**Spectrum 7**	UM	1	1	4/09/20	0,0529044	0,557608	4,0051	3,9761	0,178529	1,88168	7,1227
**Spectrum 8**	UM	2	1	4/09/20	0,0523799	0,537466	4,0051	3,9761	0,19344	1,98487	7,1227
**Spectrum 9**	UM	3	1	4/09/20	0,0422652	0,482951	4,0051	3,9761	0,147095	1,68081	7,1227
**Spectrum 10**	UM	1	2	5/09/20	0,0533964	0,551223	4,0051	3,9761	0,175127	1,80787	7,1227
**Spectrum 11**	UM	2	2	5/09/20	0,0379466	0,481002	4,0051	3,9761	0,148834	1,88658	7,1227
**Spectrum 12**	UM	3	2	5/09/20	0,024045	0,300802	4,0051	3,9761	0,101308	1,26735	7,1227
**Spectrum 13**	FM	1	2	17/09/20	-0,0057521	-217,938	4,0051	3,9761	0,0602935	2284,44	7,1227
**Spectrum 14**	FM	1	1	17/09/20	-0,0170936	-570,65	4,0051	3,9761	0,0772527	2578,99	7,1227
**Spectrum 15**	FM	2	1	17/09/20	-0,0021659	-77,8679	4,0051	3,9761	0,0552958	1988,03	7,1227
**Spectrum 16**	FM	2	2	17/09/20	-0,0109489	-407,795	4,0051	3,9761	0,0674942	2513,83	7,1227
**Spectrum 17**	FM	3	1	17/09/20	-0,0060854	-183,623	4,0051	3,9761	0,0474836	1432,79	7,1227
**Spectrum 18**	FM	3	2	17/09/20	-0,0073693	-224,063	4,0051	3,9761	0,055345	1682,77	7,1227
**Spectrum 19**	UM	0	3	20/12/19	0,0303762	869,66	4,0051	3,9761	0,0738737	2114,98	7,1227
**Spectrum 20**	UM	0	2	20/12/19	0,0440304	1499,09	4,0051	3,9761	0,0563604	1918,89	7,1227
**Spectrum 21**	UM	0	1	20/12/19	0,0318615	1095,28	4,0051	3,9761	0,0432643	1487,27	7,1227
**Spectrum 22**	UUM	0	3	27/12/19	0,00270663	937,454	4,0051	3,9761	0,00459971	1593,13	7,1227
**Spectrum 23**	UUM	0	2	27/12/19	0,00258581	737,424	4,0051	3,9761	0,00561886	1602,39	7,1227
**Spectrum 24**	UUM	0	1	27/12/19	0,00213811	732,121	4,0051	3,9761	0,00467555	1600,98	7,1227
**Spectrum 25**	FM	0	3	4/02/20	-0,0011101	-45,1899	4,0051	3,9761	0,0185719	756,034	7,1227
**Spectrum 26**	FM	0	2	28/12/19	0,00231484	111,68	4,0051	3,9761	0,0225969	1090,19	7,1227
**Spectrum 27**	FM	0	1	28/12/19	0,00180647	82,6497	4,0051	3,9761	0,029062	1329,65	7,1227

	Method	Patient	Replicated	Date								
**Spectrum 1**	UUM	1	1	4/09/20	7,0595	0,00271017	156,769	7,5018	7,4604	0,00203672	117,813
**Spectrum 2**	UUM	2	1	4/09/20	7,0595	0,00105203	109,162	7,5018	7,4604	0,00081912	84,9947
**Spectrum 3**	UUM	3	1	4/09/20	7,0595	0,00375807	298,303	7,5018	7,4604	0,00234994	186,531
**Spectrum 4**	UUM	1	2	5/09/20	7,0595	0,00895877	448,59	7,5018	7,4604	-0,0001174	-5,87663
**Spectrum 5**	UUM	2	2	5/09/20	7,0595	0,00139927	64,7748	7,5018	7,4604	0,0032796	151,818
**Spectrum 6**	UUM	3	2	5/09/20	7,0595	0,0072172	403,002	7,5018	7,4604	0,0046919	261,992
**Spectrum 7**	UM	1	1	4/09/20	7,0595	0,0492134	0,518706	7,5018	7,4604	0,0145853	0,153728
**Spectrum 8**	UM	2	1	4/09/20	7,0595	0,0467565	0,479764	7,5018	7,4604	0,0141824	0,145524
**Spectrum 9**	UM	3	1	4/09/20	7,0595	0,0413237	0,472192	7,5018	7,4604	0,00914692	0,104519
**Spectrum 10**	UM	1	2	5/09/20	7,0595	0,057404	0,592594	7,5018	7,4604	0,0129393	0,133575
**Spectrum 11**	UM	2	2	5/09/20	7,0595	0,0384111	0,48689	7,5018	7,4604	0,0108944	0,138095
**Spectrum 12**	UM	3	2	5/09/20	7,0595	0,0307566	0,384763	7,5018	7,4604	0,00038502	0,00481661
**Spectrum 13**	FM	1	2	17/09/20	7,0595	-0,0054101	-204,983	7,5018	7,4604	-0,0029269	-110,898
**Spectrum 14**	FM	1	1	17/09/20	7,0595	-0,0118062	-394,134	7,5018	7,4604	-0,0094601	-315,815
**Spectrum 15**	FM	2	1	17/09/20	7,0595	-4,84E-05	-1,73975	7,5018	7,4604	0,00019749	7,10025
**Spectrum 16**	FM	2	2	17/09/20	7,0595	-0,0127125	-473,477	7,5018	7,4604	-0,0080876	-301,223
**Spectrum 17**	FM	3	1	17/09/20	7,0595	0,00080936	24,4221	7,5018	7,4604	-0,0047129	-142,209
**Spectrum 18**	FM	3	2	17/09/20	7,0595	-0,010269	-312,23	7,5018	7,4604	-0,0047746	-145,171
**Spectrum 19**	UM	0	3	20/12/19	7,0595	0,0209493	599,772	7,5018	7,4604	0,0272044	778,853
**Spectrum 20**	UM	0	2	20/12/19	7,0595	0,0256844	874,468	7,5018	7,4604	0,0276588	941,693
**Spectrum 21**	UM	0	1	20/12/19	7,0595	0,0218636	751,591	7,5018	7,4604	0,0232253	798,4
**Spectrum 22**	UUM	0	3	27/12/19	7,0595	0,00107216	371,346	7,5018	7,4604	0,00210599	729,421
**Spectrum 23**	UUM	0	2	27/12/19	7,0595	0,00080764	230,325	7,5018	7,4604	0,00153011	436,357
**Spectrum 24**	UUM	0	1	27/12/19	7,0595	0,00034563	118,347	7,5018	7,4604	0,00129985	445,087
**Spectrum 25**	FM	0	3	4/02/20	7,0595	-0,0015444	-62,8719	7,5018	7,4604	0,0137086	558,057
**Spectrum 26**	FM	0	2	28/12/19	7,0595	0,00398781	192,393	7,5018	7,4604	0,0145095	700,014
**Spectrum 27**	FM	0	1	28/12/19	7,0595	0,00303618	138,911	7,5018	7,4604	0,00864717	395,626

	Method	Patient	Replicated	Date							
**Spectrum 1**	UUM	1	1	4/09/20	7,8583	7,8134	0,00426948	246,967	8,0456	8,0185	0,00264604
**Spectrum 2**	UUM	2	1	4/09/20	7,8583	7,8134	0,00072227	74,945	8,0456	8,0185	-1,34E-05
**Spectrum 3**	UUM	3	1	4/09/20	7,8583	7,8134	0,00174824	138,77	8,0456	8,0185	0,00132214
**Spectrum 4**	UUM	1	2	5/09/20	7,8583	7,8134	0,00366288	183,411	8,0456	8,0185	0,00309476
**Spectrum 5**	UUM	2	2	5/09/20	7,8583	7,8134	0,00236495	109,478	8,0456	8,0185	0,00077545
**Spectrum 6**	UUM	3	2	5/09/20	7,8583	7,8134	0,0025321	141,39	8,0456	8,0185	0,00262535
**Spectrum 7**	UM	1	1	4/09/20	7,8583	7,8134	0,0314849	0,331849	8,0456	8,0185	0,0233715
**Spectrum 8**	UM	2	1	4/09/20	7,8583	7,8134	0,0340803	0,349695	8,0456	8,0185	0,020152
**Spectrum 9**	UM	3	1	4/09/20	7,8583	7,8134	0,0263466	0,301054	8,0456	8,0185	0,0196827
**Spectrum 10**	UM	1	2	5/09/20	7,8583	7,8134	0,0338835	0,349787	8,0456	8,0185	0,0224521
**Spectrum 11**	UM	2	2	5/09/20	7,8583	7,8134	0,0273916	0,347209	8,0456	8,0185	0,015975
**Spectrum 12**	UM	3	2	5/09/20	7,8583	7,8134	0,0149361	0,18685	8,0456	8,0185	0,0132319
**Spectrum 13**	FM	1	2	17/09/20	7,8583	7,8134	-0,0028395	-107,586	8,0456	8,0185	-0,0023545
**Spectrum 14**	FM	1	1	17/09/20	7,8583	7,8134	-0,0106973	-357,117	8,0456	8,0185	-0,0071026
**Spectrum 15**	FM	2	1	17/09/20	7,8583	7,8134	-0,0022268	-80,0595	8,0456	8,0185	-0,0015556
**Spectrum 16**	FM	2	2	17/09/20	7,8583	7,8134	-0,0079315	-295,411	8,0456	8,0185	-0,005188
**Spectrum 17**	FM	3	1	17/09/20	7,8583	7,8134	-0,005518	-166,501	8,0456	8,0185	-0,001434
**Spectrum 18**	FM	3	2	17/09/20	7,8583	7,8134	-0,0088398	-268,774	8,0456	8,0185	-0,0038202
**Spectrum 19**	UM	0	3	20/12/19	7,8583	7,8134	0,0318515	911,898	8,0456	8,0185	0,0286305
**Spectrum 20**	UM	0	2	20/12/19	7,8583	7,8134	0,0368441	1254,42	8,0456	8,0185	0,0310933
**Spectrum 21**	UM	0	1	20/12/19	7,8583	7,8134	0,0293756	1009,82	8,0456	8,0185	0,0247379
**Spectrum 22**	UUM	0	3	27/12/19	7,8583	7,8134	0,00148024	512,689	8,0456	8,0185	0,00085426
**Spectrum 23**	UUM	0	2	27/12/19	7,8583	7,8134	0,00092501	263,795	8,0456	8,0185	0,00081998
**Spectrum 24**	UUM	0	1	27/12/19	7,8583	7,8134	0,00098622	337,697	8,0456	8,0185	0,00064303
**Spectrum 25**	FM	0	3	4/02/20	7,8583	7,8134	0,00461883	188,026	8,0456	8,0185	0,0004478
**Spectrum 26**	FM	0	2	28/12/19	7,8583	7,8134	0,00916032	441,942	8,0456	8,0185	0,00519325
**Spectrum 27**	FM	0	1	28/12/19	7,8583	7,8134	0,0125544	574,391	8,0456	8,0185	0,00748104

	Method	Patient	Replicated	Date							
**Spectrum 1**	UUM	1	1	4/09/20	153,059	6,1597	6,1248	0,00173818	100,545	8,2025	8,1871
**Spectrum 2**	UUM	2	1	4/09/20	-1,39207	6,1597	6,1248	0,00103565	107,463	8,2025	8,1871
**Spectrum 3**	UUM	3	1	4/09/20	104,947	6,1597	6,1248	0,00349871	277,716	8,2025	8,1871
**Spectrum 4**	UUM	1	2	5/09/20	154,963	6,1597	6,1248	-0,0035336	-176,937	8,2025	8,1871
**Spectrum 5**	UUM	2	2	5/09/20	35,8971	6,1597	6,1248	3,45E-05	1,59782	8,2025	8,1871
**Spectrum 6**	UUM	3	2	5/09/20	146,597	6,1597	6,1248	0,0105181	587,323	8,2025	8,1871
**Spectrum 7**	UM	1	1	4/09/20	0,246333	6,1597	6,1248	-0,001356	-0,0142925	8,2025	8,1871
**Spectrum 8**	UM	2	1	4/09/20	0,206778	6,1597	6,1248	-0,0033256	-0,0341232	8,2025	8,1871
**Spectrum 9**	UM	3	1	4/09/20	0,224908	6,1597	6,1248	0,0041351	0,0472505	8,2025	8,1871
**Spectrum 10**	UM	1	2	5/09/20	0,231778	6,1597	6,1248	0,00447897	0,0462374	8,2025	8,1871
**Spectrum 11**	UM	2	2	5/09/20	0,202496	6,1597	6,1248	-0,0064413	-0,0816478	8,2025	8,1871
**Spectrum 12**	UM	3	2	5/09/20	0,16553	6,1597	6,1248	-0,013265	-0,165944	8,2025	8,1871
**Spectrum 13**	FM	1	2	17/09/20	-89,2093	6,1597	6,1248	0,0106192	402,348	8,2025	8,1871
**Spectrum 14**	FM	1	1	17/09/20	-237,111	6,1597	6,1248	-0,0005349	-17,8567	8,2025	8,1871
**Spectrum 15**	FM	2	1	17/09/20	-55,929	6,1597	6,1248	0,0078845	283,468	8,2025	8,1871
**Spectrum 16**	FM	2	2	17/09/20	-193,229	6,1597	6,1248	-0,0033312	-124,07	8,2025	8,1871
**Spectrum 17**	FM	3	1	17/09/20	-43,2686	6,1597	6,1248	0,016457	496,581	8,2025	8,1871
**Spectrum 18**	FM	3	2	17/09/20	-116,153	6,1597	6,1248	0,00667212	202,866	8,2025	8,1871
**Spectrum 19**	UM	0	3	20/12/19	819,682	6,1597	6,1248	-0,0094097	-269,397	8,2025	8,1871
**Spectrum 20**	UM	0	2	20/12/19	1058,62	6,1597	6,1248	-0,0051522	-175,415	8,2025	8,1871
**Spectrum 21**	UM	0	1	20/12/19	850,398	6,1597	6,1248	-0,0054642	-187,84	8,2025	8,1871
**Spectrum 22**	UUM	0	3	27/12/19	295,876	6,1597	6,1248	9,44E-05	32,692	8,2025	8,1871
**Spectrum 23**	UUM	0	2	27/12/19	233,843	6,1597	6,1248	7,97E-05	22,735	8,2025	8,1871
**Spectrum 24**	UUM	0	1	27/12/19	220,183	6,1597	6,1248	0,00034789	119,124	8,2025	8,1871
**Spectrum 25**	FM	0	3	4/02/20	18,2291	6,1597	6,1248	0,0105699	430,284	8,2025	8,1871
**Spectrum 26**	FM	0	2	28/12/19	250,55	6,1597	6,1248	0,00378438	182,579	8,2025	8,1871
**Spectrum 27**	FM	0	1	28/12/19	342,273	6,1597	6,1248	0,00186202	85,1914	8,2025	8,1871

	Method	Patient	Replicated	Date								
**Spectrum 1**	UUM	1	1	4/09/20	0,0103289	597,47	8,2851	8,2598	0,00522048	301,977	8,3917
**Spectrum 2**	UUM	2	1	4/09/20	0,00523393	543,09	8,2851	8,2598	0,00206267	214,03	8,3917
**Spectrum 3**	UUM	3	1	4/09/20	0,00825812	655,504	8,2851	8,2598	0,00606322	481,279	8,3917
**Spectrum 4**	UUM	1	2	5/09/20	0,0104697	524,247	8,2851	8,2598	0,00731163	366,113	8,3917
**Spectrum 5**	UUM	2	2	5/09/20	0,0155684	720,688	8,2851	8,2598	0,00237649	110,012	8,3917
**Spectrum 6**	UUM	3	2	5/09/20	0,0111984	625,309	8,2851	8,2598	0,0114679	640,356	8,3917
**Spectrum 7**	UM	1	1	4/09/20	0,0302094	0,318405	8,2851	8,2598	0,0161908	0,17065	8,3917
**Spectrum 8**	UM	2	1	4/09/20	0,026911	0,276131	8,2851	8,2598	0,0137895	0,141493	8,3917
**Spectrum 9**	UM	3	1	4/09/20	0,0300394	0,343251	8,2851	8,2598	0,0200319	0,228898	8,3917
**Spectrum 10**	UM	1	2	5/09/20	0,02946	0,304123	8,2851	8,2598	0,018286	0,18877	8,3917
**Spectrum 11**	UM	2	2	5/09/20	0,0239913	0,304108	8,2851	8,2598	0,0102838	0,130355	8,3917
**Spectrum 12**	UM	3	2	5/09/20	0,0165182	0,206642	8,2851	8,2598	0,0238513	0,298378	8,3917
**Spectrum 13**	FM	1	2	17/09/20	0,0023855	90,3833	8,2851	8,2598	0,0132279	501,188	8,3917
**Spectrum 14**	FM	1	1	17/09/20	-0,0006871	-22,9389	8,2851	8,2598	0,015469	516,413	8,3917
**Spectrum 15**	FM	2	1	17/09/20	0,00381963	137,326	8,2851	8,2598	0,0113888	409,456	8,3917
**Spectrum 16**	FM	2	2	17/09/20	0,00093789	34,9318	8,2851	8,2598	0,00807537	300,768	8,3917
**Spectrum 17**	FM	3	1	17/09/20	0,00198372	59,8575	8,2851	8,2598	0,0238711	720,297	8,3917
**Spectrum 18**	FM	3	2	17/09/20	6,67E-07	0,0202847	8,2851	8,2598	0,0193912	589,59	8,3917
**Spectrum 19**	UM	0	3	20/12/19	0,0305374	874,276	8,2851	8,2598	0,00863778	247,297	8,3917
**Spectrum 20**	UM	0	2	20/12/19	0,0327541	1115,17	8,2851	8,2598	0,00990908	337,372	8,3917
**Spectrum 21**	UM	0	1	20/12/19	0,0284305	977,336	8,2851	8,2598	0,00589278	202,572	8,3917
**Spectrum 22**	UUM	0	3	27/12/19	0,00180626	625,607	8,2851	8,2598	0,0011411	395,227	8,3917
**Spectrum 23**	UUM	0	2	27/12/19	0,00187894	535,838	8,2851	8,2598	0,00112842	321,804	8,3917
**Spectrum 24**	UUM	0	1	27/12/19	0,00166682	570,744	8,2851	8,2598	0,00113036	387,05	8,3917
**Spectrum 25**	FM	0	3	4/02/20	0,00342987	139,625	8,2851	8,2598	0,0182105	741,322	8,3917
**Spectrum 26**	FM	0	2	28/12/19	0,0076738	370,225	8,2851	8,2598	0,0105299	508,019	8,3917
**Spectrum 27**	FM	0	1	28/12/19	0,0129052	590,439	8,2851	8,2598	0,0151051	691,089	8,3917

	Method	Patient	Replicated	Date							
**Spectrum 1**	UUM	1	1	4/09/20	8,3745	0,00028221	16,3241	8,6282	8,5897	0,0042031	243,127
**Spectrum 2**	UUM	2	1	4/09/20	8,3745	0,00013843	14,364	8,6282	8,5897	0,00157416	163,34
**Spectrum 3**	UUM	3	1	4/09/20	8,3745	-0,000225	-17,8561	8,6282	8,5897	0,00293654	233,094
**Spectrum 4**	UUM	1	2	5/09/20	8,3745	-0,0007921	-39,6613	8,6282	8,5897	0,00375575	188,06
**Spectrum 5**	UUM	2	2	5/09/20	8,3745	0,00040839	18,9049	8,6282	8,5897	0,00226933	105,051
**Spectrum 6**	UUM	3	2	5/09/20	8,3745	0,00114704	64,0497	8,6282	8,5897	0,00988725	552,095
**Spectrum 7**	UM	1	1	4/09/20	8,3745	0,00744669	0,0784875	8,6282	8,5897	0,00711392	0,0749801
**Spectrum 8**	UM	2	1	4/09/20	8,3745	0,00681895	0,0699687	8,6282	8,5897	0,00481233	0,0493789
**Spectrum 9**	UM	3	1	4/09/20	8,3745	0,00840374	0,0960269	8,6282	8,5897	0,0113174	0,12932
**Spectrum 10**	UM	1	2	5/09/20	8,3745	0,0080182	0,0827737	8,6282	8,5897	0,00944076	0,0974591
**Spectrum 11**	UM	2	2	5/09/20	8,3745	0,00558482	0,0707917	8,6282	8,5897	0,00498161	0,0631457
**Spectrum 12**	UM	3	2	5/09/20	8,3745	0,00313497	0,0392183	8,6282	8,5897	0,0141682	0,177243
**Spectrum 13**	FM	1	2	17/09/20	8,3745	-0,0016961	-64,2628	8,6282	8,5897	0,00937172	355,082
**Spectrum 14**	FM	1	1	17/09/20	8,3745	-0,0048276	-161,163	8,6282	8,5897	0,0077329	258,153
**Spectrum 15**	FM	2	1	17/09/20	8,3745	-0,0006558	-23,5765	8,6282	8,5897	0,00582802	209,532
**Spectrum 16**	FM	2	2	17/09/20	8,3745	-0,0038623	-143,852	8,6282	8,5897	0,00383788	142,942
**Spectrum 17**	FM	3	1	17/09/20	8,3745	-0,0013271	-40,0439	8,6282	8,5897	-0,0024709	-74,5565
**Spectrum 18**	FM	3	2	17/09/20	8,3745	-0,0031827	-96,7698	8,6282	8,5897	-0,0043684	-132,82
**Spectrum 19**	UM	0	3	20/12/19	8,3745	0,00712928	204,109	8,6282	8,5897	-0,0049228	-140,938
**Spectrum 20**	UM	0	2	20/12/19	8,3745	0,0105263	358,386	8,6282	8,5897	0,00050127	17,0667
**Spectrum 21**	UM	0	1	20/12/19	8,3745	0,00820663	282,114	8,6282	8,5897	-0,0003683	-12,6625
**Spectrum 22**	UUM	0	3	27/12/19	8,3745	0,00025771	89,2583	8,6282	8,5897	0,00097762	338,603
**Spectrum 23**	UUM	0	2	27/12/19	8,3745	2,71E-06	0,771993	8,6282	8,5897	0,00146291	417,193
**Spectrum 24**	UUM	0	1	27/12/19	8,3745	9,55E-06	3,26927	8,6282	8,5897	0,00132799	454,724
**Spectrum 25**	FM	0	3	4/02/20	8,3745	0,00053106	21,6187	8,6282	8,5897	0,00935507	380,831
**Spectrum 26**	FM	0	2	28/12/19	8,3745	0,00106688	51,4721	8,6282	8,5897	0,0111021	535,625
**Spectrum 27**	FM	0	1	28/12/19	8,3745	0,00129913	59,4377	8,6282	8,5897	0,0116463	532,841

	Method	Patient	Replicated	Date								
**Spectrum 1**	UUM	1	1	4/09/20	8,2305	8,2049	0,0105982	613,052	8,4729	8,4426	0,00616023
**Spectrum 2**	UUM	2	1	4/09/20	8,2305	8,2049	0,00532815	552,867	8,4729	8,4426	0,0015987
**Spectrum 3**	UUM	3	1	4/09/20	8,2305	8,2049	0,00766756	608,627	8,4729	8,4426	0,00247916
**Spectrum 4**	UUM	1	2	5/09/20	8,2305	8,2049	0,0118478	593,253	8,4729	8,4426	0,00439621
**Spectrum 5**	UUM	2	2	5/09/20	8,2305	8,2049	0,0152805	707,359	8,4729	8,4426	0,00440704
**Spectrum 6**	UUM	3	2	5/09/20	8,2305	8,2049	0,0121114	676,293	8,4729	8,4426	0,00350464
**Spectrum 7**	UM	1	1	4/09/20	8,2305	8,2049	0,0361953	0,381496	8,4729	8,4426	0,0111937
**Spectrum 8**	UM	2	1	4/09/20	8,2305	8,2049	0,0330339	0,338958	8,4729	8,4426	0,0141869
**Spectrum 9**	UM	3	1	4/09/20	8,2305	8,2049	0,0381439	0,435858	8,4729	8,4426	0,0135539
**Spectrum 10**	UM	1	2	5/09/20	8,2305	8,2049	0,0354418	0,365874	8,4729	8,4426	0,0138297
**Spectrum 11**	UM	2	2	5/09/20	8,2305	8,2049	0,0290899	0,368736	8,4729	8,4426	0,00992062
**Spectrum 12**	UM	3	2	5/09/20	8,2305	8,2049	0,0227012	0,28399	8,4729	8,4426	0,00648032
**Spectrum 13**	FM	1	2	17/09/20	8,2305	8,2049	0,00102562	38,8593	8,4729	8,4426	-0,0016237
**Spectrum 14**	FM	1	1	17/09/20	8,2305	8,2049	0,00082441	27,5219	8,4729	8,4426	-0,0058888
**Spectrum 15**	FM	2	1	17/09/20	8,2305	8,2049	0,00313383	112,669	8,4729	8,4426	-0,0012524
**Spectrum 16**	FM	2	2	17/09/20	8,2305	8,2049	-0,0009299	-34,6343	8,4729	8,4426	-0,0030474
**Spectrum 17**	FM	3	1	17/09/20	8,2305	8,2049	0,00232396	70,1242	8,4729	8,4426	-0,0022466
**Spectrum 18**	FM	3	2	17/09/20	8,2305	8,2049	-2,36E-05	-0,71839	8,4729	8,4426	-0,0026523
**Spectrum 19**	UM	0	3	20/12/19	8,2305	8,2049	0,0341811	978,595	8,4729	8,4426	0,00516586
**Spectrum 20**	UM	0	2	20/12/19	8,2305	8,2049	0,0352458	1200,01	8,4729	8,4426	0,00401657
**Spectrum 21**	UM	0	1	20/12/19	8,2305	8,2049	0,0307688	1057,72	8,4729	8,4426	0,00407452
**Spectrum 22**	UUM	0	3	27/12/19	8,2305	8,2049	0,00179236	620,794	8,4729	8,4426	0,00023366
**Spectrum 23**	UUM	0	2	27/12/19	8,2305	8,2049	0,00199433	568,743	8,4729	8,4426	0,00015928
**Spectrum 24**	UUM	0	1	27/12/19	8,2305	8,2049	0,0013527	463,185	8,4729	8,4426	0,00032978
**Spectrum 25**	FM	0	3	4/02/20	8,2305	8,2049	0,00612859	249,486	8,4729	8,4426	0,00473856
**Spectrum 26**	FM	0	2	28/12/19	8,2305	8,2049	0,0106717	514,861	8,4729	8,4426	0,00771191
**Spectrum 27**	FM	0	1	28/12/19	8,2305	8,2049	0,0179462	821,074	8,4729	8,4426	0,00998193

	Method	Patient	Replicated	Date							
**Spectrum 1**	UUM	1	1	4/09/20	356,336	1,8234	1,8096	0,00561184	324,615	1,8582	1,8434
**Spectrum 2**	UUM	2	1	4/09/20	165,887	1,8234	1,8096	0,00228795	237,406	1,8582	1,8434
**Spectrum 3**	UUM	3	1	4/09/20	196,788	1,8234	1,8096	0,00162922	129,322	1,8582	1,8434
**Spectrum 4**	UUM	1	2	5/09/20	220,13	1,8234	1,8096	0,00490369	245,541	1,8582	1,8434
**Spectrum 5**	UUM	2	2	5/09/20	204,009	1,8234	1,8096	0,00301175	139,419	1,8582	1,8434
**Spectrum 6**	UUM	3	2	5/09/20	195,696	1,8234	1,8096	0,00315008	175,898	1,8582	1,8434
**Spectrum 7**	UM	1	1	4/09/20	0,117981	1,8234	1,8096	0,0475873	0,501566	1,8582	1,8434
**Spectrum 8**	UM	2	1	4/09/20	0,14557	1,8234	1,8096	0,0539239	0,553309	1,8582	1,8434
**Spectrum 9**	UM	3	1	4/09/20	0,154877	1,8234	1,8096	0,043414	0,496078	1,8582	1,8434
**Spectrum 10**	UM	1	2	5/09/20	0,142767	1,8234	1,8096	0,0525215	0,542191	1,8582	1,8434
**Spectrum 11**	UM	2	2	5/09/20	0,125751	1,8234	1,8096	0,0452897	0,574081	1,8582	1,8434
**Spectrum 12**	UM	3	2	5/09/20	0,0810683	1,8234	1,8096	0,0481346	0,60216	1,8582	1,8434
**Spectrum 13**	FM	1	2	17/09/20	-61,5194	1,8234	1,8096	0,00308228	116,783	1,8582	1,8434
**Spectrum 14**	FM	1	1	17/09/20	-196,59	1,8234	1,8096	0,00698538	233,198	1,8582	1,8434
**Spectrum 15**	FM	2	1	17/09/20	-45,0264	1,8234	1,8096	0,00465563	167,382	1,8582	1,8434
**Spectrum 16**	FM	2	2	17/09/20	-113,5	1,8234	1,8096	0,00244548	91,082	1,8582	1,8434
**Spectrum 17**	FM	3	1	17/09/20	-67,7908	1,8234	1,8096	0,00482978	145,736	1,8582	1,8434
**Spectrum 18**	FM	3	2	17/09/20	-80,6425	1,8234	1,8096	0,00410912	124,938	1,8582	1,8434
**Spectrum 19**	UM	0	3	20/12/19	147,897	1,8234	1,8096	0,0541649	1550,72	1,8582	1,8434
**Spectrum 20**	UM	0	2	20/12/19	136,751	1,8234	1,8096	0,0586266	1996,05	1,8582	1,8434
**Spectrum 21**	UM	0	1	20/12/19	140,067	1,8234	1,8096	0,0489854	1683,94	1,8582	1,8434
**Spectrum 22**	UUM	0	3	27/12/19	80,9291	1,8234	1,8096	0,00192418	666,45	1,8582	1,8434
**Spectrum 23**	UUM	0	2	27/12/19	45,423	1,8234	1,8096	0,00211858	604,178	1,8582	1,8434
**Spectrum 24**	UUM	0	1	27/12/19	112,921	1,8234	1,8096	0,00213422	730,786	1,8582	1,8434
**Spectrum 25**	FM	0	3	4/02/20	192,9	1,8234	1,8096	0,0153554	625,094	1,8582	1,8434
**Spectrum 26**	FM	0	2	28/12/19	372,063	1,8234	1,8096	0,0139294	672,03	1,8582	1,8434
**Spectrum 27**	FM	0	1	28/12/19	456,694	1,8234	1,8096	0,0263793	1206,91	1,8582	1,8434

	Method	Patient	Replicated	Date							
**Spectrum 1**	UUM	1	1	4/09/20	0,00571184	330,4	2,2398	2,233	0,00810482	468,821	2,2326
**Spectrum 2**	UUM	2	1	4/09/20	0,00175206	181,8	2,2398	2,233	0,00451753	468,754	2,2326
**Spectrum 3**	UUM	3	1	4/09/20	0,00101413	80,4982	2,2398	2,233	0,0058027	460,6	2,2326
**Spectrum 4**	UUM	1	2	5/09/20	0,00410392	205,494	2,2398	2,233	0,00551119	275,96	2,2326
**Spectrum 5**	UUM	2	2	5/09/20	0,00332409	153,878	2,2398	2,233	0,00607315	281,137	2,2326
**Spectrum 6**	UUM	3	2	5/09/20	0,00135868	75,8673	2,2398	2,233	0,00392743	219,304	2,2326
**Spectrum 7**	UM	1	1	4/09/20	0,0552161	0,581973	2,2398	2,233	0,0258027	0,271958	2,2326
**Spectrum 8**	UM	2	1	4/09/20	0,0624427	0,640719	2,2398	2,233	0,022512	0,230994	2,2326
**Spectrum 9**	UM	3	1	4/09/20	0,0465779	0,532231	2,2398	2,233	0,0185166	0,211583	2,2326
**Spectrum 10**	UM	1	2	5/09/20	0,0628885	0,649211	2,2398	2,233	0,0179555	0,185359	2,2326
**Spectrum 11**	UM	2	2	5/09/20	0,0507865	0,643757	2,2398	2,233	0,0148685	0,18847	2,2326
**Spectrum 12**	UM	3	2	5/09/20	0,052416	0,65572	2,2398	2,233	0,0152997	0,191398	2,2326
**Spectrum 13**	FM	1	2	17/09/20	0,00453766	171,926	2,2398	2,233	0,00151584	57,4333	2,2326
**Spectrum 14**	FM	1	1	17/09/20	0,00916956	306,115	2,2398	2,233	0,00436702	145,788	2,2326
**Spectrum 15**	FM	2	1	17/09/20	0,00329596	118,498	2,2398	2,233	0,00228723	82,2319	2,2326
**Spectrum 16**	FM	2	2	17/09/20	0,00432207	160,976	2,2398	2,233	0,00124216	46,2644	2,2326
**Spectrum 17**	FM	3	1	17/09/20	0,0056206	169,598	2,2398	2,233	0,00134715	40,6494	2,2326
**Spectrum 18**	FM	3	2	17/09/20	0,00492795	149,835	2,2398	2,233	0,00346376	105,316	2,2326
**Spectrum 19**	UM	0	3	20/12/19	0,0644961	1846,5	2,2398	2,233	0,0162298	464,655	2,2326
**Spectrum 20**	UM	0	2	20/12/19	0,069274	2358,55	2,2398	2,233	0,0165759	564,355	2,2326
**Spectrum 21**	UM	0	1	20/12/19	0,0587214	2018,63	2,2398	2,233	0,0134523	462,441	2,2326
**Spectrum 22**	UUM	0	3	27/12/19	0,00195304	676,445	2,2398	2,233	0,00107428	372,082	2,2326
**Spectrum 23**	UUM	0	2	27/12/19	0,00247245	705,094	2,2398	2,233	0,00172009	490,536	2,2326
**Spectrum 24**	UUM	0	1	27/12/19	0,0026507	907,636	2,2398	2,233	0,00167564	573,762	2,2326
**Spectrum 25**	FM	0	3	4/02/20	0,0185162	753,768	2,2398	2,233	0,00521815	212,423	2,2326
**Spectrum 26**	FM	0	2	28/12/19	0,0182374	879,867	2,2398	2,233	0,00365206	176,195	2,2326
**Spectrum 27**	FM	0	1	28/12/19	0,0350773	1604,86	2,2398	2,233	0,00673023	307,922	2,2326

	Method	Patient	Replicated	Date							
**Spectrum 1**	UUM	1	1	4/09/20	2,2234	0,00640808	370,673	2,3651	2,3523	0,011352	656,651
**Spectrum 2**	UUM	2	1	4/09/20	2,2234	0,00457753	474,98	2,3651	2,3523	0,00383655	398,093
**Spectrum 3**	UUM	3	1	4/09/20	2,2234	0,0074521	591,524	2,3651	2,3523	0,0124183	985,724
**Spectrum 4**	UUM	1	2	5/09/20	2,2234	0,00635466	318,195	2,3651	2,3523	0,0119198	596,859
**Spectrum 5**	UUM	2	2	5/09/20	2,2234	0,0103469	478,976	2,3651	2,3523	0,0108336	501,504
**Spectrum 6**	UUM	3	2	5/09/20	2,2234	0,00458528	256,038	2,3651	2,3523	0,0180018	1005,21
**Spectrum 7**	UM	1	1	4/09/20	2,2234	0,0177872	0,187476	2,3651	2,3523	0,0513822	0,541565
**Spectrum 8**	UM	2	1	4/09/20	2,2234	0,0194718	0,199799	2,3651	2,3523	0,0480786	0,493331
**Spectrum 9**	UM	3	1	4/09/20	2,2234	0,0180711	0,206493	2,3651	2,3523	0,0549131	0,627474
**Spectrum 10**	UM	1	2	5/09/20	2,2234	0,0184638	0,190605	2,3651	2,3523	0,0493283	0,509226
**Spectrum 11**	UM	2	2	5/09/20	2,2234	0,0158812	0,201306	2,3651	2,3523	0,0380082	0,481782
**Spectrum 12**	UM	3	2	5/09/20	2,2234	0,0163832	0,204953	2,3651	2,3523	0,0483982	0,605457
**Spectrum 13**	FM	1	2	17/09/20	2,2234	0,00153546	58,1766	2,3651	2,3523	0,011915	451,442
**Spectrum 14**	FM	1	1	17/09/20	2,2234	0,00556473	185,772	2,3651	2,3523	0,0165263	551,712
**Spectrum 15**	FM	2	1	17/09/20	2,2234	0,00183507	65,9753	2,3651	2,3523	0,0108787	391,118
**Spectrum 16**	FM	2	2	17/09/20	2,2234	0,00194922	72,599	2,3651	2,3523	0,0115635	430,684
**Spectrum 17**	FM	3	1	17/09/20	2,2234	0,00133737	40,3544	2,3651	2,3523	0,0254784	768,795
**Spectrum 18**	FM	3	2	17/09/20	2,2234	0,00065654	19,9621	2,3651	2,3523	0,021296	647,507
**Spectrum 19**	UM	0	3	20/12/19	2,2234	0,0164183	470,051	2,3651	2,3523	0,0672233	1924,58
**Spectrum 20**	UM	0	2	20/12/19	2,2234	0,017236	586,829	2,3651	2,3523	0,0846638	2882,53
**Spectrum 21**	UM	0	1	20/12/19	2,2234	0,013684	470,406	2,3651	2,3523	0,0613679	2109,6
**Spectrum 22**	UUM	0	3	27/12/19	2,2234	0,00097147	336,473	2,3651	2,3523	0,00319184	1105,51
**Spectrum 23**	UUM	0	2	27/12/19	2,2234	0,00077987	222,403	2,3651	2,3523	0,00380484	1085,07
**Spectrum 24**	UUM	0	1	27/12/19	2,2234	0,00086886	297,511	2,3651	2,3523	0,00361148	1236,62
**Spectrum 25**	FM	0	3	4/02/20	2,2234	0,00302944	123,324	2,3651	2,3523	0,0235573	958,983
**Spectrum 26**	FM	0	2	28/12/19	2,2234	0,00312067	150,558	2,3651	2,3523	0,0180931	872,908
**Spectrum 27**	FM	0	1	28/12/19	2,2234	0,00725905	332,117	2,3651	2,3523	0,0329497	1507,52

	Method	Patient	Replicated	Date							
**Spectrum 1**	UUM	1	1	4/09/20	2,3522	2,3404	0,00784649	453,878	2,4983	2,4674	0,0473154
**Spectrum 2**	UUM	2	1	4/09/20	2,3522	2,3404	0,00270574	280,756	2,4983	2,4674	0,0269545
**Spectrum 3**	UUM	3	1	4/09/20	2,3522	2,3404	0,00749558	594,976	2,4983	2,4674	0,0279398
**Spectrum 4**	UUM	1	2	5/09/20	2,3522	2,3404	0,00683425	342,21	2,4983	2,4674	0,0536526
**Spectrum 5**	UUM	2	2	5/09/20	2,3522	2,3404	0,00685858	317,495	2,4983	2,4674	0,0650096
**Spectrum 6**	UUM	3	2	5/09/20	2,3522	2,3404	0,0116953	653,055	2,4983	2,4674	0,0532117
**Spectrum 7**	UM	1	1	4/09/20	2,3522	2,3404	0,041813	0,440706	2,4983	2,4674	0,0191782
**Spectrum 8**	UM	2	1	4/09/20	2,3522	2,3404	0,0385138	0,395187	2,4983	2,4674	0,0253905
**Spectrum 9**	UM	3	1	4/09/20	2,3522	2,3404	0,0421049	0,481119	2,4983	2,4674	0,0236958
**Spectrum 10**	UM	1	2	5/09/20	2,3522	2,3404	0,0407443	0,420612	2,4983	2,4674	0,0218896
**Spectrum 11**	UM	2	2	5/09/20	2,3522	2,3404	0,0304388	0,385834	2,4983	2,4674	0,0219542
**Spectrum 12**	UM	3	2	5/09/20	2,3522	2,3404	0,0370932	0,464033	2,4983	2,4674	0,0152624
**Spectrum 13**	FM	1	2	17/09/20	2,3522	2,3404	0,0085609	324,361	2,4983	2,4674	0,00020141
**Spectrum 14**	FM	1	1	17/09/20	2,3522	2,3404	0,011068	369,493	2,4983	2,4674	0,00337797
**Spectrum 15**	FM	2	1	17/09/20	2,3522	2,3404	0,00598053	215,015	2,4983	2,4674	-0,0004823
**Spectrum 16**	FM	2	2	17/09/20	2,3522	2,3404	0,00783366	291,765	2,4983	2,4674	0,00145109
**Spectrum 17**	FM	3	1	17/09/20	2,3522	2,3404	0,0159759	482,063	2,4983	2,4674	-0,0047582
**Spectrum 18**	FM	3	2	17/09/20	2,3522	2,3404	0,0151261	459,908	2,4983	2,4674	-0,0029204
**Spectrum 19**	UM	0	3	20/12/19	2,3522	2,3404	0,0466016	1334,19	2,4983	2,4674	0,0081533
**Spectrum 20**	UM	0	2	20/12/19	2,3522	2,3404	0,0575725	1960,15	2,4983	2,4674	0,0135967
**Spectrum 21**	UM	0	1	20/12/19	2,3522	2,3404	0,0424368	1458,82	2,4983	2,4674	0,00677468
**Spectrum 22**	UUM	0	3	27/12/19	2,3522	2,3404	0,00211079	731,082	2,4983	2,4674	0,00487
**Spectrum 23**	UUM	0	2	27/12/19	2,3522	2,3404	0,00240458	685,739	2,4983	2,4674	0,00288705
**Spectrum 24**	UUM	0	1	27/12/19	2,3522	2,3404	0,0024369	834,43	2,4983	2,4674	0,00449797
**Spectrum 25**	FM	0	3	4/02/20	2,3522	2,3404	0,0141346	575,4	2,4983	2,4674	7,30E-05
**Spectrum 26**	FM	0	2	28/12/19	2,3522	2,3404	0,0113442	547,304	2,4983	2,4674	-0,0023009
**Spectrum 27**	FM	0	1	28/12/19	2,3522	2,3404	0,0234746	1074,01	2,4983	2,4674	-0,0012112

	Method	Patient	Replicated	Date							
**Spectrum 1**	UUM	1	1	4/09/20	2736,95	2,4674	2,4335	0,0108817	629,447	2,7377	2,7293
**Spectrum 2**	UUM	2	1	4/09/20	2796,89	2,4674	2,4335	0,00304203	315,651	2,7377	2,7293
**Spectrum 3**	UUM	3	1	4/09/20	2217,77	2,4674	2,4335	0,00498586	395,762	2,7377	2,7293
**Spectrum 4**	UUM	1	2	5/09/20	2686,53	2,4674	2,4335	0,00728987	365,024	2,7377	2,7293
**Spectrum 5**	UUM	2	2	5/09/20	3009,4	2,4674	2,4335	0,00977657	452,574	2,7377	2,7293
**Spectrum 6**	UUM	3	2	5/09/20	2971,3	2,4674	2,4335	0,00953844	532,618	2,7377	2,7293
**Spectrum 7**	UM	1	1	4/09/20	0,202137	2,4674	2,4335	0,0464248	0,489313	2,7377	2,7293
**Spectrum 8**	UM	2	1	4/09/20	0,260529	2,4674	2,4335	0,0506491	0,519706	2,7377	2,7293
**Spectrum 9**	UM	3	1	4/09/20	0,270764	2,4674	2,4335	0,0435219	0,497311	2,7377	2,7293
**Spectrum 10**	UM	1	2	5/09/20	0,225971	2,4674	2,4335	0,0443319	0,457647	2,7377	2,7293
**Spectrum 11**	UM	2	2	5/09/20	0,278286	2,4674	2,4335	0,0408847	0,518244	2,7377	2,7293
**Spectrum 12**	UM	3	2	5/09/20	0,190931	2,4674	2,4335	0,0290899	0,363913	2,7377	2,7293
**Spectrum 13**	FM	1	2	17/09/20	7,63121	2,4674	2,4335	0,00212274	80,4279	2,7377	2,7293
**Spectrum 14**	FM	1	1	17/09/20	112,769	2,4674	2,4335	0,00872858	291,393	2,7377	2,7293
**Spectrum 15**	FM	2	1	17/09/20	-17,3394	2,4674	2,4335	0,0021894	78,7146	2,7377	2,7293
**Spectrum 16**	FM	2	2	17/09/20	54,0459	2,4674	2,4335	0,00237203	88,3464	2,7377	2,7293
**Spectrum 17**	FM	3	1	17/09/20	-143,576	2,4674	2,4335	0,0009027	27,2385	2,7377	2,7293
**Spectrum 18**	FM	3	2	17/09/20	-88,7955	2,4674	2,4335	0,00447201	135,972	2,7377	2,7293
**Spectrum 19**	UM	0	3	20/12/19	233,427	2,4674	2,4335	0,0429479	1229,58	2,7377	2,7293
**Spectrum 20**	UM	0	2	20/12/19	462,923	2,4674	2,4335	0,0511107	1740,15	2,7377	2,7293
**Spectrum 21**	UM	0	1	20/12/19	232,889	2,4674	2,4335	0,0357216	1227,98	2,7377	2,7293
**Spectrum 22**	UUM	0	3	27/12/19	1686,75	2,4674	2,4335	0,00230705	799,058	2,7377	2,7293
**Spectrum 23**	UUM	0	2	27/12/19	823,331	2,4674	2,4335	0,00220759	629,563	2,7377	2,7293
**Spectrum 24**	UUM	0	1	27/12/19	1540,17	2,4674	2,4335	0,00260158	890,816	2,7377	2,7293
**Spectrum 25**	FM	0	3	4/02/20	2,96993	2,4674	2,4335	0,00275523	112,161	2,7377	2,7293
**Spectrum 26**	FM	0	2	28/12/19	-111,009	2,4674	2,4335	0,00251586	121,378	2,7377	2,7293
**Spectrum 27**	FM	0	1	28/12/19	-55,4142	2,4674	2,4335	0,00889965	407,177	2,7377	2,7293

	Method	Patient	Replicated	Date							
**Spectrum 1**	UUM	1	1	4/09/20	0,00093252	53,9411	2,8792	2,8591	0,00359934	208,203	2,9859
**Spectrum 2**	UUM	2	1	4/09/20	-0,0001939	-20,1177	2,8792	2,8591	0,00144231	149,659	2,9859
**Spectrum 3**	UUM	3	1	4/09/20	-0,000405	-32,1486	2,8792	2,8591	0,00033401	26,5128	2,9859
**Spectrum 4**	UUM	1	2	5/09/20	-0,0018914	-94,7094	2,8792	2,8591	0,00016047	8,03497	2,9859
**Spectrum 5**	UUM	2	2	5/09/20	-9,62E-05	-4,45157	2,8792	2,8591	0,00129763	60,0696	2,9859
**Spectrum 6**	UUM	3	2	5/09/20	-0,0014301	-79,8574	2,8792	2,8591	0,00037896	21,1607	2,9859
**Spectrum 7**	UM	1	1	4/09/20	0,00958216	0,100995	2,8792	2,8591	0,0285716	0,301142	2,9859
**Spectrum 8**	UM	2	1	4/09/20	0,0113831	0,116801	2,8792	2,8591	0,0338944	0,347788	2,9859
**Spectrum 9**	UM	3	1	4/09/20	0,00898982	0,102724	2,8792	2,8591	0,0236162	0,269855	2,9859
**Spectrum 10**	UM	1	2	5/09/20	0,010813	0,111625	2,8792	2,8591	0,0306172	0,316068	2,9859
**Spectrum 11**	UM	2	2	5/09/20	0,00949462	0,120351	2,8792	2,8591	0,0270233	0,342541	2,9859
**Spectrum 12**	UM	3	2	5/09/20	0,00716845	0,0896767	2,8792	2,8591	0,0173623	0,217201	2,9859
**Spectrum 13**	FM	1	2	17/09/20	0,00396461	150,214	2,8792	2,8591	0,00296694	112,414	2,9859
**Spectrum 14**	FM	1	1	17/09/20	0,00531746	177,517	2,8792	2,8591	0,00355053	118,53	2,9859
**Spectrum 15**	FM	2	1	17/09/20	0,00303689	109,184	2,8792	2,8591	-0,0014075	-50,6035	2,9859
**Spectrum 16**	FM	2	2	17/09/20	0,00560749	208,852	2,8792	2,8591	0,00720302	268,277	2,9859
**Spectrum 17**	FM	3	1	17/09/20	0,00363756	109,761	2,8792	2,8591	-0,001481	-44,6896	2,9859
**Spectrum 18**	FM	3	2	17/09/20	0,0029224	88,8556	2,8792	2,8591	0,00265078	80,5971	2,9859
**Spectrum 19**	UM	0	3	20/12/19	0,00701892	200,95	2,8792	2,8591	0,0177714	508,788	2,9859
**Spectrum 20**	UM	0	2	20/12/19	0,00689152	234,634	2,8792	2,8591	0,0255999	871,592	2,9859
**Spectrum 21**	UM	0	1	20/12/19	0,00428244	147,215	2,8792	2,8591	0,0176079	605,297	2,9859
**Spectrum 22**	UUM	0	3	27/12/19	2,33E-05	8,05867	2,8792	2,8591	0,00084961	294,268	2,9859
**Spectrum 23**	UUM	0	2	27/12/19	1,86E-05	5,29904	2,8792	2,8591	0,00063966	182,418	2,9859
**Spectrum 24**	UUM	0	1	27/12/19	3,43E-05	11,728	2,8792	2,8591	0,00108924	372,971	2,9859
**Spectrum 25**	FM	0	3	4/02/20	0,00168442	68,5703	2,8792	2,8591	-0,0023106	-94,0598	2,9859
**Spectrum 26**	FM	0	2	28/12/19	-0,0003212	-15,4963	2,8792	2,8591	-0,0001678	-8,0933	2,9859
**Spectrum 27**	FM	0	1	28/12/19	0,00155104	70,963	2,8792	2,8591	0,00224156	102,556	2,9859

	Method	Patient	Replicated	Date							
**Spectrum 1**	UUM	1	1	4/09/20	2,9316	0,00759945	439,588	3,7264	3,7027	0,315565	18253,7
**Spectrum 2**	UUM	2	1	4/09/20	2,9316	0,00349515	362,668	3,7264	3,7027	0,22964	23828,2
**Spectrum 3**	UUM	3	1	4/09/20	2,9316	0,00135484	107,543	3,7264	3,7027	0,246933	19600,7
**Spectrum 4**	UUM	1	2	5/09/20	2,9316	-0,0031746	-158,963	3,7264	3,7027	0,359938	18023,1
**Spectrum 5**	UUM	2	2	5/09/20	2,9316	0,00401875	186,035	3,7264	3,7027	0,735909	34066,5
**Spectrum 6**	UUM	3	2	5/09/20	2,9316	0,00690837	385,758	3,7264	3,7027	0,268843	15012
**Spectrum 7**	UM	1	1	4/09/20	2,9316	0,0936977	0,987566	3,7264	3,7027	0,0191334	0,201664
**Spectrum 8**	UM	2	1	4/09/20	2,9316	0,107551	1,10357	3,7264	3,7027	0,0308975	0,317037
**Spectrum 9**	UM	3	1	4/09/20	2,9316	0,0929771	1,06242	3,7264	3,7027	0,0263722	0,301347
**Spectrum 10**	UM	1	2	5/09/20	2,9316	0,100718	1,03973	3,7264	3,7027	0,0176302	0,182
**Spectrum 11**	UM	2	2	5/09/20	2,9316	0,0934966	1,18514	3,7264	3,7027	0,018725	0,237353
**Spectrum 12**	UM	3	2	5/09/20	2,9316	0,0947857	1,18576	3,7264	3,7027	-0,0109659	-0,137183
**Spectrum 13**	FM	1	2	17/09/20	2,9316	0,0111242	421,483	3,7264	3,7027	0,00471224	178,541
**Spectrum 14**	FM	1	1	17/09/20	2,9316	0,0193606	646,329	3,7264	3,7027	0,00519877	173,555
**Spectrum 15**	FM	2	1	17/09/20	2,9316	0,00733449	263,694	3,7264	3,7027	0,00881303	316,851
**Spectrum 16**	FM	2	2	17/09/20	2,9316	0,0178239	663,852	3,7264	3,7027	0,00410986	153,072
**Spectrum 17**	FM	3	1	17/09/20	2,9316	0,0262559	792,257	3,7264	3,7027	-0,0012546	-37,858
**Spectrum 18**	FM	3	2	17/09/20	2,9316	0,0167334	508,779	3,7264	3,7027	0,00739916	224,972
**Spectrum 19**	UM	0	3	20/12/19	2,9316	0,0772205	2210,8	3,7264	3,7027	0,0414637	1187,09
**Spectrum 20**	UM	0	2	20/12/19	2,9316	0,08771	2986,24	3,7264	3,7027	0,0393857	1340,95
**Spectrum 21**	UM	0	1	20/12/19	2,9316	0,0663245	2280	3,7264	3,7027	0,032014	1100,53
**Spectrum 22**	UUM	0	3	27/12/19	2,9316	0,00176599	611,659	3,7264	3,7027	0,0640464	22182,8
**Spectrum 23**	UUM	0	2	27/12/19	2,9316	0,00156324	445,807	3,7264	3,7027	0,0140249	3999,64
**Spectrum 24**	UUM	0	1	27/12/19	2,9316	0,00218331	747,596	3,7264	3,7027	0,00874418	2994,13
**Spectrum 25**	FM	0	3	4/02/20	2,9316	0,0283261	1153,11	3,7264	3,7027	0,0238647	971,497
**Spectrum 26**	FM	0	2	28/12/19	2,9316	0,0158144	762,973	3,7264	3,7027	0,0196222	946,678
**Spectrum 27**	FM	0	1	28/12/19	2,9316	0,0311004	1422,91	3,7264	3,7027	0,0340964	1559,98

	Method	Patient	Replicated	Date							
**Spectrum 1**	UUM	1	1	4/09/20	3,7405	3,7346	0,0173557	1003,93	3,7671	3,7415	0,0759592
**Spectrum 2**	UUM	2	1	4/09/20	3,7405	3,7346	0,0115883	1202,45	3,7671	3,7415	0,0426101
**Spectrum 3**	UUM	3	1	4/09/20	3,7405	3,7346	0,0123021	976,502	3,7671	3,7415	0,0558887
**Spectrum 4**	UUM	1	2	5/09/20	3,7405	3,7346	0,0181244	907,539	3,7671	3,7415	0,0695721
**Spectrum 5**	UUM	2	2	5/09/20	3,7405	3,7346	0,0337487	1562,29	3,7671	3,7415	0,110898
**Spectrum 6**	UUM	3	2	5/09/20	3,7405	3,7346	0,0139909	781,239	3,7671	3,7415	0,0683553
**Spectrum 7**	UM	1	1	4/09/20	3,7405	3,7346	0,0156913	0,165385	3,7671	3,7415	0,141658
**Spectrum 8**	UM	2	1	4/09/20	3,7405	3,7346	0,0175887	0,180476	3,7671	3,7415	0,136284
**Spectrum 9**	UM	3	1	4/09/20	3,7405	3,7346	0,0144318	0,164907	3,7671	3,7415	0,1176
**Spectrum 10**	UM	1	2	5/09/20	3,7405	3,7346	0,0150855	0,155731	3,7671	3,7415	0,133787
**Spectrum 11**	UM	2	2	5/09/20	3,7405	3,7346	0,0116397	0,147542	3,7671	3,7415	0,104078
**Spectrum 12**	UM	3	2	5/09/20	3,7405	3,7346	-0,0016803	-0,02102	3,7671	3,7415	0,0424194
**Spectrum 13**	FM	1	2	17/09/20	3,7405	3,7346	0,00432656	163,928	3,7671	3,7415	0,0301074
**Spectrum 14**	FM	1	1	17/09/20	3,7405	3,7346	0,00432452	144,369	3,7671	3,7415	0,0379476
**Spectrum 15**	FM	2	1	17/09/20	3,7405	3,7346	0,00359814	129,363	3,7671	3,7415	0,0231332
**Spectrum 16**	FM	2	2	17/09/20	3,7405	3,7346	0,00419067	156,082	3,7671	3,7415	0,0295071
**Spectrum 17**	FM	3	1	17/09/20	3,7405	3,7346	0,00344691	104,009	3,7671	3,7415	0,0461895
**Spectrum 18**	FM	3	2	17/09/20	3,7405	3,7346	0,00429905	130,713	3,7671	3,7415	0,043849
**Spectrum 19**	UM	0	3	20/12/19	3,7405	3,7346	0,0187515	536,849	3,7671	3,7415	0,112658
**Spectrum 20**	UM	0	2	20/12/19	3,7405	3,7346	0,0190637	649,058	3,7671	3,7415	0,115911
**Spectrum 21**	UM	0	1	20/12/19	3,7405	3,7346	0,0150882	518,679	3,7671	3,7415	0,0847566
**Spectrum 22**	UUM	0	3	27/12/19	3,7405	3,7346	0,00459047	1589,93	3,7671	3,7415	0,0135641
**Spectrum 23**	UUM	0	2	27/12/19	3,7405	3,7346	0,0010656	303,89	3,7671	3,7415	0,00688093
**Spectrum 24**	UUM	0	1	27/12/19	3,7405	3,7346	0,00071247	243,961	3,7671	3,7415	0,00557735
**Spectrum 25**	FM	0	3	4/02/20	3,7405	3,7346	0,00771996	314,268	3,7671	3,7415	0,0312365
**Spectrum 26**	FM	0	2	28/12/19	3,7405	3,7346	0,00573191	276,538	3,7671	3,7415	0,0263585
**Spectrum 27**	FM	0	1	28/12/19	3,7405	3,7346	0,00805199	368,395	3,7671	3,7415	0,0369921

	Method	Patient	Replicated	Date							
**Spectrum 1**	UUM	1	1	4/09/20	4393,84	4,0881	4,0611	0,011826	684,071	4,0608	4,0528
**Spectrum 2**	UUM	2	1	4/09/20	4421,37	4,0881	4,0611	0,00636442	660,394	4,0608	4,0528
**Spectrum 3**	UUM	3	1	4/09/20	4436,27	4,0881	4,0611	0,0146042	1159,24	4,0608	4,0528
**Spectrum 4**	UUM	1	2	5/09/20	3483,67	4,0881	4,0611	0,013288	665,368	4,0608	4,0528
**Spectrum 5**	UUM	2	2	5/09/20	5133,67	4,0881	4,0611	0,0173139	801,488	4,0608	4,0528
**Spectrum 6**	UUM	3	2	5/09/20	3816,9	4,0881	4,0611	0,0145171	810,622	4,0608	4,0528
**Spectrum 7**	UM	1	1	4/09/20	1,49306	4,0881	4,0611	0,0530792	0,559451	4,0608	4,0528
**Spectrum 8**	UM	2	1	4/09/20	1,3984	4,0881	4,0611	0,0641952	0,658701	4,0608	4,0528
**Spectrum 9**	UM	3	1	4/09/20	1,34377	4,0881	4,0611	0,0639527	0,730767	4,0608	4,0528
**Spectrum 10**	UM	1	2	5/09/20	1,38111	4,0881	4,0611	0,0476018	0,491404	4,0608	4,0528
**Spectrum 11**	UM	2	2	5/09/20	1,31927	4,0881	4,0611	0,0532453	0,674924	4,0608	4,0528
**Spectrum 12**	UM	3	2	5/09/20	0,530664	4,0881	4,0611	0,0625314	0,782263	4,0608	4,0528
**Spectrum 13**	FM	1	2	17/09/20	1140,73	4,0881	4,0611	0,0117656	445,783	4,0608	4,0528
**Spectrum 14**	FM	1	1	17/09/20	1266,83	4,0881	4,0611	0,0161227	538,239	4,0608	4,0528
**Spectrum 15**	FM	2	1	17/09/20	831,697	4,0881	4,0611	0,00181632	65,3013	4,0608	4,0528
**Spectrum 16**	FM	2	2	17/09/20	1099	4,0881	4,0611	0,0146281	544,825	4,0608	4,0528
**Spectrum 17**	FM	3	1	17/09/20	1393,74	4,0881	4,0611	0,00973905	293,87	4,0608	4,0528
**Spectrum 18**	FM	3	2	17/09/20	1333,23	4,0881	4,0611	0,0160638	488,421	4,0608	4,0528
**Spectrum 19**	UM	0	3	20/12/19	3225,38	4,0881	4,0611	-0,0076325	-218,516	4,0608	4,0528
**Spectrum 20**	UM	0	2	20/12/19	3946,39	4,0881	4,0611	-4,27E-05	-1,4524	4,0608	4,0528
**Spectrum 21**	UM	0	1	20/12/19	2913,62	4,0881	4,0611	-0,0030446	-104,661	4,0608	4,0528
**Spectrum 22**	UUM	0	3	27/12/19	4698,01	4,0881	4,0611	-0,000682	-236,203	4,0608	4,0528
**Spectrum 23**	UUM	0	2	27/12/19	1962,31	4,0881	4,0611	-0,0006832	-194,832	4,0608	4,0528
**Spectrum 24**	UUM	0	1	27/12/19	1909,76	4,0881	4,0611	-0,0004133	-141,518	4,0608	4,0528
**Spectrum 25**	FM	0	3	4/02/20	1271,59	4,0881	4,0611	8,34E-05	3,39506	4,0608	4,0528
**Spectrum 26**	FM	0	2	28/12/19	1271,67	4,0881	4,0611	-0,0031986	-154,315	4,0608	4,0528
**Spectrum 27**	FM	0	1	28/12/19	1692,46	4,0881	4,0611	0,00788017	360,534	4,0608	4,0528

	Method	Patient	Replicated	Date							
**Spectrum 1**	UUM	1	1	4/09/20	0,0035038	202,676	4,052	4,0427	0,00312999	181,053	4,0423
**Spectrum 2**	UUM	2	1	4/09/20	0,00174067	180,618	4,052	4,0427	0,00159807	165,821	4,0423
**Spectrum 3**	UUM	3	1	4/09/20	0,00312924	248,389	4,052	4,0427	0,00114359	90,7745	4,0423
**Spectrum 4**	UUM	1	2	5/09/20	-0,0012628	-63,2302	4,052	4,0427	-0,0022325	-111,786	4,0423
**Spectrum 5**	UUM	2	2	5/09/20	0,00360016	166,657	4,052	4,0427	0,00284217	131,569	4,0423
**Spectrum 6**	UUM	3	2	5/09/20	0,00311619	174,006	4,052	4,0427	0,00265766	148,402	4,0423
**Spectrum 7**	UM	1	1	4/09/20	0,0179131	0,188803	4,052	4,0427	0,0212481	0,223953	4,0423
**Spectrum 8**	UM	2	1	4/09/20	0,0207028	0,21243	4,052	4,0427	0,0236412	0,24258	4,0423
**Spectrum 9**	UM	3	1	4/09/20	0,0169189	0,193327	4,052	4,0427	0,0194712	0,222491	4,0423
**Spectrum 10**	UM	1	2	5/09/20	0,0180111	0,185932	4,052	4,0427	0,0208206	0,214936	4,0423
**Spectrum 11**	UM	2	2	5/09/20	0,0184502	0,23387	4,052	4,0427	0,0208144	0,263838	4,0423
**Spectrum 12**	UM	3	2	5/09/20	0,0140956	0,176335	4,052	4,0427	0,00831633	0,104037	4,0423
**Spectrum 13**	FM	1	2	17/09/20	0,00248869	94,2932	4,052	4,0427	0,00152902	57,9326	4,0423
**Spectrum 14**	FM	1	1	17/09/20	0,00485483	162,073	4,052	4,0427	0,00503005	167,922	4,0423
**Spectrum 15**	FM	2	1	17/09/20	0,00029108	10,4652	4,052	4,0427	0,00023283	8,37089	4,0423
**Spectrum 16**	FM	2	2	17/09/20	0,00404652	150,713	4,052	4,0427	0,00474569	176,754	4,0423
**Spectrum 17**	FM	3	1	17/09/20	-0,0006016	-18,152	4,052	4,0427	0,00086632	26,1407	4,0423
**Spectrum 18**	FM	3	2	17/09/20	0,00184366	56,0565	4,052	4,0427	0,00142866	43,4385	4,0423
**Spectrum 19**	UM	0	3	20/12/19	-0,0018436	-52,7825	4,052	4,0427	-0,0016984	-48,624	4,0423
**Spectrum 20**	UM	0	2	20/12/19	-0,0005579	-18,9956	4,052	4,0427	-0,0009093	-30,959	4,0423
**Spectrum 21**	UM	0	1	20/12/19	-0,0013811	-47,4785	4,052	4,0427	-0,0017508	-60,1868	4,0423
**Spectrum 22**	UUM	0	3	27/12/19	-0,0001333	-46,1665	4,052	4,0427	-0,0002765	-95,7806	4,0423
**Spectrum 23**	UUM	0	2	27/12/19	-0,0002843	-81,0896	4,052	4,0427	-0,0001078	-30,7375	4,0423
**Spectrum 24**	UUM	0	1	27/12/19	-0,0001909	-65,3611	4,052	4,0427	-0,0003634	-124,424	4,0423
**Spectrum 25**	FM	0	3	4/02/20	0,00030651	12,4776	4,052	4,0427	-0,000157	-6,39303	4,0423
**Spectrum 26**	FM	0	2	28/12/19	-0,0010545	-50,8725	4,052	4,0427	-0,0020747	-100,096	4,0423
**Spectrum 27**	FM	0	1	28/12/19	0,00179352	82,0572	4,052	4,0427	0,00197618	90,4145	4,0423

	Method	Patient	Replicated	Date							
**Spectrum 1**	UUM	1	1	4/09/20	4,0356	0,0018007	104,161	4,118	4,0972	0,0342483	1981,08
**Spectrum 2**	UUM	2	1	4/09/20	4,0356	0,00115411	119,755	4,118	4,0972	0,012352	1281,69
**Spectrum 3**	UUM	3	1	4/09/20	4,0356	0,00028675	22,761	4,118	4,0972	0,0142675	1132,51
**Spectrum 4**	UUM	1	2	5/09/20	4,0356	-0,0016444	-82,3393	4,118	4,0972	0,0306775	1536,1
**Spectrum 5**	UUM	2	2	5/09/20	4,0356	0,00098654	45,6686	4,118	4,0972	0,018128	839,177
**Spectrum 6**	UUM	3	2	5/09/20	4,0356	0,00263172	146,953	4,118	4,0972	0,019459	1086,58
**Spectrum 7**	UM	1	1	4/09/20	4,0356	0,0154457	0,162796	4,118	4,0972	0,0665656	0,701596
**Spectrum 8**	UM	2	1	4/09/20	4,0356	0,016333	0,167592	4,118	4,0972	0,0649996	0,666955
**Spectrum 9**	UM	3	1	4/09/20	4,0356	0,0138193	0,157908	4,118	4,0972	0,0613827	0,701401
**Spectrum 10**	UM	1	2	5/09/20	4,0356	0,0154813	0,159817	4,118	4,0972	0,0618119	0,638098
**Spectrum 11**	UM	2	2	5/09/20	4,0356	0,0132618	0,168104	4,118	4,0972	0,0522448	0,662241
**Spectrum 12**	UM	3	2	5/09/20	4,0356	0,00625936	0,0783042	4,118	4,0972	0,0444501	0,556068
**Spectrum 13**	FM	1	2	17/09/20	4,0356	0,00300465	113,842	4,118	4,0972	0,018979	719,088
**Spectrum 14**	FM	1	1	17/09/20	4,0356	0,00490812	163,852	4,118	4,0972	0,0298814	997,555
**Spectrum 15**	FM	2	1	17/09/20	4,0356	0,00179725	64,6158	4,118	4,0972	0,0122159	439,194
**Spectrum 16**	FM	2	2	17/09/20	4,0356	0,00370959	138,164	4,118	4,0972	0,0180357	671,741
**Spectrum 17**	FM	3	1	17/09/20	4,0356	0,00354901	107,089	4,118	4,0972	0,0167778	506,262
**Spectrum 18**	FM	3	2	17/09/20	4,0356	0,00455916	138,621	4,118	4,0972	0,0274927	835,916
**Spectrum 19**	UM	0	3	20/12/19	4,0356	-0,0010049	-28,7704	4,118	4,0972	0,00629851	180,324
**Spectrum 20**	UM	0	2	20/12/19	4,0356	-0,0007716	-26,2721	4,118	4,0972	0,0102165	347,837
**Spectrum 21**	UM	0	1	20/12/19	4,0356	-0,0016191	-55,6596	4,118	4,0972	0,00931805	320,321
**Spectrum 22**	UUM	0	3	27/12/19	4,0356	-0,0001954	-67,6738	4,118	4,0972	-9,32E-05	-32,2711
**Spectrum 23**	UUM	0	2	27/12/19	4,0356	-0,0003043	-86,7912	4,118	4,0972	4,54E-05	12,934
**Spectrum 24**	UUM	0	1	27/12/19	4,0356	-0,0002551	-87,3486	4,118	4,0972	0,00019113	65,4466
**Spectrum 25**	FM	0	3	4/02/20	4,0356	-0,0012753	-51,9161	4,118	4,0972	0,00472177	192,216
**Spectrum 26**	FM	0	2	28/12/19	4,0356	-0,0009502	-45,841	4,118	4,0972	0,00174235	84,0602
**Spectrum 27**	FM	0	1	28/12/19	4,0356	0,00107897	49,3649	4,118	4,0972	0,0119308	545,858

	Method	Patient	Replicated	Date							
**Spectrum 1**	UUM	1	1	4/09/20	4,0972	4,0906	0,00305736	176,852	4,1289	4,1184	0,0232551
**Spectrum 2**	UUM	2	1	4/09/20	4,0972	4,0906	0,00117774	122,207	4,1289	4,1184	0,0069449
**Spectrum 3**	UUM	3	1	4/09/20	4,0972	4,0906	0,00171141	135,847	4,1289	4,1184	0,00943495
**Spectrum 4**	UUM	1	2	5/09/20	4,0972	4,0906	0,00305712	153,078	4,1289	4,1184	0,0176395
**Spectrum 5**	UUM	2	2	5/09/20	4,0972	4,0906	0,00339371	157,101	4,1289	4,1184	0,00892296
**Spectrum 6**	UUM	3	2	5/09/20	4,0972	4,0906	0,00230204	128,544	4,1289	4,1184	0,0131019
**Spectrum 7**	UM	1	1	4/09/20	4,0972	4,0906	0,0162762	0,17155	4,1289	4,1184	0,0390284
**Spectrum 8**	UM	2	1	4/09/20	4,0972	4,0906	0,0171002	0,175463	4,1289	4,1184	0,0346181
**Spectrum 9**	UM	3	1	4/09/20	4,0972	4,0906	0,0136276	0,155719	4,1289	4,1184	0,0355149
**Spectrum 10**	UM	1	2	5/09/20	4,0972	4,0906	0,0146089	0,150811	4,1289	4,1184	0,0350509
**Spectrum 11**	UM	2	2	5/09/20	4,0972	4,0906	0,0146859	0,186155	4,1289	4,1184	0,0251359
**Spectrum 12**	UM	3	2	5/09/20	4,0972	4,0906	0,0110034	0,137651	4,1289	4,1184	0,0210983
**Spectrum 13**	FM	1	2	17/09/20	4,0972	4,0906	0,00113529	43,0147	4,1289	4,1184	0,0141166
**Spectrum 14**	FM	1	1	17/09/20	4,0972	4,0906	0,00373611	124,725	4,1289	4,1184	0,0209582
**Spectrum 15**	FM	2	1	17/09/20	4,0972	4,0906	-0,0004529	-16,2845	4,1289	4,1184	0,0089812
**Spectrum 16**	FM	2	2	17/09/20	4,0972	4,0906	0,00272789	101,6	4,1289	4,1184	0,0103285
**Spectrum 17**	FM	3	1	17/09/20	4,0972	4,0906	-0,0013335	-40,2374	4,1289	4,1184	0,0158307
**Spectrum 18**	FM	3	2	17/09/20	4,0972	4,0906	0,0005421	16,4825	4,1289	4,1184	0,0203931
**Spectrum 19**	UM	0	3	20/12/19	4,0972	4,0906	0,000485	13,8853	4,1289	4,1184	0,00664104
**Spectrum 20**	UM	0	2	20/12/19	4,0972	4,0906	0,00118492	40,3426	4,1289	4,1184	0,0100091
**Spectrum 21**	UM	0	1	20/12/19	4,0972	4,0906	0,00094048	32,3303	4,1289	4,1184	0,00785756
**Spectrum 22**	UUM	0	3	27/12/19	4,0972	4,0906	-0,0001207	-41,8081	4,1289	4,1184	8,59E-05
**Spectrum 23**	UUM	0	2	27/12/19	4,0972	4,0906	-6,68E-06	-1,90608	4,1289	4,1184	0,00021566
**Spectrum 24**	UUM	0	1	27/12/19	4,0972	4,0906	-0,0001572	-53,8183	4,1289	4,1184	0,00020405
**Spectrum 25**	FM	0	3	4/02/20	4,0972	4,0906	3,17E-05	1,28934	4,1289	4,1184	0,0058684
**Spectrum 26**	FM	0	2	28/12/19	4,0972	4,0906	-0,0015424	-74,4143	4,1289	4,1184	0,00324686
**Spectrum 27**	FM	0	1	28/12/19	4,0972	4,0906	0,00075882	34,7176	4,1289	4,1184	0,00990693

	Method	Patient	Replicated	Date							
**Spectrum 1**	UUM	1	1	4/09/20	1345,19	4,1407	4,1291	0,0104338	603,543	4,1519	4,1423
**Spectrum 2**	UUM	2	1	4/09/20	720,626	4,1407	4,1291	0,00388122	402,729	4,1519	4,1423
**Spectrum 3**	UUM	3	1	4/09/20	748,917	4,1407	4,1291	0,00499513	396,498	4,1519	4,1423
**Spectrum 4**	UUM	1	2	5/09/20	883,258	4,1407	4,1291	0,00615061	307,978	4,1519	4,1423
**Spectrum 5**	UUM	2	2	5/09/20	413,059	4,1407	4,1291	0,00626406	289,974	4,1519	4,1423
**Spectrum 6**	UUM	3	2	5/09/20	731,597	4,1407	4,1291	0,0077787	434,356	4,1519	4,1423
**Spectrum 7**	UM	1	1	4/09/20	0,411356	4,1407	4,1291	0,0377543	0,397928	4,1519	4,1423
**Spectrum 8**	UM	2	1	4/09/20	0,355213	4,1407	4,1291	0,0403226	0,413747	4,1519	4,1423
**Spectrum 9**	UM	3	1	4/09/20	0,405818	4,1407	4,1291	0,0331547	0,378849	4,1519	4,1423
**Spectrum 10**	UM	1	2	5/09/20	0,361838	4,1407	4,1291	0,0366413	0,378256	4,1519	4,1423
**Spectrum 11**	UM	2	2	5/09/20	0,318616	4,1407	4,1291	0,027818	0,352614	4,1519	4,1423
**Spectrum 12**	UM	3	2	5/09/20	0,263938	4,1407	4,1291	0,0195099	0,244067	4,1519	4,1423
**Spectrum 13**	FM	1	2	17/09/20	534,858	4,1407	4,1291	0,0083932	318,008	4,1519	4,1423
**Spectrum 14**	FM	1	1	17/09/20	699,665	4,1407	4,1291	0,0144046	480,879	4,1519	4,1423
**Spectrum 15**	FM	2	1	17/09/20	322,897	4,1407	4,1291	0,00282043	101,402	4,1519	4,1423
**Spectrum 16**	FM	2	2	17/09/20	384,686	4,1407	4,1291	0,00879195	327,457	4,1519	4,1423
**Spectrum 17**	FM	3	1	17/09/20	477,683	4,1407	4,1291	0,0089412	269,796	4,1519	4,1423
**Spectrum 18**	FM	3	2	17/09/20	620,051	4,1407	4,1291	0,0119453	363,197	4,1519	4,1423
**Spectrum 19**	UM	0	3	20/12/19	190,131	4,1407	4,1291	0,00954723	273,334	4,1519	4,1423
**Spectrum 20**	UM	0	2	20/12/19	340,776	4,1407	4,1291	0,0155147	528,224	4,1519	4,1423
**Spectrum 21**	UM	0	1	20/12/19	270,114	4,1407	4,1291	0,0104636	359,7	4,1519	4,1423
**Spectrum 22**	UUM	0	3	27/12/19	29,7481	4,1407	4,1291	0,00019402	67,1996	4,1519	4,1423
**Spectrum 23**	UUM	0	2	27/12/19	61,5021	4,1407	4,1291	3,96E-05	11,3004	4,1519	4,1423
**Spectrum 24**	UUM	0	1	27/12/19	69,8693	4,1407	4,1291	6,37E-05	21,8146	4,1519	4,1423
**Spectrum 25**	FM	0	3	4/02/20	238,894	4,1407	4,1291	0,00424008	172,607	4,1519	4,1423
**Spectrum 26**	FM	0	2	28/12/19	156,646	4,1407	4,1291	0,00242613	117,049	4,1519	4,1423
**Spectrum 27**	FM	0	1	28/12/19	453,262	4,1407	4,1291	0,00803974	367,835	4,1519	4,1423

	Method	Patient	Replicated	Date							
**Spectrum 1**	UUM	1	1	4/09/20	0,00202868	117,348	4,2759	4,2442	0,0129041	746,435	5,6375
**Spectrum 2**	UUM	2	1	4/09/20	0,00107299	111,337	4,2759	4,2442	0,00367592	381,426	5,6375
**Spectrum 3**	UUM	3	1	4/09/20	1,50E-05	1,1884	4,2759	4,2442	0,00841545	667,992	5,6375
**Spectrum 4**	UUM	1	2	5/09/20	-0,0005464	-27,3583	4,2759	4,2442	0,0127768	639,769	5,6375
**Spectrum 5**	UUM	2	2	5/09/20	0,00157284	72,8094	4,2759	4,2442	0,0121902	564,305	5,6375
**Spectrum 6**	UUM	3	2	5/09/20	0,00158675	88,6029	4,2759	4,2442	0,0111978	625,277	5,6375
**Spectrum 7**	UM	1	1	4/09/20	0,0246847	0,260175	4,2759	4,2442	0,103562	1,09153	5,6375
**Spectrum 8**	UM	2	1	4/09/20	0,0281953	0,28931	4,2759	4,2442	0,114511	1,17498	5,6375
**Spectrum 9**	UM	3	1	4/09/20	0,020397	0,23307	4,2759	4,2442	0,0994816	1,13674	5,6375
**Spectrum 10**	UM	1	2	5/09/20	0,0229102	0,236507	4,2759	4,2442	0,0919949	0,949684	5,6375
**Spectrum 11**	UM	2	2	5/09/20	0,0209817	0,265959	4,2759	4,2442	0,0886092	1,12319	5,6375
**Spectrum 12**	UM	3	2	5/09/20	0,00852479	0,106645	4,2759	4,2442	0,0611178	0,764579	5,6375
**Spectrum 13**	FM	1	2	17/09/20	0,00310989	117,829	4,2759	4,2442	0,0169162	640,934	5,6375
**Spectrum 14**	FM	1	1	17/09/20	0,00827937	276,397	4,2759	4,2442	0,0329244	1099,14	5,6375
**Spectrum 15**	FM	2	1	17/09/20	-0,0002804	-10,0801	4,2759	4,2442	0,00177754	63,9069	5,6375
**Spectrum 16**	FM	2	2	17/09/20	0,00532818	198,448	4,2759	4,2442	0,0201259	749,592	5,6375
**Spectrum 17**	FM	3	1	17/09/20	0,0004074	12,293	4,2759	4,2442	0,022492	678,683	5,6375
**Spectrum 18**	FM	3	2	17/09/20	0,00176098	53,5426	4,2759	4,2442	0,0290437	883,074	5,6375
**Spectrum 19**	UM	0	3	20/12/19	0,00681271	195,046	4,2759	4,2442	-0,0024576	-70,361	5,6375
**Spectrum 20**	UM	0	2	20/12/19	0,00897583	305,598	4,2759	4,2442	-0,0101847	-346,755	5,6375
**Spectrum 21**	UM	0	1	20/12/19	0,00625342	214,97	4,2759	4,2442	-0,0101623	-349,344	5,6375
**Spectrum 22**	UUM	0	3	27/12/19	-6,20E-05	-21,4736	4,2759	4,2442	-0,0007027	-243,379	5,6375
**Spectrum 23**	UUM	0	2	27/12/19	-0,0001398	-39,8629	4,2759	4,2442	-0,0011661	-332,536	5,6375
**Spectrum 24**	UUM	0	1	27/12/19	3,39E-05	11,5946	4,2759	4,2442	-0,0007493	-256,561	5,6375
**Spectrum 25**	FM	0	3	4/02/20	0,00301812	122,863	4,2759	4,2442	-0,0055211	-224,757	5,6375
**Spectrum 26**	FM	0	2	28/12/19	0,0017035	82,1859	4,2759	4,2442	-0,0094619	-456,49	5,6375
**Spectrum 27**	FM	0	1	28/12/19	0,00466726	213,537	4,2759	4,2442	-0,004145	-189,643	5,6375

	Method	Patient	Replicated	Date							
**Spectrum 1**	UUM	1	1	4/09/20	5,6064	0,00554433	320,71	0,9229	0,8599	0,0286213	1655,59
**Spectrum 2**	UUM	2	1	4/09/20	5,6064	0,00228116	236,701	0,9229	0,8599	0,0124203	1288,78
**Spectrum 3**	UUM	3	1	4/09/20	5,6064	0,00336778	267,324	0,9229	0,8599	0,0148462	1178,45
**Spectrum 4**	UUM	1	2	5/09/20	5,6064	0,0109986	550,732	0,9229	0,8599	0,0228448	1143,9
**Spectrum 5**	UUM	2	2	5/09/20	5,6064	0,00529193	244,972	0,9229	0,8599	0,0267638	1238,94
**Spectrum 6**	UUM	3	2	5/09/20	5,6064	0,00100392	56,058	0,9229	0,8599	0,0177185	989,386
**Spectrum 7**	UM	1	1	4/09/20	5,6064	0,0179635	0,189334	0,9229	0,8599	0,522212	5,50407
**Spectrum 8**	UM	2	1	4/09/20	5,6064	0,0185676	0,190521	0,9229	0,8599	0,52452	5,38205
**Spectrum 9**	UM	3	1	4/09/20	5,6064	0,0142323	0,162628	0,9229	0,8599	0,441762	5,04788
**Spectrum 10**	UM	1	2	5/09/20	5,6064	0,0254342	0,262562	0,9229	0,8599	0,56474	5,82994
**Spectrum 11**	UM	2	2	5/09/20	5,6064	0,0221931	0,281314	0,9229	0,8599	0,425637	5,39526
**Spectrum 12**	UM	3	2	5/09/20	5,6064	0,025998	0,325232	0,9229	0,8599	0,484909	6,06617
**Spectrum 13**	FM	1	2	17/09/20	5,6064	-0,0023315	-88,3385	0,9229	0,8599	0,0622501	2358,58
**Spectrum 14**	FM	1	1	17/09/20	5,6064	-0,0194168	-648,208	0,9229	0,8599	0,102433	3419,59
**Spectrum 15**	FM	2	1	17/09/20	5,6064	0,00879853	316,33	0,9229	0,8599	0,0874338	3143,47
**Spectrum 16**	FM	2	2	17/09/20	5,6064	-0,0134573	-501,22	0,9229	0,8599	0,0501982	1869,64
**Spectrum 17**	FM	3	1	17/09/20	5,6064	0,00370288	111,732	0,9229	0,8599	0,0497769	1501,99
**Spectrum 18**	FM	3	2	17/09/20	5,6064	-0,0065214	-198,285	0,9229	0,8599	0,0484338	1472,63
**Spectrum 19**	UM	0	3	20/12/19	5,6064	0,0143609	411,147	0,9229	0,8599	0,543197	15551,6
**Spectrum 20**	UM	0	2	20/12/19	5,6064	0,00749041	255,024	0,9229	0,8599	0,535069	18217,3
**Spectrum 21**	UM	0	1	20/12/19	5,6064	0,0104742	360,066	0,9229	0,8599	0,455964	15674,4
**Spectrum 22**	UUM	0	3	27/12/19	5,6064	0,00060321	208,925	0,9229	0,8599	0,00727672	2520,33
**Spectrum 23**	UUM	0	2	27/12/19	5,6064	0,00027848	79,4159	0,9229	0,8599	0,0060284	1719,18
**Spectrum 24**	UUM	0	1	27/12/19	5,6064	0,00020108	68,8523	0,9229	0,8599	0,00363485	1244,62
**Spectrum 25**	FM	0	3	4/02/20	5,6064	-0,001789	-72,8289	0,9229	0,8599	0,0571508	2326,52
**Spectrum 26**	FM	0	2	28/12/19	5,6064	-0,0004746	-22,8955	0,9229	0,8599	0,103021	4970,27
**Spectrum 27**	FM	0	1	28/12/19	5,6064	3,61E-05	1,65108	0,9229	0,8599	0,193883	8870,54

	Method	Patient	Replicated	Date								
**Spectrum 1**	UUM	1	1	4/09/20	1,1007	1,0649	0,0138313	800,07	1,3109	1,2737	0,0292972
**Spectrum 2**	UUM	2	1	4/09/20	1,1007	1,0649	0,0066175	686,654	1,3109	1,2737	0,0136363
**Spectrum 3**	UUM	3	1	4/09/20	1,1007	1,0649	0,0110814	879,61	1,3109	1,2737	0,0227321
**Spectrum 4**	UUM	1	2	5/09/20	1,1007	1,0649	0,0106999	535,776	1,3109	1,2737	0,0303864
**Spectrum 5**	UUM	2	2	5/09/20	1,1007	1,0649	0,0138095	639,267	1,3109	1,2737	0,0305342
**Spectrum 6**	UUM	3	2	5/09/20	1,1007	1,0649	0,0106427	594,282	1,3109	1,2737	0,0208533
**Spectrum 7**	UM	1	1	4/09/20	1,1007	1,0649	0,0426608	0,449642	1,3109	1,2737	0,0826564
**Spectrum 8**	UM	2	1	4/09/20	1,1007	1,0649	0,0435206	0,446561	1,3109	1,2737	0,0820426
**Spectrum 9**	UM	3	1	4/09/20	1,1007	1,0649	0,0466764	0,533356	1,3109	1,2737	0,0879472
**Spectrum 10**	UM	1	2	5/09/20	1,1007	1,0649	0,0493957	0,509922	1,3109	1,2737	0,0921484
**Spectrum 11**	UM	2	2	5/09/20	1,1007	1,0649	0,0384554	0,487451	1,3109	1,2737	0,0709941
**Spectrum 12**	UM	3	2	5/09/20	1,1007	1,0649	0,0381373	0,477094	1,3109	1,2737	0,0691167
**Spectrum 13**	FM	1	2	17/09/20	1,1007	1,0649	0,00219081	83,0068	1,3109	1,2737	0,0810509
**Spectrum 14**	FM	1	1	17/09/20	1,1007	1,0649	0,0149257	498,276	1,3109	1,2737	0,115975
**Spectrum 15**	FM	2	1	17/09/20	1,1007	1,0649	0,00733233	263,616	1,3109	1,2737	0,106782
**Spectrum 16**	FM	2	2	17/09/20	1,1007	1,0649	0,00442923	164,967	1,3109	1,2737	0,0591898
**Spectrum 17**	FM	3	1	17/09/20	1,1007	1,0649	0,00343065	103,518	1,3109	1,2737	0,0481655
**Spectrum 18**	FM	3	2	17/09/20	1,1007	1,0649	0,00241862	73,5381	1,3109	1,2737	0,0687411
**Spectrum 19**	UM	0	3	20/12/19	1,1007	1,0649	0,0160852	460,513	1,3109	1,2737	0,0524907
**Spectrum 20**	UM	0	2	20/12/19	1,1007	1,0649	0,020254	689,582	1,3109	1,2737	0,0577518
**Spectrum 21**	UM	0	1	20/12/19	1,1007	1,0649	0,012897	443,353	1,3109	1,2737	0,044082
**Spectrum 22**	UUM	0	3	27/12/19	1,1007	1,0649	0,0444759	15404,5	1,3109	1,2737	0,0516192
**Spectrum 23**	UUM	0	2	27/12/19	1,1007	1,0649	0,0424145	12095,8	1,3109	1,2737	0,0398951
**Spectrum 24**	UUM	0	1	27/12/19	1,1007	1,0649	0,0354362	12133,9	1,3109	1,2737	0,0393787
**Spectrum 25**	FM	0	3	4/02/20	1,1007	1,0649	-0,0013616	-55,4289	1,3109	1,2737	0,0183292
**Spectrum 26**	FM	0	2	28/12/19	1,1007	1,0649	-0,000772	-37,2468	1,3109	1,2737	0,0262154
**Spectrum 27**	FM	0	1	28/12/19	1,1007	1,0649	0,00274679	125,671	1,3109	1,2737	0,0418286

	Method	Patient	Replicated	Date							
**Spectrum 1**	UUM	1	1	4/09/20	1694,69	2,1356	2,1114	0,0166153	961,104	2,1498	2,1359
**Spectrum 2**	UUM	2	1	4/09/20	1414,95	2,1356	2,1114	0,00717571	744,576	2,1498	2,1359
**Spectrum 3**	UUM	3	1	4/09/20	1804,4	2,1356	2,1114	0,0133195	1057,26	2,1498	2,1359
**Spectrum 4**	UUM	1	2	5/09/20	1521,53	2,1356	2,1114	0,0158455	793,427	2,1498	2,1359
**Spectrum 5**	UUM	2	2	5/09/20	1413,48	2,1356	2,1114	0,0171032	791,737	2,1498	2,1359
**Spectrum 6**	UUM	3	2	5/09/20	1164,43	2,1356	2,1114	0,0209146	1167,85	2,1498	2,1359
**Spectrum 7**	UM	1	1	4/09/20	0,871192	2,1356	2,1114	0,1105	1,16466	2,1498	2,1359
**Spectrum 8**	UM	2	1	4/09/20	0,841832	2,1356	2,1114	0,111832	1,1475	2,1498	2,1359
**Spectrum 9**	UM	3	1	4/09/20	1,00494	2,1356	2,1114	0,108677	1,24182	2,1498	2,1359
**Spectrum 10**	UM	1	2	5/09/20	0,951268	2,1356	2,1114	0,112329	1,1596	2,1498	2,1359
**Spectrum 11**	UM	2	2	5/09/20	0,899903	2,1356	2,1114	0,0893724	1,13286	2,1498	2,1359
**Spectrum 12**	UM	3	2	5/09/20	0,864644	2,1356	2,1114	0,103249	1,29163	2,1498	2,1359
**Spectrum 13**	FM	1	2	17/09/20	3070,91	2,1356	2,1114	0,0160756	609,082	2,1498	2,1359
**Spectrum 14**	FM	1	1	17/09/20	3871,67	2,1356	2,1114	0,0236993	791,174	2,1498	2,1359
**Spectrum 15**	FM	2	1	17/09/20	3839,08	2,1356	2,1114	0,012035	432,688	2,1498	2,1359
**Spectrum 16**	FM	2	2	17/09/20	2204,53	2,1356	2,1114	0,0144749	539,12	2,1498	2,1359
**Spectrum 17**	FM	3	1	17/09/20	1453,37	2,1356	2,1114	0,026975	813,955	2,1498	2,1359
**Spectrum 18**	FM	3	2	17/09/20	2090,07	2,1356	2,1114	0,0241561	734,468	2,1498	2,1359
**Spectrum 19**	UM	0	3	20/12/19	1502,79	2,1356	2,1114	0,108784	3114,45	2,1498	2,1359
**Spectrum 20**	UM	0	2	20/12/19	1966,26	2,1356	2,1114	0,125135	4260,44	2,1498	2,1359
**Spectrum 21**	UM	0	1	20/12/19	1515,38	2,1356	2,1114	0,096071	3302,57	2,1498	2,1359
**Spectrum 22**	UUM	0	3	27/12/19	17878,5	2,1356	2,1114	0,0040228	1393,32	2,1498	2,1359
**Spectrum 23**	UUM	0	2	27/12/19	11377,3	2,1356	2,1114	0,00458191	1306,67	2,1498	2,1359
**Spectrum 24**	UUM	0	1	27/12/19	13483,8	2,1356	2,1114	0,00467618	1601,19	2,1498	2,1359
**Spectrum 25**	FM	0	3	4/02/20	746,154	2,1356	2,1114	0,0191454	779,382	2,1498	2,1359
**Spectrum 26**	FM	0	2	28/12/19	1264,77	2,1356	2,1114	0,024984	1205,36	2,1498	2,1359
**Spectrum 27**	FM	0	1	28/12/19	1913,74	2,1356	2,1114	0,0469712	2149,03	2,1498	2,1359

	Method	Patient	Replicated	Date							
**Spectrum 1**	UUM	1	1	4/09/20	0,0186897	1081,1	2,1929	2,1506	0,0194141	1123	2,2706
**Spectrum 2**	UUM	2	1	4/09/20	0,00795967	825,922	2,1929	2,1506	0,00864198	896,721	2,2706
**Spectrum 3**	UUM	3	1	4/09/20	0,0143457	1138,72	2,1929	2,1506	0,0159889	1269,15	2,2706
**Spectrum 4**	UUM	1	2	5/09/20	0,0212718	1065,14	2,1929	2,1506	0,0210559	1054,33	2,2706
**Spectrum 5**	UUM	2	2	5/09/20	0,0224694	1040,15	2,1929	2,1506	0,0203895	943,862	2,2706
**Spectrum 6**	UUM	3	2	5/09/20	0,0217652	1215,35	2,1929	2,1506	0,0295213	1648,45	2,2706
**Spectrum 7**	UM	1	1	4/09/20	0,0758254	0,799194	2,1929	2,1506	0,0959206	1,011	2,2706
**Spectrum 8**	UM	2	1	4/09/20	0,0760051	0,779882	2,1929	2,1506	0,0982943	1,00859	2,2706
**Spectrum 9**	UM	3	1	4/09/20	0,0731121	0,835428	2,1929	2,1506	0,107553	1,22898	2,2706
**Spectrum 10**	UM	1	2	5/09/20	0,0765947	0,790704	2,1929	2,1506	0,10195	1,05246	2,2706
**Spectrum 11**	UM	2	2	5/09/20	0,0595817	0,755243	2,1929	2,1506	0,086439	1,09568	2,2706
**Spectrum 12**	UM	3	2	5/09/20	0,0630268	0,78846	2,1929	2,1506	0,106736	1,33526	2,2706
**Spectrum 13**	FM	1	2	17/09/20	0,0118848	450,298	2,1929	2,1506	0,0290663	1101,28	2,2706
**Spectrum 14**	FM	1	1	17/09/20	0,0167912	560,553	2,1929	2,1506	0,0387664	1294,17	2,2706
**Spectrum 15**	FM	2	1	17/09/20	0,0108223	389,09	2,1929	2,1506	0,0238278	856,669	2,2706
**Spectrum 16**	FM	2	2	17/09/20	0,0113168	421,494	2,1929	2,1506	0,0297283	1107,23	2,2706
**Spectrum 17**	FM	3	1	17/09/20	0,0192851	581,916	2,1929	2,1506	0,0567032	1710,99	2,2706
**Spectrum 18**	FM	3	2	17/09/20	0,0167581	509,531	2,1929	2,1506	0,049384	1501,52	2,2706
**Spectrum 19**	UM	0	3	20/12/19	0,0738258	2113,61	2,1929	2,1506	0,091985	2633,5	2,2706
**Spectrum 20**	UM	0	2	20/12/19	0,0911003	3101,67	2,1929	2,1506	0,107753	3668,64	2,2706
**Spectrum 21**	UM	0	1	20/12/19	0,067862	2332,85	2,1929	2,1506	0,0834186	2867,63	2,2706
**Spectrum 22**	UUM	0	3	27/12/19	0,00428034	1482,52	2,1929	2,1506	0,00544645	1886,4	2,2706
**Spectrum 23**	UUM	0	2	27/12/19	0,00503642	1436,29	2,1929	2,1506	0,0056208	1602,94	2,2706
**Spectrum 24**	UUM	0	1	27/12/19	0,00485473	1662,33	2,1929	2,1506	0,0055171	1889,13	2,2706
**Spectrum 25**	FM	0	3	4/02/20	0,0133933	545,221	2,1929	2,1506	0,0469844	1912,67	2,2706
**Spectrum 26**	FM	0	2	28/12/19	0,0180334	870,028	2,1929	2,1506	0,0392331	1892,81	2,2706
**Spectrum 27**	FM	0	1	28/12/19	0,0296956	1358,63	2,1929	2,1506	0,0685663	3137,05	2,2706

	Method	Patient	Replicated	Date							
**Spectrum 1**	UUM	1	1	4/09/20	2,258	0,00884204	511,465	2,2989	2,271	0,00687091	397,446
**Spectrum 2**	UUM	2	1	4/09/20	2,258	0,00411994	427,499	2,2989	2,271	0,00277934	288,394
**Spectrum 3**	UUM	3	1	4/09/20	2,258	0,00884389	702	2,2989	2,271	0,00303008	240,518
**Spectrum 4**	UUM	1	2	5/09/20	2,258	0,00854938	428,091	2,2989	2,271	0,00648505	324,724
**Spectrum 5**	UUM	2	2	5/09/20	2,258	0,00962478	445,547	2,2989	2,271	0,00530756	245,696
**Spectrum 6**	UUM	3	2	5/09/20	2,258	0,0109945	613,921	2,2989	2,271	0,00436749	243,877
**Spectrum 7**	UM	1	1	4/09/20	2,258	0,0530235	0,558863	2,2989	2,271	0,119762	1,26229
**Spectrum 8**	UM	2	1	4/09/20	2,258	0,0516839	0,530324	2,2989	2,271	0,124957	1,28217
**Spectrum 9**	UM	3	1	4/09/20	2,258	0,0531953	0,607845	2,2989	2,271	0,0997634	1,13996
**Spectrum 10**	UM	1	2	5/09/20	2,258	0,0537968	0,555356	2,2989	2,271	0,123433	1,27423
**Spectrum 11**	UM	2	2	5/09/20	2,258	0,0430935	0,546242	2,2989	2,271	0,0990223	1,25518
**Spectrum 12**	UM	3	2	5/09/20	2,258	0,050583	0,63279	2,2989	2,271	0,101985	1,27582
**Spectrum 13**	FM	1	2	17/09/20	2,258	0,0116782	442,473	2,2989	2,271	0,0172504	653,596
**Spectrum 14**	FM	1	1	17/09/20	2,258	0,0136908	457,052	2,2989	2,271	0,0251004	837,945
**Spectrum 15**	FM	2	1	17/09/20	2,258	0,00873397	314,008	2,2989	2,271	0,0183681	660,381
**Spectrum 16**	FM	2	2	17/09/20	2,258	0,010568	393,605	2,2989	2,271	0,0157073	585,022
**Spectrum 17**	FM	3	1	17/09/20	2,258	0,0212052	639,855	2,2989	2,271	0,0187499	565,769
**Spectrum 18**	FM	3	2	17/09/20	2,258	0,0182375	554,512	2,2989	2,271	0,0206992	629,36
**Spectrum 19**	UM	0	3	20/12/19	2,258	0,785121	22477,8	2,2989	2,271	0,161951	4636,61
**Spectrum 20**	UM	0	2	20/12/19	2,258	0,802461	27321,2	2,2989	2,271	0,174457	5939,69
**Spectrum 21**	UM	0	1	20/12/19	2,258	0,680124	23380,2	2,2989	2,271	0,14055	4831,6
**Spectrum 22**	UUM	0	3	27/12/19	2,258	0,0554363	19200,6	2,2989	2,271	0,00972772	3369,24
**Spectrum 23**	UUM	0	2	27/12/19	2,258	0,0688061	19622,2	2,2989	2,271	0,00887945	2532,25
**Spectrum 24**	UUM	0	1	27/12/19	2,258	0,0626902	21466	2,2989	2,271	0,00735094	2517,07
**Spectrum 25**	FM	0	3	4/02/20	2,258	0,573507	23346,6	2,2989	2,271	0,0493721	2009,86
**Spectrum 26**	FM	0	2	28/12/19	2,258	0,423084	20411,8	2,2989	2,271	0,0810104	3908,37
**Spectrum 27**	FM	0	1	28/12/19	2,258	0,623232	28514,2	2,2989	2,271	0,143029	6543,85

	Method	Patient	Replicated	Date							
**Spectrum 1**	UUM	1	1	4/09/20	2,3723	2,3652	0,00376599	217,843	2,3801	2,3724	0,0085138
**Spectrum 2**	UUM	2	1	4/09/20	2,3723	2,3652	0,00134892	139,969	2,3801	2,3724	0,00328336
**Spectrum 3**	UUM	3	1	4/09/20	2,3723	2,3652	0,00387764	307,794	2,3801	2,3724	0,00656738
**Spectrum 4**	UUM	1	2	5/09/20	2,3723	2,3652	0,00396778	198,678	2,3801	2,3724	0,00737694
**Spectrum 5**	UUM	2	2	5/09/20	2,3723	2,3652	0,00424559	196,536	2,3801	2,3724	0,00529212
**Spectrum 6**	UUM	3	2	5/09/20	2,3723	2,3652	0,00590652	329,815	2,3801	2,3724	0,010887
**Spectrum 7**	UM	1	1	4/09/20	2,3723	2,3652	0,0244167	0,25735	2,3801	2,3724	0,0265606
**Spectrum 8**	UM	2	1	4/09/20	2,3723	2,3652	0,0233092	0,239174	2,3801	2,3724	0,0250083
**Spectrum 9**	UM	3	1	4/09/20	2,3723	2,3652	0,0239267	0,273403	2,3801	2,3724	0,0301481
**Spectrum 10**	UM	1	2	5/09/20	2,3723	2,3652	0,023009	0,237527	2,3801	2,3724	0,0263607
**Spectrum 11**	UM	2	2	5/09/20	2,3723	2,3652	0,0180625	0,228955	2,3801	2,3724	0,0216491
**Spectrum 12**	UM	3	2	5/09/20	2,3723	2,3652	0,0235153	0,294175	2,3801	2,3724	0,0284447
**Spectrum 13**	FM	1	2	17/09/20	2,3723	2,3652	0,00452783	171,554	2,3801	2,3724	0,0073355
**Spectrum 14**	FM	1	1	17/09/20	2,3723	2,3652	0,00626893	209,281	2,3801	2,3724	0,00990897
**Spectrum 15**	FM	2	1	17/09/20	2,3723	2,3652	0,00402816	144,823	2,3801	2,3724	0,0060514
**Spectrum 16**	FM	2	2	17/09/20	2,3723	2,3652	0,00393782	146,664	2,3801	2,3724	0,00719479
**Spectrum 17**	FM	3	1	17/09/20	2,3723	2,3652	0,00825208	249,002	2,3801	2,3724	0,0147485
**Spectrum 18**	FM	3	2	17/09/20	2,3723	2,3652	0,00794393	241,535	2,3801	2,3724	0,0121066
**Spectrum 19**	UM	0	3	20/12/19	2,3723	2,3652	0,0521762	1493,79	2,3801	2,3724	0,0431556
**Spectrum 20**	UM	0	2	20/12/19	2,3723	2,3652	0,0610472	2078,46	2,3801	2,3724	0,0453065
**Spectrum 21**	UM	0	1	20/12/19	2,3723	2,3652	0,0467714	1607,83	2,3801	2,3724	0,0391131
**Spectrum 22**	UUM	0	3	27/12/19	2,3723	2,3652	0,00276056	956,133	2,3801	2,3724	0,00181748
**Spectrum 23**	UUM	0	2	27/12/19	2,3723	2,3652	0,00290263	827,775	2,3801	2,3724	0,00195128
**Spectrum 24**	UUM	0	1	27/12/19	2,3723	2,3652	0,00262817	899,922	2,3801	2,3724	0,00188146
**Spectrum 25**	FM	0	3	4/02/20	2,3723	2,3652	0,0287438	1170,12	2,3801	2,3724	0,0127583
**Spectrum 26**	FM	0	2	28/12/19	2,3723	2,3652	0,0215435	1039,37	2,3801	2,3724	0,0140657
**Spectrum 27**	FM	0	1	28/12/19	2,3723	2,3652	0,0290663	1329,84	2,3801	2,3724	0,0214999

	Method	Patient	Replicated	Date							
**Spectrum 1**	UUM	1	1	4/09/20	492,478	3,0261	3,0147	0,0164246	950,076	3,0391	3,0263
**Spectrum 2**	UUM	2	1	4/09/20	340,692	3,0261	3,0147	0,00531161	551,151	3,0391	3,0263
**Spectrum 3**	UUM	3	1	4/09/20	521,298	3,0261	3,0147	0,00529269	420,117	3,0391	3,0263
**Spectrum 4**	UUM	1	2	5/09/20	369,384	3,0261	3,0147	0,0179331	897,961	3,0391	3,0263
**Spectrum 5**	UUM	2	2	5/09/20	244,981	3,0261	3,0147	0,013073	605,171	3,0391	3,0263
**Spectrum 6**	UUM	3	2	5/09/20	607,919	3,0261	3,0147	0,00615105	343,469	3,0391	3,0263
**Spectrum 7**	UM	1	1	4/09/20	0,279947	3,0261	3,0147	0,103275	1,08851	3,0391	3,0263
**Spectrum 8**	UM	2	1	4/09/20	0,256609	3,0261	3,0147	0,111424	1,14332	3,0391	3,0263
**Spectrum 9**	UM	3	1	4/09/20	0,344493	3,0261	3,0147	0,0702368	0,802574	3,0391	3,0263
**Spectrum 10**	UM	1	2	5/09/20	0,272127	3,0261	3,0147	0,107665	1,11145	3,0391	3,0263
**Spectrum 11**	UM	2	2	5/09/20	0,274419	3,0261	3,0147	0,0862465	1,09324	3,0391	3,0263
**Spectrum 12**	UM	3	2	5/09/20	0,355842	3,0261	3,0147	0,0789877	0,98813	3,0391	3,0263
**Spectrum 13**	FM	1	2	17/09/20	277,933	3,0261	3,0147	0,00990673	375,353	3,0391	3,0263
**Spectrum 14**	FM	1	1	17/09/20	330,799	3,0261	3,0147	0,0091711	306,166	3,0391	3,0263
**Spectrum 15**	FM	2	1	17/09/20	217,563	3,0261	3,0147	0,00403792	145,174	3,0391	3,0263
**Spectrum 16**	FM	2	2	17/09/20	267,971	3,0261	3,0147	0,0107013	398,572	3,0391	3,0263
**Spectrum 17**	FM	3	1	17/09/20	445,027	3,0261	3,0147	0,00982554	296,48	3,0391	3,0263
**Spectrum 18**	FM	3	2	17/09/20	368,101	3,0261	3,0147	0,00995194	302,589	3,0391	3,0263
**Spectrum 19**	UM	0	3	20/12/19	1235,53	3,0261	3,0147	0,081998	2347,58	3,0391	3,0263
**Spectrum 20**	UM	0	2	20/12/19	1542,54	3,0261	3,0147	0,0939926	3200,14	3,0391	3,0263
**Spectrum 21**	UM	0	1	20/12/19	1344,56	3,0261	3,0147	0,0757156	2602,83	3,0391	3,0263
**Spectrum 22**	UUM	0	3	27/12/19	629,493	3,0261	3,0147	0,00313904	1087,22	3,0391	3,0263
**Spectrum 23**	UUM	0	2	27/12/19	556,466	3,0261	3,0147	0,0034955	996,848	3,0391	3,0263
**Spectrum 24**	UUM	0	1	27/12/19	644,238	3,0261	3,0147	0,00360748	1235,25	3,0391	3,0263
**Spectrum 25**	FM	0	3	4/02/20	519,372	3,0261	3,0147	0,0076567	311,693	3,0391	3,0263
**Spectrum 26**	FM	0	2	28/12/19	678,602	3,0261	3,0147	0,0143521	692,423	3,0391	3,0263
**Spectrum 27**	FM	0	1	28/12/19	983,664	3,0261	3,0147	0,0291119	1331,93	3,0391	3,0263

	Method	Patient	Replicated	Date							
**Spectrum 1**	UUM	1	1	4/09/20	0,0218797	1265,63	3,0539	3,0392	0,0146091	845,061	3,4458
**Spectrum 2**	UUM	2	1	4/09/20	0,00589372	611,553	3,0539	3,0392	0,00455078	472,205	3,4458
**Spectrum 3**	UUM	3	1	4/09/20	0,00729808	579,299	3,0539	3,0392	0,00532509	422,689	3,4458
**Spectrum 4**	UUM	1	2	5/09/20	0,0237847	1190,97	3,0539	3,0392	0,0150477	753,482	3,4458
**Spectrum 5**	UUM	2	2	5/09/20	0,0182889	846,625	3,0539	3,0392	0,0111777	517,437	3,4458
**Spectrum 6**	UUM	3	2	5/09/20	0,00849712	474,472	3,0539	3,0392	0,00762578	425,817	3,4458
**Spectrum 7**	UM	1	1	4/09/20	0,106486	1,12235	3,0539	3,0392	0,0649668	0,684745	3,4458
**Spectrum 8**	UM	2	1	4/09/20	0,10689	1,09679	3,0539	3,0392	0,0652368	0,669389	3,4458
**Spectrum 9**	UM	3	1	4/09/20	0,0649082	0,741685	3,0539	3,0392	0,0453825	0,518572	3,4458
**Spectrum 10**	UM	1	2	5/09/20	0,104288	1,07659	3,0539	3,0392	0,0640307	0,661003	3,4458
**Spectrum 11**	UM	2	2	5/09/20	0,0789339	1,00055	3,0539	3,0392	0,0498989	0,632506	3,4458
**Spectrum 12**	UM	3	2	5/09/20	0,0613007	0,766867	3,0539	3,0392	0,0398589	0,498631	3,4458
**Spectrum 13**	FM	1	2	17/09/20	0,0045193	171,23	3,0539	3,0392	0,00293781	111,31	3,4458
**Spectrum 14**	FM	1	1	17/09/20	0,00628072	209,674	3,0539	3,0392	0,00454588	151,759	3,4458
**Spectrum 15**	FM	2	1	17/09/20	0,00151818	54,5825	3,0539	3,0392	0,00207145	74,4739	3,4458
**Spectrum 16**	FM	2	2	17/09/20	0,00491755	183,155	3,0539	3,0392	0,00373494	139,108	3,4458
**Spectrum 17**	FM	3	1	17/09/20	0,00418023	126,136	3,0539	3,0392	0,00375513	113,309	3,4458
**Spectrum 18**	FM	3	2	17/09/20	0,00394509	119,951	3,0539	3,0392	0,00375341	114,123	3,4458
**Spectrum 19**	UM	0	3	20/12/19	0,0895191	2562,9	3,0539	3,0392	0,0517468	1481,5	3,4458
**Spectrum 20**	UM	0	2	20/12/19	0,110978	3778,43	3,0539	3,0392	0,0695595	2368,27	3,4458
**Spectrum 21**	UM	0	1	20/12/19	0,0826601	2841,56	3,0539	3,0392	0,0503626	1731,28	3,4458
**Spectrum 22**	UUM	0	3	27/12/19	0,00460081	1593,51	3,0539	3,0392	0,00336646	1165,99	3,4458
**Spectrum 23**	UUM	0	2	27/12/19	0,00513871	1465,46	3,0539	3,0392	0,00353357	1007,71	3,4458
**Spectrum 24**	UUM	0	1	27/12/19	0,00485538	1662,55	3,0539	3,0392	0,00332121	1137,23	3,4458
**Spectrum 25**	FM	0	3	4/02/20	0,00482334	196,351	3,0539	3,0392	0,00639797	260,452	3,4458
**Spectrum 26**	FM	0	2	28/12/19	0,0130744	630,778	3,0539	3,0392	0,0090836	438,241	3,4458
**Spectrum 27**	FM	0	1	28/12/19	0,0244277	1117,62	3,0539	3,0392	0,0186012	851,045	3,4458

	Method	Patient	Replicated	Date							
**Spectrum 1**	UUM	1	1	4/09/20	3,4102	0,127826	7394,06	3,5685	3,5438	0,800896	46327,6
**Spectrum 2**	UUM	2	1	4/09/20	3,4102	0,0591161	6134,08	3,5685	3,5438	0,606661	62949,2
**Spectrum 3**	UUM	3	1	4/09/20	3,4102	0,133557	10601,4	3,5685	3,5438	0,802075	63666,2
**Spectrum 4**	UUM	1	2	5/09/20	3,4102	0,138615	6940,82	3,5685	3,5438	0,539372	27007,8
**Spectrum 5**	UUM	2	2	5/09/20	3,4102	0,134348	6219,22	3,5685	3,5438	0,445231	20610,5
**Spectrum 6**	UUM	3	2	5/09/20	3,4102	0,198777	11099,5	3,5685	3,5438	0,621995	34731,7
**Spectrum 7**	UM	1	1	4/09/20	3,4102	0,193934	2,04405	3,5685	3,5438	0,083923	0,884541
**Spectrum 8**	UM	2	1	4/09/20	3,4102	0,170554	1,75004	3,5685	3,5438	0,081103	0,832191
**Spectrum 9**	UM	3	1	4/09/20	3,4102	0,26614	3,0411	3,5685	3,5438	0,12085	1,38091
**Spectrum 10**	UM	1	2	5/09/20	3,4102	0,197372	2,03752	3,5685	3,5438	0,0717928	0,741133
**Spectrum 11**	UM	2	2	5/09/20	3,4102	0,153683	1,94805	3,5685	3,5438	0,0590249	0,748185
**Spectrum 12**	UM	3	2	5/09/20	3,4102	0,247442	3,09548	3,5685	3,5438	0,078143	0,977563
**Spectrum 13**	FM	1	2	17/09/20	3,4102	0,13648	5171,05	3,5685	3,5438	0,0310308	1175,71
**Spectrum 14**	FM	1	1	17/09/20	3,4102	0,162212	5415,27	3,5685	3,5438	0,0367373	1226,43
**Spectrum 15**	FM	2	1	17/09/20	3,4102	0,117759	4233,72	3,5685	3,5438	0,0310388	1115,92
**Spectrum 16**	FM	2	2	17/09/20	3,4102	0,120845	4500,88	3,5685	3,5438	0,0331984	1236,48
**Spectrum 17**	FM	3	1	17/09/20	3,4102	0,286732	8651,99	3,5685	3,5438	0,0527757	1592,48
**Spectrum 18**	FM	3	2	17/09/20	3,4102	0,239644	7286,39	3,5685	3,5438	0,0523419	1591,46
**Spectrum 19**	UM	0	3	20/12/19	3,4102	0,134569	3852,68	3,5685	3,5438	0,0815312	2334,21
**Spectrum 20**	UM	0	2	20/12/19	3,4102	0,169643	5775,8	3,5685	3,5438	0,0860282	2928,98
**Spectrum 21**	UM	0	1	20/12/19	3,4102	0,133451	4587,55	3,5685	3,5438	0,0616728	2120,09
**Spectrum 22**	UUM	0	3	27/12/19	3,4102	0,013546	4691,71	3,5685	3,5438	0,0296592	10272,6
**Spectrum 23**	UUM	0	2	27/12/19	3,4102	0,015791	4503,3	3,5685	3,5438	0,0275929	7868,97
**Spectrum 24**	UUM	0	1	27/12/19	3,4102	0,0148042	5069,17	3,5685	3,5438	0,0188811	6465,17
**Spectrum 25**	FM	0	3	4/02/20	3,4102	0,11982	4877,71	3,5685	3,5438	0,0275836	1122,89
**Spectrum 26**	FM	0	2	28/12/19	3,4102	0,0838432	4045,04	3,5685	3,5438	0,027519	1327,66
**Spectrum 27**	FM	0	1	28/12/19	3,4102	0,14684	6718,21	3,5685	3,5438	0,036537	1671,64

	Method	Patient	Replicated	Date							
**Spectrum 1**	UUM	1	1	4/09/20	3,5908	3,5687	1,12165	64881,8	3,5976	3,5909	0,0166094
**Spectrum 2**	UUM	2	1	4/09/20	3,5908	3,5687	0,86421	89673,4	3,5976	3,5909	0,0103908
**Spectrum 3**	UUM	3	1	4/09/20	3,5908	3,5687	1,15279	91505	3,5976	3,5909	0,0173789
**Spectrum 4**	UUM	1	2	5/09/20	3,5908	3,5687	0,770445	38578,3	3,5976	3,5909	0,0233996
**Spectrum 5**	UUM	2	2	5/09/20	3,5908	3,5687	0,609432	28211,6	3,5976	3,5909	0,0134895
**Spectrum 6**	UUM	3	2	5/09/20	3,5908	3,5687	0,821497	45871,7	3,5976	3,5909	0,0130897
**Spectrum 7**	UM	1	1	4/09/20	3,5908	3,5687	0,0357707	0,37702	3,5976	3,5909	0,0204908
**Spectrum 8**	UM	2	1	4/09/20	3,5908	3,5687	0,0376119	0,385932	3,5976	3,5909	0,0194654
**Spectrum 9**	UM	3	1	4/09/20	3,5908	3,5687	0,0859286	0,981879	3,5976	3,5909	0,0167057
**Spectrum 10**	UM	1	2	5/09/20	3,5908	3,5687	0,033566	0,34651	3,5976	3,5909	0,0194692
**Spectrum 11**	UM	2	2	5/09/20	3,5908	3,5687	0,027677	0,350827	3,5976	3,5909	0,0148367
**Spectrum 12**	UM	3	2	5/09/20	3,5908	3,5687	0,0605778	0,757824	3,5976	3,5909	0,0066682
**Spectrum 13**	FM	1	2	17/09/20	3,5908	3,5687	0,0213771	809,952	3,5976	3,5909	0,00379287
**Spectrum 14**	FM	1	1	17/09/20	3,5908	3,5687	0,0274752	917,225	3,5976	3,5909	0,00465071
**Spectrum 15**	FM	2	1	17/09/20	3,5908	3,5687	0,0222909	801,416	3,5976	3,5909	0,00265394
**Spectrum 16**	FM	2	2	17/09/20	3,5908	3,5687	0,021626	805,461	3,5976	3,5909	0,0037279
**Spectrum 17**	FM	3	1	17/09/20	3,5908	3,5687	0,022806	688,159	3,5976	3,5909	0,00214384
**Spectrum 18**	FM	3	2	17/09/20	3,5908	3,5687	0,0271399	825,19	3,5976	3,5909	0,00250373
**Spectrum 19**	UM	0	3	20/12/19	3,5908	3,5687	0,0345314	988,624	3,5976	3,5909	0,0104686
**Spectrum 20**	UM	0	2	20/12/19	3,5908	3,5687	0,0344268	1172,12	3,5976	3,5909	0,0125148
**Spectrum 21**	UM	0	1	20/12/19	3,5908	3,5687	0,0233511	802,727	3,5976	3,5909	0,00888863
**Spectrum 22**	UUM	0	3	27/12/19	3,5908	3,5687	0,0282031	9768,28	3,5976	3,5909	0,00186971
**Spectrum 23**	UUM	0	2	27/12/19	3,5908	3,5687	0,0244398	6969,77	3,5976	3,5909	0,00122338
**Spectrum 24**	UUM	0	1	27/12/19	3,5908	3,5687	0,0123968	4244,83	3,5976	3,5909	0,00090688
**Spectrum 25**	FM	0	3	4/02/20	3,5908	3,5687	0,0113212	460,87	3,5976	3,5909	0,00349932
**Spectrum 26**	FM	0	2	28/12/19	3,5908	3,5687	0,0165915	800,462	3,5976	3,5909	0,00451415
**Spectrum 27**	FM	0	1	28/12/19	3,5908	3,5687	0,0256925	1175,48	3,5976	3,5909	0,00770318

	Method	Patient	Replicated	Date							
**Spectrum 1**	UUM	1	1	4/09/20	960,764	3,6888	3,664	1,20932	69952,7	3,664	3,6399
**Spectrum 2**	UUM	2	1	4/09/20	1078,19	3,6888	3,664	0,841447	87311,4	3,664	3,6399
**Spectrum 3**	UUM	3	1	4/09/20	1379,49	3,6888	3,664	1,24158	98553,1	3,664	3,6399
**Spectrum 4**	UUM	1	2	5/09/20	1171,68	3,6888	3,664	1,03347	51748,9	3,664	3,6399
**Spectrum 5**	UUM	2	2	5/09/20	624,452	3,6888	3,664	0,944163	43706,9	3,664	3,6399
**Spectrum 6**	UUM	3	2	5/09/20	730,916	3,6888	3,664	0,858341	47929	3,664	3,6399
**Spectrum 7**	UM	1	1	4/09/20	0,215971	3,6888	3,664	0,105078	1,10752	3,664	3,6399
**Spectrum 8**	UM	2	1	4/09/20	0,199732	3,6888	3,664	0,102994	1,05682	3,664	3,6399
**Spectrum 9**	UM	3	1	4/09/20	0,190891	3,6888	3,664	0,125753	1,43694	3,664	3,6399
**Spectrum 10**	UM	1	2	5/09/20	0,200985	3,6888	3,664	0,0801192	0,827088	3,664	3,6399
**Spectrum 11**	UM	2	2	5/09/20	0,188066	3,6888	3,664	0,0832461	1,05521	3,664	3,6399
**Spectrum 12**	UM	3	2	5/09/20	0,0834187	3,6888	3,664	0,0943336	1,18011	3,664	3,6399
**Spectrum 13**	FM	1	2	17/09/20	143,707	3,6888	3,664	0,0304861	1155,08	3,664	3,6399
**Spectrum 14**	FM	1	1	17/09/20	155,258	3,6888	3,664	0,030651	1023,25	3,664	3,6399
**Spectrum 15**	FM	2	1	17/09/20	95,4158	3,6888	3,664	0,0320268	1151,45	3,664	3,6399
**Spectrum 16**	FM	2	2	17/09/20	138,846	3,6888	3,664	0,0293735	1094,02	3,664	3,6399
**Spectrum 17**	FM	3	1	17/09/20	64,6893	3,6888	3,664	0,0256932	775,277	3,664	3,6399
**Spectrum 18**	FM	3	2	17/09/20	76,1258	3,6888	3,664	0,0359906	1094,3	3,664	3,6399
**Spectrum 19**	UM	0	3	20/12/19	299,713	3,6888	3,664	0,106912	3060,85	3,664	3,6399
**Spectrum 20**	UM	0	2	20/12/19	426,087	3,6888	3,664	0,119199	4058,33	3,664	3,6399
**Spectrum 21**	UM	0	1	20/12/19	305,559	3,6888	3,664	0,0983234	3380	3,664	3,6399
**Spectrum 22**	UUM	0	3	27/12/19	647,584	3,6888	3,664	1,05427	365151	3,664	3,6399
**Spectrum 23**	UUM	0	2	27/12/19	348,884	3,6888	3,664	1,07288	305966	3,664	3,6399
**Spectrum 24**	UUM	0	1	27/12/19	310,528	3,6888	3,664	0,96957	331995	3,664	3,6399
**Spectrum 25**	FM	0	3	4/02/20	142,452	3,6888	3,664	0,0692828	2820,4	3,664	3,6399
**Spectrum 26**	FM	0	2	28/12/19	217,787	3,6888	3,664	0,0549263	2649,94	3,664	3,6399
**Spectrum 27**	FM	0	1	28/12/19	352,436	3,6888	3,664	0,0859095	3930,54	3,664	3,6399

	Method	Patient	Replicated	Date								
**Spectrum 1**	UUM	1	1	4/09/20	1,6744	96855,3	9,5201	9,5	-5,62E-05	-3,25352	6,4595
**Spectrum 2**	UUM	2	1	4/09/20	1,1967	124173	9,5201	9,5	0,00035838	37,1868	6,4595
**Spectrum 3**	UUM	3	1	4/09/20	1,67438	132907	9,5201	9,5	5,79E-05	4,59314	6,4595
**Spectrum 4**	UUM	1	2	5/09/20	1,31449	65820,3	9,5201	9,5	-0,0011375	-56,9596	6,4595
**Spectrum 5**	UUM	2	2	5/09/20	1,25534	58111,9	9,5201	9,5	0,00050643	23,4433	6,4595
**Spectrum 6**	UUM	3	2	5/09/20	1,23499	68960,7	9,5201	9,5	0,00074074	41,3624	6,4595
**Spectrum 7**	UM	1	1	4/09/20	0,0935184	0,985676	9,5201	9,5	-0,0021737	-0,0229111	6,4595
**Spectrum 8**	UM	2	1	4/09/20	0,089467	0,918013	9,5201	9,5	-1,70E-07	-1,75E-06	6,4595
**Spectrum 9**	UM	3	1	4/09/20	0,130662	1,49303	9,5201	9,5	-0,0015542	-0,0177592	6,4595
**Spectrum 10**	UM	1	2	5/09/20	0,0674415	0,696214	9,5201	9,5	-0,000409	-0,0042223	6,4595
**Spectrum 11**	UM	2	2	5/09/20	0,075837	0,96129	9,5201	9,5	0,0003003	0,0038065	6,4595
**Spectrum 12**	UM	3	2	5/09/20	0,106822	1,33634	9,5201	9,5	-0,0014519	-0,0181629	6,4595
**Spectrum 13**	FM	1	2	17/09/20	0,0306395	1160,89	9,5201	9,5	-0,0020318	-76,9812	6,4595
**Spectrum 14**	FM	1	1	17/09/20	0,0326899	1091,31	9,5201	9,5	-0,0037893	-126,501	6,4595
**Spectrum 15**	FM	2	1	17/09/20	0,0330446	1188,04	9,5201	9,5	-0,0028115	-101,08	6,4595
**Spectrum 16**	FM	2	2	17/09/20	0,0281883	1049,88	9,5201	9,5	-0,0037469	-139,555	6,4595
**Spectrum 17**	FM	3	1	17/09/20	0,0290495	876,551	9,5201	9,5	-0,0015354	-46,3294	6,4595
**Spectrum 18**	FM	3	2	17/09/20	0,0351232	1067,92	9,5201	9,5	-0,0025728	-78,2272	6,4595
**Spectrum 19**	UM	0	3	20/12/19	0,0771652	2209,22	9,5201	9,5	-0,0025447	-72,8542	6,4595
**Spectrum 20**	UM	0	2	20/12/19	0,0799135	2720,79	9,5201	9,5	-0,0022198	-75,5779	6,4595
**Spectrum 21**	UM	0	1	20/12/19	0,0668485	2298,01	9,5201	9,5	-0,0019852	-68,2432	6,4595
**Spectrum 22**	UUM	0	3	27/12/19	0,9727	336899	9,5201	9,5	0,00016672	57,7455	6,4595
**Spectrum 23**	UUM	0	2	27/12/19	1,04475	297942	9,5201	9,5	-0,00014	-39,9208	6,4595
**Spectrum 24**	UUM	0	1	27/12/19	0,972278	332922	9,5201	9,5	-3,78E-05	-12,9426	6,4595
**Spectrum 25**	FM	0	3	4/02/20	0,0583374	2374,83	9,5201	9,5	-0,0007039	-28,6536	6,4595
**Spectrum 26**	FM	0	2	28/12/19	0,0428664	2068,1	9,5201	9,5	-0,0003224	-15,5548	6,4595
**Spectrum 27**	FM	0	1	28/12/19	0,0697508	3191,24	9,5201	9,5	-0,0011714	-53,5945	6,4595

	Method	Patient	Replicated	Date							
**Spectrum 1**	UUM	1	1	4/09/20	6,4394	-0,001537	-88,9098	0,5401	0,52	-0,0009155	-52,9563
**Spectrum 2**	UUM	2	1	4/09/20	6,4394	-0,0004736	-49,1401	0,5401	0,52	-0,0008139	-84,4521
**Spectrum 3**	UUM	3	1	4/09/20	6,4394	0,00025963	20,6085	0,5401	0,52	-0,0001036	-8,22669
**Spectrum 4**	UUM	1	2	5/09/20	6,4394	0,00043865	21,9646	0,5401	0,52	-0,0006364	-31,8639
**Spectrum 5**	UUM	2	2	5/09/20	6,4394	-0,0009879	-45,731	0,5401	0,52	-0,0020474	-94,7763
**Spectrum 6**	UUM	3	2	5/09/20	6,4394	0,00112658	62,9071	0,5401	0,52	-0,0002065	-11,5281
**Spectrum 7**	UM	1	1	4/09/20	6,4394	-0,0007898	-0,0083241	0,5401	0,52	0,00677918	0,071452
**Spectrum 8**	UM	2	1	4/09/20	6,4394	-0,0011308	-0,0116026	0,5401	0,52	0,00750632	0,0770217
**Spectrum 9**	UM	3	1	4/09/20	6,4394	-0,0005351	-0,006115	0,5401	0,52	0,00765265	0,0874444
**Spectrum 10**	UM	1	2	5/09/20	6,4394	0,00106516	0,0109959	0,5401	0,52	0,00781241	0,0806492
**Spectrum 11**	UM	2	2	5/09/20	6,4394	-0,0002869	-0,0036366	0,5401	0,52	0,0069746	0,0884082
**Spectrum 12**	UM	3	2	5/09/20	6,4394	-0,0040413	-0,0505568	0,5401	0,52	0,00311536	0,0389729
**Spectrum 13**	FM	1	2	17/09/20	6,4394	-0,0033943	-128,606	0,5401	0,52	-0,0002433	-9,21837
**Spectrum 14**	FM	1	1	17/09/20	6,4394	-0,0091231	-304,563	0,5401	0,52	0,00064232	21,4432
**Spectrum 15**	FM	2	1	17/09/20	6,4394	-0,0009985	-35,9002	0,5401	0,52	-0,0009792	-35,2059
**Spectrum 16**	FM	2	2	17/09/20	6,4394	-0,0055824	-207,917	0,5401	0,52	0,00211074	78,6146
**Spectrum 17**	FM	3	1	17/09/20	6,4394	-0,0021068	-63,5722	0,5401	0,52	-0,001286	-38,8046
**Spectrum 18**	FM	3	2	17/09/20	6,4394	-0,0040877	-124,285	0,5401	0,52	-9,75E-05	-2,96518
**Spectrum 19**	UM	0	3	20/12/19	6,4394	-0,00531	-152,023	0,5401	0,52	-0,0021878	-62,6346
**Spectrum 20**	UM	0	2	20/12/19	6,4394	-0,0042818	-145,781	0,5401	0,52	1,04E-05	0,352936
**Spectrum 21**	UM	0	1	20/12/19	6,4394	-0,0042738	-146,919	0,5401	0,52	-0,0015968	-54,8924
**Spectrum 22**	UUM	0	3	27/12/19	6,4394	4,62E-06	1,59995	0,5401	0,52	0,00020726	71,7837
**Spectrum 23**	UUM	0	2	27/12/19	6,4394	-0,0002843	-81,072	0,5401	0,52	-3,08E-05	-8,7877
**Spectrum 24**	UUM	0	1	27/12/19	6,4394	-0,000251	-85,9564	0,5401	0,52	-0,0003067	-105,019
**Spectrum 25**	FM	0	3	4/02/20	6,4394	-0,0032312	-131,537	0,5401	0,52	-0,0021681	-88,2579
**Spectrum 26**	FM	0	2	28/12/19	6,4394	-0,0011202	-54,0437	0,5401	0,52	-0,0032595	-157,253
**Spectrum 27**	FM	0	1	28/12/19	6,4394	-0,0044517	-203,675	0,5401	0,52	-0,0036761	-168,191
