# Supplementary material for: Development of an optimized method for processing peripheral blood mononuclear cells for 1H-nuclear magnetic resonance-based metabolomic profiling
Source: PLoS One. 2021 Feb 25;16(2):e0247668. doi: 10.1371/journal.pone.0247668 (PMC7906414; doi:10.1371/journal.pone.0247668)
Supplement: S2 Table — (PDF) [file pone.0247668.s002.pdf]

Type	H's	Metabolite	ppm 1	ppm 2	Spectrum 1	Spectrum 2
s		TSP	0,0428	-0,0466	215,9721	249,4394
		Noise	0,5401	0,52	-0,2952	-0,5353
t	3	2-hydroxybutyrate	0,9229	0,8599	9,2291	8,1685
-	-	Unknown 1	0,9354	0,9247	1,8169	1,2306
t	3	Isoleucine	0,9479	0,9359	3,6527	2,7672
t	6	Leucine	0,9799	0,9503	21,9572	18,2462
d	3	Valine	1,0016	0,9799	7,8503	5,8723
d	3	Isoleucine	1,0248	1,0016	4,5176	3,4162
d	3	Valine	1,058	1,0337	7,3533	5,7269
-	-	Unknown 2	1,1007	1,0649	4,4600	4,3521
t	3	Ethanol	1,2478	1,1354	575,3369	679,6375
s	6	3-Hidroxyisovalerate	1,2576	1,2508	1,0407	1,0422
-	-	Unknown 3	1,3109	1,2737	9,4471	8,9682
d	3	Lactate	1,3503	1,3136	60,5807	39,2258
d	3	Alanine	1,5001	1,4645	29,7108	19,1974
m	2	2-hydroxybutyrate	1,6294	1,5908	17,8739	20,4924
m	2	2-hydroxybutyrate	1,6653	1,6299	18,9516	21,0903
m	2	Lysine	1,7974	1,6768	55,4361	48,1154
-	-	Unknown 4	1,8234	1,8096	1,8096	1,5047
-	-	Unknown 5	1,8582	1,8434	1,8418	1,1523
m	2	Lysine	1,9177	1,8747	13,6392	8,4106
s	3	Acetate	1,9285	1,9179	13,0994	11,0674
m	2	Glutamate	2,098	1,996	17,7470	13,0238
m	2	Glutamine	2,1356	2,1114	5,3577	4,7193
s	3	Hidroxyacetone	2,1498	2,1359	6,0266	5,2348
m	5	Methionine	2,1929	2,1506	6,2602	5,6836
s	-	Unknown 6	2,2326	2,2234	2,0663	3,0105
s	-	Unknown 7	2,2398	2,233	2,6135	2,9711
s	3	Acetoacetate	2,2706	2,258	2,8512	2,7096
m	1	Valine	2,2989	2,271	2,2156	1,8279
s	3	Methylacetoacetate	2,34	2,3302	0,8391	0,5541
-	-	Unknown 8	2,3522	2,3404	2,5302	1,7795
m	2	Glutamate	2,3651	2,3523	3,6605	2,5232
s	4	Succinate	2,3723	2,3652	1,2144	0,8871
s	3	Pyruvate	2,3801	2,3724	2,7453	2,1594
-	-	Unknown 9	2,4117	2,4035	1,2358	0,7328
-	-	Unknown 10	2,4674	2,4335	3,5089	2,0007
m	2	Glutamine	2,4983	2,4674	15,2572	17,7272
m	2	GSH+GSSG	2,5612	2,5522	1,2574	1,0933
d	2	Citrate	2,5952	2,5224	5,6013	4,7138
d	2	Citrate	2,6434	2,6285	0,8488	0,3532
t	2	Methionine	2,663	2,6435	3,4633	2,9886
-	-	Unknown 11	2,673	2,6631	1,2499	1,3365
m	1	Aspartate	2,7038	2,6726	3,1151	3,5618

s	3	Sarcosine	2,7196	2,7089	0,1582	-0,0392
-	-	Unknown 12	2,7377	2,7293	0,3007	-0,1275
m	1	Aspartate	2,8541	2,7938	5,0548	4,6317
s	9	Trimethylamine	2,8792	2,8591	1,1606	0,9486
dd	2	GSH+GSSG	2,9859	2,9316	2,4505	2,2987
-	-	Unknown 13	3,0261	3,0147	5,2962	3,4933
s	3	Creatine	3,0391	3,0263	7,0553	3,8761
s	3	Creatinine	3,0539	3,0392	4,7108	2,9929
s	2	Malonate	3,1376	3,1285	1,6886	1,5158
m	1	Phenylalanine	3,1671	3,1378	3,5260	2,3887
s	9	Choline	3,2082	3,1984	6,8119	5,1387
s	9	O-Phosphocoline	3,2143	3,2082	1,2067	0,7291
s	9	Trimethylamine N-oxide	3,2245	3,2146	10,0227	10,6728
s	9	Carnitine	3,2343	3,2247	10,9563	11,9505
s	9	Betaine	3,2416	3,2342	5,4479	5,2176
t	2	Taurine	3,2803	3,2419	64,1011	63,4079
m	1	Phenylalanine	3,3122	3,2904	3,6302	1,9871
s	3	Methanol	3,377	3,3531	28,8353	32,0928
t	2	Taurine	3,4458	3,4102	41,2185	38,8789
-	-	Unknown 14	3,5415	3,5165	10,2164	10,2472
m	4	Glycerol	3,5685	3,5438	258,2550	398,9838
s	2	Glycine	3,5908	3,5687	361,6860	568,3668
d	1	Treonine	3,5976	3,5909	5,3558	6,8338
d	1	Valine	3,6236	3,6086	7,0417	7,3106
-	-	Unknown 15	3,6347	3,6244	5,9282	7,1155
m	4	Glycerol	3,664	3,6399	539,9235	787,0317
q	2	Ethanol	3,6888	3,664	389,9540	553,3960
-	-	Unknown 16	3,7264	3,7027	101,7559	151,0276
-	-	Unknown 17	3,7405	3,7346	5,5964	7,6214
m	1	Glutamate	3,7671	3,7415	24,4936	28,0235
dd + q	1	Serine + Alanine	3,818	3,7665	345,6241	531,8696
dd	1	Methionine	3,8593	3,8349	8,2265	8,7808
s	2	Betaine	3,8892	3,8839	1,2329	1,0459
-	-	Unknown 18	3,9138	3,8929	7,4251	9,7267
s	2	Creatine	3,9218	3,9141	2,0161	2,8071
dd	1	Tyrosine	3,9759	3,9389	9,6787	9,4104
m	2	Serine	4,0051	3,9761	18,3808	19,4781
s	2	Creatinine	4,0133	4,0054	2,1756	2,0954
m	2	Choline	4,0423	4,0356	0,5806	0,7590
-	-	Unknown 19	4,052	4,0427	1,0093	1,0510
-	-	Unknown 20	4,0608	4,0528	1,1298	1,1448
-	-	Unknown 21	4,0881	4,0611	3,8134	4,1857
-	-	Unknown 22	4,0972	4,0906	0,9859	0,7746
q	1	Lactate	4,118	4,0972	11,0436	8,1236
q	1	Lactate	4,1289	4,1184	7,4988	4,5675

q	1	Lactate	4,1407	4,1291	3,3645	2,5526
-	-	Unknown 23	4,1519	4,1423	0,6542	0,7057
m	1	Treonine	4,2436	4,1836	4,4248	5,7267
-	-	Unknown 24	4,2759	4,2442	4,1610	2,4175
-	-	Unknown 25	5,6375	5,6064	1,7878	1,5003
d	1	Inosine	6,1597	6,1248	0,5605	0,6811
		Noise	6,4595	6,4394	-0,4956	-0,3115
d	2	Tyrosine	6,9311	6,8779	3,6760	2,9881
dd	1	Xanthurenate	7,1227	7,0595	0,8739	0,6919
d	2	Tyrosine	7,2147	7,1804	2,4732	2,6320
d	1	Phenylalanine	7,3525	7,2973	3,5527	2,9391
d	1	Phenylalanine	7,4036	7,3753	1,4664	0,9702
t	1	Phenylalanine	7,4547	7,4125	2,5742	2,4269
-	-	Unknown	7,5018	7,4604	0,6568	0,5387
s	1	Xanthine	7,8583	7,8134	1,3767	0,4750
s	2	GTP	8,0456	8,0185	0,8532	-0,0088
s	1	Inosine	8,2025	8,1871	3,3306	3,4422
s	1	Inosine	8,2305	8,2049	3,4175	3,5042
s	1	AMP	8,2851	8,2598	1,6834	1,3566
s	1	Oxypurinol	8,3917	8,3745	0,0910	0,0910
s	1	Formate	8,4729	8,4426	1,9864	1,0514
s	1	AMP	8,6282	8,5897	1,3553	1,0353
-	-	Noise	9,5201	9,5	-0,0181	0,2357

Type	H's	Metabolite	ppm 1	ppm 2	Spectrum 3	Spectrum 4
s		TSP	0,0428	-0,0466	222,0756	242,6772
		Noise	0,5401	0,52	-0,0494	-0,1918
t	3	2-hydroxybutyrate	0,9229	0,8599	7,0695	6,8845
-	-	Unknown 1	0,9354	0,9247	1,3606	1,4199
t	3	Isoleucine	0,9479	0,9359	2,5864	3,4898
t	6	Leucine	0,9799	0,9503	21,0866	25,9675
d	3	Valine	1,0016	0,9799	7,6779	9,4928
d	3	Isoleucine	1,0248	1,0016	4,0619	4,9682
d	3	Valine	1,058	1,0337	6,7454	8,0422
-	-	Unknown 2	1,1007	1,0649	5,2767	3,2245
t	3	Ethanol	1,2478	1,1354	843,3500	559,3711
s	6	3-Hidroxyisovalerate	1,2576	1,2508	1,2382	1,4238
-	-	Unknown 3	1,3109	1,2737	10,8245	9,1573
d	3	Lactate	1,3503	1,3136	40,2770	50,6604
d	3	Alanine	1,5001	1,4645	24,7029	35,0226
m	2	2-hydroxybutyrate	1,6294	1,5908	14,9391	20,0964
m	2	2-hydroxybutyrate	1,6653	1,6299	16,1035	22,2080
m	2	Lysine	1,7974	1,6768	39,6172	66,3841
-	-	Unknown 4	1,8234	1,8096	0,7758	1,4778
-	-	Unknown 5	1,8582	1,8434	0,4829	1,2368
m	2	Lysine	1,9177	1,8747	8,2006	15,0822
s	3	Acetate	1,9285	1,9179	10,6815	13,4792
m	2	Glutamate	2,098	1,996	16,1038	17,2707
m	2	Glutamine	2,1356	2,1114	6,3424	4,7752
s	3	Hidroxyacetone	2,1498	2,1359	6,8311	6,4105
m	5	Methionine	2,1929	2,1506	7,6136	6,3454
s	-	Unknown 6	2,2326	2,2234	3,5485	1,9150
s	-	Unknown 7	2,2398	2,233	2,7631	1,6609
s	3	Acetoacetate	2,2706	2,258	4,2113	2,5764
m	1	Valine	2,2989	2,271	1,4429	1,9543
s	3	Methylacetoacetate	2,34	2,3302	0,5419	0,4141
-	-	Unknown 8	2,3522	2,3404	3,5692	2,0596
m	2	Glutamate	2,3651	2,3523	5,9133	3,5922
s	4	Succinate	2,3723	2,3652	1,8464	1,1957
s	3	Pyruvate	2,3801	2,3724	3,1272	2,2231
-	-	Unknown 9	2,4117	2,4035	0,5272	0,5847
-	-	Unknown 10	2,4674	2,4335	2,3742	2,1969
m	2	Glutamine	2,4983	2,4674	13,3043	16,1688
m	2	GSH+GSSG	2,5612	2,5522	1,0982	0,6719
d	2	Citrate	2,5952	2,5224	4,4604	0,4713
d	2	Citrate	2,6434	2,6285	0,6098	-0,2205
t	2	Methionine	2,663	2,6435	3,7519	2,6637
-	-	Unknown 11	2,673	2,6631	1,8508	0,6014
m	1	Aspartate	2,7038	2,6726	4,1486	1,2052

s	3	Sarcosine	2,7196	2,7089	-0,3218	-0,3965
-	-	Unknown 12	2,7377	2,7293	-0,1929	-0,5700
m	1	Aspartate	2,8541	2,7938	5,2340	-1,1516
s	9	Trimethylamine	2,8792	2,8591	0,1590	0,0484
dd	2	GSH+GSSG	2,9859	2,9316	0,6451	-0,9567
-	-	Unknown 13	3,0261	3,0147	2,5203	5,4043
s	3	Creatine	3,0391	3,0263	3,4752	7,1678
s	3	Creatinine	3,0539	3,0392	2,5357	4,5348
s	2	Malonate	3,1376	3,1285	1,6558	0,2665
m	1	Phenylalanine	3,1671	3,1378	13,0682	-0,5807
s	9	Choline	3,2082	3,1984	10,0903	6,2566
s	9	O-Phosphocoline	3,2143	3,2082	0,4871	0,5069
s	9	Trimethylamine N-oxide	3,2245	3,2146	4,3402	9,5129
s	9	Carnitine	3,2343	3,2247	10,5208	11,2801
s	9	Betaine	3,2416	3,2342	3,8658	5,6956
t	2	Taurine	3,2803	3,2419	86,2361	71,4721
m	1	Phenylalanine	3,3122	3,2904	3,5864	2,8446
s	3	Methanol	3,377	3,3531	31,6750	70,9738
t	2	Taurine	3,4458	3,4102	63,5972	41,7730
-	-	Unknown 14	3,5415	3,5165	12,3974	6,9135
m	4	Glycerol	3,5685	3,5438	381,9302	162,5451
s	2	Glycine	3,5908	3,5687	548,9337	232,1816
d	1	Treonine	3,5976	3,5909	8,2755	7,0517
d	1	Valine	3,6236	3,6086	8,1032	6,5836
-	-	Unknown 15	3,6347	3,6244	7,6373	4,3890
m	4	Glycerol	3,664	3,6399	797,3021	396,1363
q	2	Ethanol	3,6888	3,664	591,2149	311,4482
-	-	Unknown 16	3,7264	3,7027	117,5836	108,4712
-	-	Unknown 17	3,7405	3,7346	5,8580	5,4620
m	1	Glutamate	3,7671	3,7415	26,6130	20,9663
dd + q	1	Serine + Alanine	3,818	3,7665	510,7258	221,7300
dd	1	Methionine	3,8593	3,8349	12,0276	6,2777
s	2	Betaine	3,8892	3,8839	0,8838	0,3296
-	-	Unknown 18	3,9138	3,8929	9,0027	4,2017
s	2	Creatine	3,9218	3,9141	3,0806	1,2021
dd	1	Tyrosine	3,9759	3,9389	8,7976	9,4238
m	2	Serine	4,0051	3,9761	14,4588	17,9003
s	2	Creatinine	4,0133	4,0054	1,7867	1,8283
m	2	Choline	4,0423	4,0356	0,1365	-0,4956
-	-	Unknown 19	4,052	4,0427	0,5446	-0,6728
-	-	Unknown 20	4,0608	4,0528	1,4901	-0,3805
-	-	Unknown 21	4,0881	4,0611	6,9542	4,0045
-	-	Unknown 22	4,0972	4,0906	0,8149	0,9213
q	1	Lactate	4,118	4,0972	6,7939	9,2449
q	1	Lactate	4,1289	4,1184	4,4927	5,3158

q	1	Lactate	4,1407	4,1291	2,3786	1,8536
-	-	Unknown 23	4,1519	4,1423	0,0071	-0,1647
m	1	Treonine	4,2436	4,1836	0,6918	-0,8590
-	-	Unknown 24	4,2759	4,2442	4,0072	3,8504
-	-	Unknown 25	5,6375	5,6064	1,6037	3,3146
d	1	Inosine	6,1597	6,1248	1,6660	-1,0649
		Noise	6,4595	6,4394	0,1236	0,1322
d	2	Tyrosine	6,9311	6,8779	5,0817	7,5077
dd	1	Xanthurenate	7,1227	7,0595	1,7895	2,6998
d	2	Tyrosine	7,2147	7,1804	3,4910	3,5621
d	1	Phenylalanine	7,3525	7,2973	3,7092	4,0648
d	1	Phenylalanine	7,4036	7,3753	1,5428	1,5392
t	1	Phenylalanine	7,4547	7,4125	2,7099	2,3463
-	-	Unknown	7,5018	7,4604	1,1190	-0,0354
s	1	Xanthine	7,8583	7,8134	0,8325	1,1039
s	2	GTP	8,0456	8,0185	0,6296	0,9326
s	1	Inosine	8,2025	8,1871	3,9323	3,1552
s	1	Inosine	8,2305	8,2049	3,6511	3,5705
s	1	AMP	8,2851	8,2598	2,8872	2,2034
s	1	Oxypurinol	8,3917	8,3745	-0,1071	-0,2387
s	1	Formate	8,4729	8,4426	1,1805	1,3248
s	1	AMP	8,6282	8,5897	1,3983	1,1318
-	-	Noise	9,5201	9,5	0,0276	-0,3428

Type	H's	Metabolite	ppm 1	ppm 2	Spectrum 5	Spectrum 6
s		TSP	0,0428	-0,0466	236,2218	235,3237
		Noise	0,5401	0,52	-0,5495	-0,0676
t	3	2-hydroxybutyrate	0,9229	0,8599	7,1826	5,8008
-	-	Unknown 1	0,9354	0,9247	1,6030	1,4656
t	3	Isoleucine	0,9479	0,9359	3,1737	2,7421
t	6	Leucine	0,9799	0,9503	22,9716	18,1004
d	3	Valine	1,0016	0,9799	7,9494	6,5202
d	3	Isoleucine	1,0248	1,0016	4,2276	4,1503
d	3	Valine	1,058	1,0337	6,9150	6,0122
-	-	Unknown 2	1,1007	1,0649	3,7061	3,4843
t	3	Ethanol	1,2478	1,1354	518,6903	405,3136
s	6	3-Hidroxyisovalerate	1,2576	1,2508	0,7963	0,7903
-	-	Unknown 3	1,3109	1,2737	8,1944	6,8270
d	3	Lactate	1,3503	1,3136	24,5418	34,9930
d	3	Alanine	1,5001	1,4645	24,0155	21,9132
m	2	2-hydroxybutyrate	1,6294	1,5908	19,6932	20,3502
m	2	2-hydroxybutyrate	1,6653	1,6299	20,5870	20,0658
m	2	Lysine	1,7974	1,6768	50,8268	39,9046
-	-	Unknown 4	1,8234	1,8096	0,8083	1,0313
-	-	Unknown 5	1,8582	1,8434	0,8921	0,4448
m	2	Lysine	1,9177	1,8747	11,0230	7,8608
s	3	Acetate	1,9285	1,9179	10,9251	9,1728
m	2	Glutamate	2,098	1,996	14,6696	18,2901
m	2	Glutamine	2,1356	2,1114	4,5900	6,8471
s	3	Hidroxyacetone	2,1498	2,1359	6,0301	7,1256
m	5	Methionine	2,1929	2,1506	5,4719	9,6649
s	-	Unknown 6	2,2326	2,2234	2,7768	1,5011
s	-	Unknown 7	2,2398	2,233	1,6299	1,2858
s	3	Acetoacetate	2,2706	2,258	2,5830	3,5994
m	1	Valine	2,2989	2,271	1,4244	1,4298
s	3	Methylacetoacetate	2,34	2,3302	0,4716	0,6646
-	-	Unknown 8	2,3522	2,3404	1,8406	3,8289
m	2	Glutamate	2,3651	2,3523	2,9074	5,8935
s	4	Succinate	2,3723	2,3652	1,1394	1,9337
s	3	Pyruvate	2,3801	2,3724	1,4202	3,5642
-	-	Unknown 9	2,4117	2,4035	0,3617	0,3534
-	-	Unknown 10	2,4674	2,4335	2,6237	3,1227
m	2	Glutamine	2,4983	2,4674	17,4466	17,4207
m	2	GSH+GSSG	2,5612	2,5522	0,7545	1,9102
d	2	Citrate	2,5952	2,5224	2,5371	6,7219
d	2	Citrate	2,6434	2,6285	0,5877	1,0951
t	2	Methionine	2,663	2,6435	2,9619	4,2674
-	-	Unknown 11	2,673	2,6631	1,1848	1,6017
m	1	Aspartate	2,7038	2,6726	3,2322	3,7243

s	3	Sarcosine	2,7196	2,7089	-0,1360	-0,7382
-	-	Unknown 12	2,7377	2,7293	-0,0258	-0,4682
m	1	Aspartate	2,8541	2,7938	3,7567	6,0963
s	9	Trimethylamine	2,8792	2,8591	0,3482	0,1241
dd	2	GSH+GSSG	2,9859	2,9316	1,0785	2,2617
-	-	Unknown 13	3,0261	3,0147	3,5084	2,0138
s	3	Creatine	3,0391	3,0263	4,9082	2,7818
s	3	Creatinine	3,0539	3,0392	2,9998	2,4966
s	2	Malonate	3,1376	3,1285	1,0432	1,8460
m	1	Phenylalanine	3,1671	3,1378	1,5180	13,0700
s	9	Choline	3,2082	3,1984	4,9458	10,6617
s	9	O-Phosphocoline	3,2143	3,2082	0,9483	0,8648
s	9	Trimethylamine N-oxide	3,2245	3,2146	9,8425	6,5879
s	9	Carnitine	3,2343	3,2247	11,2829	12,3780
s	9	Betaine	3,2416	3,2342	5,8774	4,7177
t	2	Taurine	3,2803	3,2419	63,6625	93,2228
m	1	Phenylalanine	3,3122	3,2904	1,8751	3,7731
s	3	Methanol	3,377	3,3531	75,9308	69,3581
t	2	Taurine	3,4458	3,4102	36,0550	65,0763
-	-	Unknown 14	3,5415	3,5165	5,9475	9,7271
m	4	Glycerol	3,5685	3,5438	119,4863	203,6318
s	2	Glycine	3,5908	3,5687	163,5526	268,9456
d	1	Treonine	3,5976	3,5909	3,6202	4,2854
d	1	Valine	3,6236	3,6086	5,2774	5,6626
-	-	Unknown 15	3,6347	3,6244	4,2261	6,6975
m	4	Glycerol	3,664	3,6399	336,8951	404,3163
q	2	Ethanol	3,6888	3,664	253,3843	281,0075
-	-	Unknown 16	3,7264	3,7027	197,4955	88,0153
-	-	Unknown 17	3,7405	3,7346	9,0571	4,5804
m	1	Glutamate	3,7671	3,7415	29,7617	22,3785
dd + q	1	Serine + Alanine	3,818	3,7665	166,6136	270,2243
dd	1	Methionine	3,8593	3,8349	6,8864	10,1223
s	2	Betaine	3,8892	3,8839	0,5896	1,7160
-	-	Unknown 18	3,9138	3,8929	5,8987	10,4833
s	2	Creatine	3,9218	3,9141	1,6489	2,9755
dd	1	Tyrosine	3,9759	3,9389	8,1668	7,3990
m	2	Serine	4,0051	3,9761	18,8469	13,0724
s	2	Creatinine	4,0133	4,0054	1,4182	2,2184
m	2	Choline	4,0423	4,0356	0,2648	0,8616
-	-	Unknown 19	4,052	4,0427	0,7628	0,8701
-	-	Unknown 20	4,0608	4,0528	0,9662	1,0202
-	-	Unknown 21	4,0881	4,0611	4,6465	4,7527
-	-	Unknown 22	4,0972	4,0906	0,9108	0,7537
q	1	Lactate	4,118	4,0972	4,8650	6,3706
q	1	Lactate	4,1289	4,1184	2,3946	4,2894

q	1	Lactate	4,1407	4,1291	1,6811	2,5466
-	-	Unknown 23	4,1519	4,1423	0,4221	0,5195
m	1	Treonine	4,2436	4,1836	2,6250	5,2164
-	-	Unknown 24	4,2759	4,2442	3,2715	3,6660
-	-	Unknown 25	5,6375	5,6064	1,4202	0,3287
d	1	Inosine	6,1597	6,1248	0,0093	3,4435
		Noise	6,4595	6,4394	-0,2651	0,3688
d	2	Tyrosine	6,9311	6,8779	3,8469	4,2362
dd	1	Xanthurenate	7,1227	7,0595	0,3755	2,3628
d	2	Tyrosine	7,2147	7,1804	3,0974	2,6825
d	1	Phenylalanine	7,3525	7,2973	3,2042	4,2342
d	1	Phenylalanine	7,4036	7,3753	0,8264	0,9900
t	1	Phenylalanine	7,4547	7,4125	2,5055	3,1606
-	-	Unknown	7,5018	7,4604	0,8801	1,5361
s	1	Xanthine	7,8583	7,8134	0,6347	0,8290
s	2	GTP	8,0456	8,0185	0,2081	0,8595
s	1	Inosine	8,2025	8,1871	4,1781	3,6662
s	1	Inosine	8,2305	8,2049	4,1008	3,9651
s	1	AMP	8,2851	8,2598	0,6378	3,7544
s	1	Oxypurinol	8,3917	8,3745	0,1096	0,3755
s	1	Formate	8,4729	8,4426	1,1827	1,1474
s	1	AMP	8,6282	8,5897	0,6090	3,2369
-	-	Noise	9,5201	9,5	0,1359	0,2425

Type	H's	Metabolite	ppm 1	ppm 2	Spectrum 7	Spectrum 8
s		TSP	0,0428	-0,0466	114,3457	109,7551
		Noise	0,5401	0,52	0,7118	0,7842
t	3	2-hydroxybutyrate	0,9229	0,8599	54,8296	54,8007
-	-	Unknown 1	0,9354	0,9247	12,9317	13,0497
t	3	Isoleucine	0,9479	0,9359	16,4698	16,4862
t	6	Leucine	0,9799	0,9503	40,4253	40,1956
d	3	Valine	1,0016	0,9799	14,8165	14,2065
d	3	Isoleucine	1,0248	1,0016	8,3961	8,4361
d	3	Valine	1,058	1,0337	11,2305	10,4585
-	-	Unknown 2	1,1007	1,0649	4,4792	4,5469
t	3	Ethanol	1,2478	1,1354	49,2114	45,4972
s	6	3-Hidroxyisovalerate	1,2576	1,2508	2,0788	2,0499
-	-	Unknown 3	1,3109	1,2737	8,6785	8,5716
d	3	Lactate	1,3503	1,3136	26,6044	19,5865
d	3	Alanine	1,5001	1,4645	40,2630	38,2163
m	2	2-hydroxybutyrate	1,6294	1,5908	14,9637	14,8539
m	2	2-hydroxybutyrate	1,6653	1,6299	18,2085	18,3752
m	2	Lysine	1,7974	1,6768	87,7416	91,2705
-	-	Unknown 4	1,8234	1,8096	4,9964	5,6339
-	-	Unknown 5	1,8582	1,8434	5,7974	6,5239
m	2	Lysine	1,9177	1,8747	30,3974	28,7542
s	3	Acetate	1,9285	1,9179	11,1477	11,0324
m	2	Glutamate	2,098	1,996	61,6184	60,7259
m	2	Glutamine	2,1356	2,1114	11,6019	11,6840
s	3	Hidroxyacetone	2,1498	2,1359	7,9613	7,9408
m	5	Methionine	2,1929	2,1506	10,0712	10,2696
s	-	Unknown 6	2,2326	2,2234	1,8676	2,0344
s	-	Unknown 7	2,2398	2,233	2,7091	2,3520
s	3	Acetoacetate	2,2706	2,258	5,5672	5,3998
m	1	Valine	2,2989	2,271	12,5745	13,0552
s	3	Methylacetoacetate	2,34	2,3302	2,6559	2,7712
-	-	Unknown 8	2,3522	2,3404	4,3902	4,0238
m	2	Glutamate	2,3651	2,3523	5,3949	5,0232
s	4	Succinate	2,3723	2,3652	2,5636	2,4353
s	3	Pyruvate	2,3801	2,3724	2,7887	2,6128
-	-	Unknown 9	2,4117	2,4035	1,1925	1,4025
-	-	Unknown 10	2,4674	2,4335	4,8744	5,2917
m	2	Glutamine	2,4983	2,4674	2,0136	2,6527
m	2	GSH+GSSG	2,5612	2,5522	1,5585	1,4538
d	2	Citrate	2,5952	2,5224	8,1282	9,0986
d	2	Citrate	2,6434	2,6285	2,4219	2,4408
t	2	Methionine	2,663	2,6435	4,6574	4,6443
-	-	Unknown 11	2,673	2,6631	1,7017	2,0863
m	1	Aspartate	2,7038	2,6726	5,2710	5,9197

s	3	Sarcosine	2,7196	2,7089	1,5753	1,7567
-	-	Unknown 12	2,7377	2,7293	1,0061	1,1893
m	1	Aspartate	2,8541	2,7938	6,6413	8,5025
s	9	Trimethylamine	2,8792	2,8591	2,9999	3,5412
dd	2	GSH+GSSG	2,9859	2,9316	9,8378	11,2367
-	-	Unknown 13	3,0261	3,0147	10,8433	11,6414
s	3	Creatine	3,0391	3,0263	11,1805	11,1676
s	3	Creatinine	3,0539	3,0392	6,8212	6,8158
s	2	Malonate	3,1376	3,1285	2,0377	2,2659
m	1	Phenylalanine	3,1671	3,1378	4,8189	5,8325
s	9	Choline	3,2082	3,1984	5,6409	4,6212
s	9	O-Phosphocoline	3,2143	3,2082	1,9540	2,0615
s	9	Trimethylamine N-oxide	3,2245	3,2146	8,3268	8,4573
s	9	Carnitine	3,2343	3,2247	8,1592	8,3945
s	9	Betaine	3,2416	3,2342	4,9241	5,2429
t	2	Taurine	3,2803	3,2419	31,5536	28,2159
m	1	Phenylalanine	3,3122	3,2904	2,6628	2,9576
s	3	Methanol	3,377	3,3531	5,3355	5,6126
t	2	Taurine	3,4458	3,4102	20,3621	17,8191
-	-	Unknown 14	3,5415	3,5165	1,6195	2,3011
m	4	Glycerol	3,5685	3,5438	8,8115	8,4735
s	2	Glycine	3,5908	3,5687	3,7557	3,9296
d	1	Treonine	3,5976	3,5909	2,1514	2,0337
d	1	Valine	3,6236	3,6086	4,2866	4,8271
-	-	Unknown 15	3,6347	3,6244	1,8136	2,4100
m	4	Glycerol	3,664	3,6399	9,8190	9,3473
q	2	Ethanol	3,6888	3,664	11,0327	10,7607
-	-	Unknown 16	3,7264	3,7027	2,0089	3,2281
-	-	Unknown 17	3,7405	3,7346	1,6475	1,8376
m	1	Glutamate	3,7671	3,7415	14,8733	14,2387
dd + q	1	Serine + Alanine	3,818	3,7665	29,9214	29,2479
dd	1	Methionine	3,8593	3,8349	11,0306	11,5351
s	2	Betaine	3,8892	3,8839	2,5990	2,6538
-	-	Unknown 18	3,9138	3,8929	9,7646	10,0673
s	2	Creatine	3,9218	3,9141	2,9779	3,0928
dd	1	Tyrosine	3,9759	3,9389	17,0980	18,0011
m	2	Serine	4,0051	3,9761	18,7446	20,2102
s	2	Creatinine	4,0133	4,0054	2,8371	3,0846
m	2	Choline	4,0423	4,0356	1,6217	1,7064
-	-	Unknown 19	4,052	4,0427	2,2309	2,4700
-	-	Unknown 20	4,0608	4,0528	1,8808	2,1630
-	-	Unknown 21	4,0881	4,0611	5,5731	6,7070
-	-	Unknown 22	4,0972	4,0906	1,7089	1,7866
q	1	Lactate	4,118	4,0972	6,9890	6,7910
q	1	Lactate	4,1289	4,1184	4,0978	3,6168

q	1	Lactate	4,1407	4,1291	3,9640	4,2128
-	-	Unknown 23	4,1519	4,1423	2,5918	2,9458
m	1	Treonine	4,2436	4,1836	16,7854	18,3303
-	-	Unknown 24	4,2759	4,2442	10,8734	11,9638
-	-	Unknown 25	5,6375	5,6064	1,8861	1,9399
d	1	Inosine	6,1597	6,1248	-0,1424	-0,3474
		Noise	6,4595	6,4394	-0,0829	-0,1181
d	2	Tyrosine	6,9311	6,8779	5,5547	5,4725
dd	1	Xanthurenate	7,1227	7,0595	5,1672	4,8850
d	2	Tyrosine	7,2147	7,1804	4,6531	4,7581
d	1	Phenylalanine	7,3525	7,2973	8,4781	8,1344
d	1	Phenylalanine	7,4036	7,3753	2,3263	2,5717
t	1	Phenylalanine	7,4547	7,4125	3,6592	3,7714
-	-	Unknown	7,5018	7,4604	1,5314	1,4817
s	1	Xanthine	7,8583	7,8134	3,3058	3,5606
s	2	GTP	8,0456	8,0185	2,4539	2,1054
s	1	Inosine	8,2025	8,1871	3,1718	2,8116
s	1	Inosine	8,2305	8,2049	3,8003	3,4513
s	1	AMP	8,2851	8,2598	1,7000	1,4407
s	1	Oxypurinol	8,3917	8,3745	0,7819	0,7124
s	1	Formate	8,4729	8,4426	1,1753	1,4822
s	1	AMP	8,6282	8,5897	0,7469	0,5028
-	-	Noise	9,5201	9,5	-0,2282	0,0000

Type	H's	Metabolite	ppm 1	ppm 2	Spectrum 9	Spectrum 10
s		TSP	0,0428	-0,0466	120,9491	108,7481
		Noise	0,5401	0,52	0,9039	0,8094
t	3	2-hydroxybutyrate	0,9229	0,8599	52,1791	58,5125
-	-	Unknown 1	0,9354	0,9247	11,6882	13,5540
t	3	Isoleucine	0,9479	0,9359	14,3635	17,0237
t	6	Leucine	0,9799	0,9503	33,4882	39,1660
d	3	Valine	1,0016	0,9799	12,4568	14,2000
d	3	Isoleucine	1,0248	1,0016	8,0906	8,5524
d	3	Valine	1,058	1,0337	9,5178	10,3835
-	-	Unknown 2	1,1007	1,0649	5,5132	5,1179
t	3	Ethanol	1,2478	1,1354	46,4821	43,0778
s	6	3-Hidroxyisovalerate	1,2576	1,2508	2,4549	2,2316
-	-	Unknown 3	1,3109	1,2737	10,3879	9,5475
d	3	Lactate	1,3503	1,3136	25,6534	26,3179
d	3	Alanine	1,5001	1,4645	31,1276	38,9226
m	2	2-hydroxybutyrate	1,6294	1,5908	13,9569	15,8031
m	2	2-hydroxybutyrate	1,6653	1,6299	16,9036	19,2909
m	2	Lysine	1,7974	1,6768	73,5754	90,0516
-	-	Unknown 4	1,8234	1,8096	5,1279	5,4417
-	-	Unknown 5	1,8582	1,8434	5,5016	6,5158
m	2	Lysine	1,9177	1,8747	23,3984	29,7799
s	3	Acetate	1,9285	1,9179	9,6805	11,0072
m	2	Glutamate	2,098	1,996	61,6338	63,8978
m	2	Glutamine	2,1356	2,1114	12,8365	11,6384
s	3	Hidroxyacetone	2,1498	2,1359	8,6357	7,9359
m	5	Methionine	2,1929	2,1506	12,7038	10,5631
s	-	Unknown 6	2,2326	2,2234	2,1345	1,9130
s	-	Unknown 7	2,2398	2,233	2,1871	1,8604
s	3	Acetoacetate	2,2706	2,258	6,2832	5,5739
m	1	Valine	2,2989	2,271	11,7836	12,7889
s	3	Methylacetoacetate	2,34	2,3302	2,6711	2,6850
-	-	Unknown 8	2,3522	2,3404	4,9732	4,2215
m	2	Glutamate	2,3651	2,3523	6,4861	5,1109
s	4	Succinate	2,3723	2,3652	2,8261	2,3840
s	3	Pyruvate	2,3801	2,3724	3,5610	2,7312
-	-	Unknown 9	2,4117	2,4035	1,5069	1,2783
-	-	Unknown 10	2,4674	2,4335	5,1406	4,5932
m	2	Glutamine	2,4983	2,4674	2,7988	2,2680
m	2	GSH+GSSG	2,5612	2,5522	1,9395	1,6567
d	2	Citrate	2,5952	2,5224	11,0228	8,6997
d	2	Citrate	2,6434	2,6285	2,4383	2,3769
t	2	Methionine	2,663	2,6435	5,3625	4,5755
-	-	Unknown 11	2,673	2,6631	2,2039	1,8940
m	1	Aspartate	2,7038	2,6726	6,3640	5,3200

s	3	Sarcosine	2,7196	2,7089	1,6240	1,5216
-	-	Unknown 12	2,7377	2,7293	1,0618	1,1203
m	1	Aspartate	2,8541	2,7938	8,3111	6,8215
s	9	Trimethylamine	2,8792	2,8591	2,7894	3,1722
dd	2	GSH+GSSG	2,9859	2,9316	10,9821	10,4353
-	-	Unknown 13	3,0261	3,0147	8,2961	11,1551
s	3	Creatine	3,0391	3,0263	7,6667	10,8053
s	3	Creatinine	3,0539	3,0392	5,3604	6,6342
s	2	Malonate	3,1376	3,1285	2,4127	1,9044
m	1	Phenylalanine	3,1671	3,1378	11,2737	4,9161
s	9	Choline	3,2082	3,1984	7,3135	5,9278
s	9	O-Phosphocoline	3,2143	3,2082	1,8567	2,0585
s	9	Trimethylamine N-oxide	3,2245	3,2146	5,9002	8,7282
s	9	Carnitine	3,2343	3,2247	9,8913	8,7469
s	9	Betaine	3,2416	3,2342	4,1596	4,9198
t	2	Taurine	3,2803	3,2419	40,2029	31,4728
m	1	Phenylalanine	3,3122	3,2904	3,5875	2,3726
s	3	Methanol	3,377	3,3531	5,7706	4,3992
t	2	Taurine	3,4458	3,4102	31,4353	20,4497
-	-	Unknown 14	3,5415	3,5165	2,9383	1,6180
m	4	Glycerol	3,5685	3,5438	14,2742	7,4384
s	2	Glycine	3,5908	3,5687	10,1495	3,4778
d	1	Treonine	3,5976	3,5909	1,9732	2,0172
d	1	Valine	3,6236	3,6086	4,4236	3,8994
-	-	Unknown 15	3,6347	3,6244	2,8103	2,0001
m	4	Glycerol	3,664	3,6399	15,4332	6,9876
q	2	Ethanol	3,6888	3,664	14,8534	8,3011
-	-	Unknown 16	3,7264	3,7027	3,1150	1,8267
-	-	Unknown 17	3,7405	3,7346	1,7046	1,5630
m	1	Glutamate	3,7671	3,7415	13,8903	13,8616
dd + q	1	Serine + Alanine	3,818	3,7665	34,9949	28,9441
dd	1	Methionine	3,8593	3,8349	13,7649	10,5544
s	2	Betaine	3,8892	3,8839	2,9162	2,6796
-	-	Unknown 18	3,9138	3,8929	11,3081	9,8104
s	2	Creatine	3,9218	3,9141	3,6154	3,0384
dd	1	Tyrosine	3,9759	3,9389	16,2072	16,7827
m	2	Serine	4,0051	3,9761	17,3742	18,1448
s	2	Creatinine	4,0133	4,0054	3,0779	2,8458
m	2	Choline	4,0423	4,0356	1,6323	1,6040
-	-	Unknown 19	4,052	4,0427	2,2999	2,1572
-	-	Unknown 20	4,0608	4,0528	1,9984	1,8661
-	-	Unknown 21	4,0881	4,0611	7,5538	4,9320
-	-	Unknown 22	4,0972	4,0906	1,6096	1,5136
q	1	Lactate	4,118	4,0972	7,2503	6,4043
q	1	Lactate	4,1289	4,1184	4,1949	3,6316

q	1	Lactate	4,1407	4,1291	3,9161	3,7964
-	-	Unknown 23	4,1519	4,1423	2,4092	2,3737
m	1	Treonine	4,2436	4,1836	16,4284	15,8379
-	-	Unknown 24	4,2759	4,2442	11,7503	9,5316
-	-	Unknown 25	5,6375	5,6064	1,6811	2,6352
d	1	Inosine	6,1597	6,1248	0,4884	0,4641
		Noise	6,4595	6,4394	-0,0632	0,1104
d	2	Tyrosine	6,9311	6,8779	4,9922	5,5324
dd	1	Xanthurenate	7,1227	7,0595	4,8810	5,9476
d	2	Tyrosine	7,2147	7,1804	4,2436	4,6174
d	1	Phenylalanine	7,3525	7,2973	7,4204	8,3773
d	1	Phenylalanine	7,4036	7,3753	2,1900	2,4537
t	1	Phenylalanine	7,4547	7,4125	3,4891	3,4649
-	-	Unknown	7,5018	7,4604	1,0804	1,3406
s	1	Xanthine	7,8583	7,8134	3,1119	3,5107
s	2	GTP	8,0456	8,0185	2,3248	2,3263
s	1	Inosine	8,2025	8,1871	3,5481	3,0523
s	1	Inosine	8,2305	8,2049	4,5054	3,6721
s	1	AMP	8,2851	8,2598	2,3661	1,8946
s	1	Oxypurinol	8,3917	8,3745	0,9926	0,8308
s	1	Formate	8,4729	8,4426	1,6009	1,4329
s	1	AMP	8,6282	8,5897	1,3368	0,9782
-	-	Noise	9,5201	9,5	-0,1836	-0,0424

Type	H's	Metabolite	ppm 1	ppm 2	Spectrum 11	Spectrum 12
s		TSP	0,0428	-0,0466	128,5449	131,4987
		Noise	0,5401	0,52	0,8913	0,4013
t	3	2-hydroxybutyrate	0,9229	0,8599	54,3922	62,4599
-	-	Unknown 1	0,9354	0,9247	12,5620	14,2374
t	3	Isoleucine	0,9479	0,9359	15,9959	18,0952
t	6	Leucine	0,9799	0,9503	38,0785	37,3233
d	3	Valine	1,0016	0,9799	13,5089	12,6392
d	3	Isoleucine	1,0248	1,0016	8,2181	8,3224
d	3	Valine	1,058	1,0337	9,6506	7,7527
-	-	Unknown 2	1,1007	1,0649	4,9142	4,9124
t	3	Ethanol	1,2478	1,1354	47,0734	46,3384
s	6	3-Hidroxyisovalerate	1,2576	1,2508	2,1878	2,1673
-	-	Unknown 3	1,3109	1,2737	9,0724	8,9027
d	3	Lactate	1,3503	1,3136	19,9744	24,6980
d	3	Alanine	1,5001	1,4645	35,4539	31,8419
m	2	2-hydroxybutyrate	1,6294	1,5908	14,9192	15,7861
m	2	2-hydroxybutyrate	1,6653	1,6299	18,3783	19,6602
m	2	Lysine	1,7974	1,6768	88,5022	89,7779
-	-	Unknown 4	1,8234	1,8096	5,7876	6,2001
-	-	Unknown 5	1,8582	1,8434	6,4900	6,7516
m	2	Lysine	1,9177	1,8747	27,0381	23,3466
s	3	Acetate	1,9285	1,9179	10,5847	9,3274
m	2	Glutamate	2,098	1,996	60,6256	68,1489
m	2	Glutamine	2,1356	2,1114	11,4209	13,2992
s	3	Hidroxyacetone	2,1498	2,1359	7,6140	8,1183
m	5	Methionine	2,1929	2,1506	11,0461	13,7484
s	-	Unknown 6	2,2326	2,2234	2,0295	2,1103
s	-	Unknown 7	2,2398	2,233	1,9001	1,9707
s	3	Acetoacetate	2,2706	2,258	5,5069	6,5155
m	1	Valine	2,2989	2,271	12,6541	13,1364
s	3	Methylacetoacetate	2,34	2,3302	2,7621	2,9088
-	-	Unknown 8	2,3522	2,3404	3,8898	4,7779
m	2	Glutamate	2,3651	2,3523	4,8571	6,2340
s	4	Succinate	2,3723	2,3652	2,3082	3,0290
s	3	Pyruvate	2,3801	2,3724	2,7666	3,6639
-	-	Unknown 9	2,4117	2,4035	1,4034	1,6505
-	-	Unknown 10	2,4674	2,4335	5,2247	3,7470
m	2	Glutamine	2,4983	2,4674	2,8055	1,9659
m	2	GSH+GSSG	2,5612	2,5522	1,6414	2,1375
d	2	Citrate	2,5952	2,5224	9,6950	9,8287
d	2	Citrate	2,6434	2,6285	2,5615	2,1422
t	2	Methionine	2,663	2,6435	4,9558	5,0437
-	-	Unknown 11	2,673	2,6631	2,0833	2,2908
m	1	Aspartate	2,7038	2,6726	6,3525	6,2327

s	3	Sarcosine	2,7196	2,7089	1,8782	1,4481
-	-	Unknown 12	2,7377	2,7293	1,2133	0,9233
m	1	Aspartate	2,8541	2,7938	8,8675	6,7088
s	9	Trimethylamine	2,8792	2,8591	3,4533	2,2364
dd	2	GSH+GSSG	2,9859	2,9316	11,9480	12,2091
-	-	Unknown 13	3,0261	3,0147	11,0215	10,1742
s	3	Creatine	3,0391	3,0263	10,0870	7,8960
s	3	Creatinine	3,0539	3,0392	6,3766	5,1341
s	2	Malonate	3,1376	3,1285	2,2377	2,0851
m	1	Phenylalanine	3,1671	3,1378	5,4478	10,6362
s	9	Choline	3,2082	3,1984	4,9595	6,9394
s	9	O-Phosphocoline	3,2143	3,2082	2,1714	2,3193
s	9	Trimethylamine N-oxide	3,2245	3,2146	8,8407	5,8788
s	9	Carnitine	3,2343	3,2247	9,1297	9,2025
s	9	Betaine	3,2416	3,2342	5,2700	4,4278
t	2	Taurine	3,2803	3,2419	29,6558	42,2815
m	1	Phenylalanine	3,3122	3,2904	2,8730	3,3086
s	3	Methanol	3,377	3,3531	5,9532	4,7755
t	2	Taurine	3,4458	3,4102	19,6392	31,8724
-	-	Unknown 14	3,5415	3,5165	2,1860	1,9258
m	4	Glycerol	3,5685	3,5438	7,5428	10,0654
s	2	Glycine	3,5908	3,5687	3,5369	7,8029
d	1	Treonine	3,5976	3,5909	1,8960	0,8589
d	1	Valine	3,6236	3,6086	4,4586	1,5802
-	-	Unknown 15	3,6347	3,6244	2,5162	2,0711
m	4	Glycerol	3,664	3,6399	9,6912	13,7595
q	2	Ethanol	3,6888	3,664	10,6381	12,1509
-	-	Unknown 16	3,7264	3,7027	2,3929	-1,4125
-	-	Unknown 17	3,7405	3,7346	1,4874	-0,2164
m	1	Glutamate	3,7671	3,7415	13,3002	5,4639
dd + q	1	Serine + Alanine	3,818	3,7665	28,3752	29,9376
dd	1	Methionine	3,8593	3,8349	11,1342	13,2630
s	2	Betaine	3,8892	3,8839	2,3896	2,4631
-	-	Unknown 18	3,9138	3,8929	9,7711	9,2829
s	2	Creatine	3,9218	3,9141	3,0413	2,6833
dd	1	Tyrosine	3,9759	3,9389	16,8975	14,7706
m	2	Serine	4,0051	3,9761	19,0195	13,0492
s	2	Creatinine	4,0133	4,0054	2,5004	2,3908
m	2	Choline	4,0423	4,0356	1,6947	0,8063
-	-	Unknown 19	4,052	4,0427	2,6599	1,0712
-	-	Unknown 20	4,0608	4,0528	2,3578	1,8156
-	-	Unknown 21	4,0881	4,0611	6,8042	8,0545
-	-	Unknown 22	4,0972	4,0906	1,8767	1,4173
q	1	Lactate	4,118	4,0972	6,6764	5,7255
q	1	Lactate	4,1289	4,1184	3,2121	2,7176

q	1	Lactate	4,1407	4,1291	3,5549	2,5130
-	-	Unknown 23	4,1519	4,1423	2,6813	1,0981
m	1	Treonine	4,2436	4,1836	17,0219	7,8920
-	-	Unknown 24	4,2759	4,2442	11,3234	7,8724
-	-	Unknown 25	5,6375	5,6064	2,8361	3,3487
d	1	Inosine	6,1597	6,1248	-0,8231	-1,7086
		Noise	6,4595	6,4394	-0,0367	-0,5206
d	2	Tyrosine	6,9311	6,8779	4,8492	3,0972
dd	1	Xanthurenate	7,1227	7,0595	4,9086	3,9617
d	2	Tyrosine	7,2147	7,1804	4,3445	2,4111
d	1	Phenylalanine	7,3525	7,2973	7,4458	5,8227
d	1	Phenylalanine	7,4036	7,3753	2,1193	0,8678
t	1	Phenylalanine	7,4547	7,4125	3,0776	0,7475
-	-	Unknown	7,5018	7,4604	1,3922	0,0496
s	1	Xanthine	7,8583	7,8134	3,5004	1,9239
s	2	GTP	8,0456	8,0185	2,0415	1,7044
s	1	Inosine	8,2025	8,1871	3,0659	2,1277
s	1	Inosine	8,2305	8,2049	3,7174	2,9241
s	1	AMP	8,2851	8,2598	1,3142	3,0722
s	1	Oxypurinol	8,3917	8,3745	0,7137	0,4038
s	1	Formate	8,4729	8,4426	1,2678	0,8347
s	1	AMP	8,6282	8,5897	0,6366	1,8250
-	-	Noise	9,5201	9,5	0,0384	-0,1870

Type	H's	Metabolite	ppm 1	ppm 2	Spectrum 13	Spectrum 14
s		TSP	0,0428	-0,0466	406,1296	366,9569
		Noise	0,5401	0,52	-0,0964	0,2020
t	3	2-hydroxybutyrate	0,9229	0,8599	24,6584	32,2146
-	-	Unknown 1	0,9354	0,9247	4,2471	5,0945
t	3	Isoleucine	0,9479	0,9359	5,9798	6,7952
t	6	Leucine	0,9799	0,9503	11,0936	13,0890
d	3	Valine	1,0016	0,9799	3,9686	4,5475
d	3	Isoleucine	1,0248	1,0016	1,8008	3,2583
d	3	Valine	1,058	1,0337	2,5646	4,1009
-	-	Unknown 2	1,1007	1,0649	0,8678	4,6941
t	3	Ethanol	1,2478	1,1354	21,8682	36,6711
s	6	3-Hidroxyisovalerate	1,2576	1,2508	2,5115	3,9350
-	-	Unknown 3	1,3109	1,2737	32,1056	36,4735
d	3	Lactate	1,3503	1,3136	49,5687	55,9115
d	3	Alanine	1,5001	1,4645	12,9852	15,3455
m	2	2-hydroxybutyrate	1,6294	1,5908	4,7585	6,3200
m	2	2-hydroxybutyrate	1,6653	1,6299	6,3013	8,5030
m	2	Lysine	1,7974	1,6768	27,1587	29,7590
-	-	Unknown 4	1,8234	1,8096	1,2209	2,1969
-	-	Unknown 5	1,8582	1,8434	1,7974	2,8838
m	2	Lysine	1,9177	1,8747	7,4324	9,5557
s	3	Acetate	1,9285	1,9179	10,3366	9,8317
m	2	Glutamate	2,098	1,996	32,0469	35,9374
m	2	Glutamine	2,1356	2,1114	6,3678	7,4533
s	3	Hidroxyacetone	2,1498	2,1359	4,7078	5,2807
m	5	Methionine	2,1929	2,1506	11,5136	12,1919
s	-	Unknown 6	2,2326	2,2234	0,6082	1,7501
s	-	Unknown 7	2,2398	2,233	0,6005	1,3734
s	3	Acetoacetate	2,2706	2,258	4,6259	4,3057
m	1	Valine	2,2989	2,271	6,8332	7,8939
s	3	Methylacetoacetate	2,34	2,3302	0,9609	1,6660
-	-	Unknown 8	2,3522	2,3404	3,3911	3,4808
m	2	Glutamate	2,3651	2,3523	4,7197	5,1975
s	4	Succinate	2,3723	2,3652	1,7936	1,9716
s	3	Pyruvate	2,3801	2,3724	2,9057	3,1163
-	-	Unknown 9	2,4117	2,4035	1,4047	1,7062
-	-	Unknown 10	2,4674	2,4335	0,8409	2,7451
m	2	Glutamine	2,4983	2,4674	0,0798	1,0624
m	2	GSH+GSSG	2,5612	2,5522	2,8964	3,1646
d	2	Citrate	2,5952	2,5224	10,6959	12,4466
d	2	Citrate	2,6434	2,6285	0,7631	1,4238
t	2	Methionine	2,663	2,6435	4,3344	5,0843
-	-	Unknown 11	2,673	2,6631	1,8508	2,3451
m	1	Aspartate	2,7038	2,6726	5,0999	4,8857

s	3	Sarcosine	2,7196	2,7089	0,2977	0,2754
-	-	Unknown 12	2,7377	2,7293	1,5705	1,6723
m	1	Aspartate	2,8541	2,7938	4,7909	6,9240
s	9	Trimethylamine	2,8792	2,8591	1,1753	1,1166
dd	2	GSH+GSSG	2,9859	2,9316	4,4065	6,0888
-	-	Unknown 13	3,0261	3,0147	3,9242	2,8843
s	3	Creatine	3,0391	3,0263	1,7902	1,9753
s	3	Creatinine	3,0539	3,0392	1,1637	1,4297
s	2	Malonate	3,1376	3,1285	0,3164	0,5255
m	1	Phenylalanine	3,1671	3,1378	1,5373	3,0519
s	9	Choline	3,2082	3,1984	8,8361	8,1826
s	9	O-Phosphocoline	3,2143	3,2082	1,1049	1,3874
s	9	Trimethylamine N-oxide	3,2245	3,2146	17,3480	17,1267
s	9	Carnitine	3,2343	3,2247	16,7188	15,8819
s	9	Betaine	3,2416	3,2342	6,0156	5,8483
t	2	Taurine	3,2803	3,2419	64,8986	59,6405
m	1	Phenylalanine	3,3122	3,2904	3,5024	3,4788
s	3	Methanol	3,377	3,3531	10,0315	13,9148
t	2	Taurine	3,4458	3,4102	54,0621	51,0151
-	-	Unknown 14	3,5415	3,5165	2,8929	3,7027
m	4	Glycerol	3,5685	3,5438	12,2918	11,5537
s	2	Glycine	3,5908	3,5687	8,4679	8,6408
d	1	Treonine	3,5976	3,5909	1,5024	1,4626
d	1	Valine	3,6236	3,6086	2,7966	1,9541
-	-	Unknown 15	3,6347	3,6244	2,3314	1,2859
m	4	Glycerol	3,664	3,6399	12,1368	10,2808
q	2	Ethanol	3,6888	3,664	12,0761	9,6396
-	-	Unknown 16	3,7264	3,7027	1,8666	1,6350
-	-	Unknown 17	3,7405	3,7346	1,7138	1,3600
m	1	Glutamate	3,7671	3,7415	11,9261	11,9343
dd + q	1	Serine + Alanine	3,818	3,7665	32,4104	32,3891
dd	1	Methionine	3,8593	3,8349	7,5874	9,1704
s	2	Betaine	3,8892	3,8839	1,9846	2,0993
-	-	Unknown 18	3,9138	3,8929	8,9050	10,7066
s	2	Creatine	3,9218	3,9141	2,9736	2,4465
dd	1	Tyrosine	3,9759	3,9389	13,5773	12,6363
m	2	Serine	4,0051	3,9761	23,8833	24,2956
s	2	Creatinine	4,0133	4,0054	3,1263	4,6154
m	2	Choline	4,0423	4,0356	1,1902	1,5436
-	-	Unknown 19	4,052	4,0427	0,6057	1,5819
-	-	Unknown 20	4,0608	4,0528	0,9858	1,5268
-	-	Unknown 21	4,0881	4,0611	4,6606	5,0705
-	-	Unknown 22	4,0972	4,0906	0,4497	1,1750
q	1	Lactate	4,118	4,0972	7,5179	9,3976
q	1	Lactate	4,1289	4,1184	5,5918	6,5913

q	1	Lactate	4,1407	4,1291	3,3247	4,5302
-	-	Unknown 23	4,1519	4,1423	1,2319	2,6038
m	1	Treonine	4,2436	4,1836	9,1127	17,0592
-	-	Unknown 24	4,2759	4,2442	6,7008	10,3546
-	-	Unknown 25	5,6375	5,6064	-0,9236	-6,1065
d	1	Inosine	6,1597	6,1248	4,2065	-0,1682
		Noise	6,4595	6,4394	-1,3445	-2,8692
d	2	Tyrosine	6,9311	6,8779	-2,2785	-5,3759
dd	1	Xanthurenate	7,1227	7,0595	-2,1430	-3,7130
d	2	Tyrosine	7,2147	7,1804	-0,8498	-2,8409
d	1	Phenylalanine	7,3525	7,2973	0,9869	-0,6742
d	1	Phenylalanine	7,4036	7,3753	0,0023	-1,2351
t	1	Phenylalanine	7,4547	7,4125	-2,1705	-3,6061
-	-	Unknown	7,5018	7,4604	-1,1594	-2,9752
s	1	Xanthine	7,8583	7,8134	-1,1248	-3,3643
s	2	GTP	8,0456	8,0185	-0,9327	-2,2337
s	1	Inosine	8,2025	8,1871	0,9449	-0,2161
s	1	Inosine	8,2305	8,2049	0,4063	0,2593
s	1	AMP	8,2851	8,2598	5,2398	4,8649
s	1	Oxypurinol	8,3917	8,3745	-0,6719	-1,5183
s	1	Formate	8,4729	8,4426	-0,6432	-1,8520
s	1	AMP	8,6282	8,5897	3,7123	2,4320
-	-	Noise	9,5201	9,5	-0,8048	-1,1917

Type	H's	Metabolite	ppm 1	ppm 2	Spectrum 15	Spectrum 16
s		TSP	0,0428	-0,0466	448,1238	440,3327
		Noise	0,5401	0,52	-0,4041	0,8801
t	3	2-hydroxybutyrate	0,9229	0,8599	36,0853	20,9305
-	-	Unknown 1	0,9354	0,9247	5,1213	4,0252
t	3	Isoleucine	0,9479	0,9359	6,5286	4,6967
t	6	Leucine	0,9799	0,9503	12,1201	9,4968
d	3	Valine	1,0016	0,9799	5,2388	3,5250
d	3	Isoleucine	1,0248	1,0016	3,0329	2,5221
d	3	Valine	1,058	1,0337	3,2508	3,0784
-	-	Unknown 2	1,1007	1,0649	3,0262	1,8468
t	3	Ethanol	1,2478	1,1354	45,0771	24,0543
s	6	3-Hidroxyisovalerate	1,2576	1,2508	4,2186	2,4444
-	-	Unknown 3	1,3109	1,2737	44,0705	24,6796
d	3	Lactate	1,3503	1,3136	56,9863	35,6066
d	3	Alanine	1,5001	1,4645	13,8327	10,8815
m	2	2-hydroxybutyrate	1,6294	1,5908	5,8198	4,7054
m	2	2-hydroxybutyrate	1,6653	1,6299	7,3522	6,4574
m	2	Lysine	1,7974	1,6768	24,5609	24,2304
-	-	Unknown 4	1,8234	1,8096	1,9215	1,0197
-	-	Unknown 5	1,8582	1,8434	1,3603	1,8021
m	2	Lysine	1,9177	1,8747	6,2451	6,5219
s	3	Acetate	1,9285	1,9179	10,8122	10,7683
m	2	Glutamate	2,098	1,996	28,5506	32,0718
m	2	Glutamine	2,1356	2,1114	4,9670	6,0354
s	3	Hidroxyacetone	2,1498	2,1359	4,4665	4,7186
m	5	Methionine	2,1929	2,1506	9,8341	12,3954
s	-	Unknown 6	2,2326	2,2234	0,7574	0,8127
s	-	Unknown 7	2,2398	2,233	0,9440	0,5179
s	3	Acetoacetate	2,2706	2,258	3,6046	4,4064
m	1	Valine	2,2989	2,271	7,5808	6,5493
s	3	Methylacetoacetate	2,34	2,3302	1,0032	1,2967
-	-	Unknown 8	2,3522	2,3404	2,4683	3,2663
m	2	Glutamate	2,3651	2,3523	4,4898	4,8215
s	4	Succinate	2,3723	2,3652	1,6625	1,6419
s	3	Pyruvate	2,3801	2,3724	2,4975	2,9999
-	-	Unknown 9	2,4117	2,4035	0,7927	1,3146
-	-	Unknown 10	2,4674	2,4335	0,9036	0,9890
m	2	Glutamine	2,4983	2,4674	-0,1990	0,6050
m	2	GSH+GSSG	2,5612	2,5522	2,7167	2,8448
d	2	Citrate	2,5952	2,5224	7,2419	10,4007
d	2	Citrate	2,6434	2,6285	0,2856	1,6331
t	2	Methionine	2,663	2,6435	3,8699	4,8520
-	-	Unknown 11	2,673	2,6631	1,9805	2,6357
m	1	Aspartate	2,7038	2,6726	4,1629	6,6290

s	3	Sarcosine	2,7196	2,7089	-0,3872	0,0130
-	-	Unknown 12	2,7377	2,7293	1,2534	2,3381
m	1	Aspartate	2,8541	2,7938	3,2023	7,9845
s	9	Trimethylamine	2,8792	2,8591	-0,5809	3,0033
dd	2	GSH+GSSG	2,9859	2,9316	3,0271	7,4318
-	-	Unknown 13	3,0261	3,0147	1,6665	4,4620
s	3	Creatine	3,0391	3,0263	0,6266	2,0504
s	3	Creatinine	3,0539	3,0392	0,8549	1,5573
s	2	Malonate	3,1376	3,1285	-0,1640	0,8382
m	1	Phenylalanine	3,1671	3,1378	-0,9829	1,9005
s	9	Choline	3,2082	3,1984	4,8726	6,0060
s	9	O-Phosphocoline	3,2143	3,2082	0,3130	1,4667
s	9	Trimethylamine N-oxide	3,2245	3,2146	16,8747	18,4759
s	9	Carnitine	3,2343	3,2247	16,7351	17,7339
s	9	Betaine	3,2416	3,2342	6,6380	6,7474
t	2	Taurine	3,2803	3,2419	56,5615	60,5291
m	1	Phenylalanine	3,3122	3,2904	1,2328	3,7176
s	3	Methanol	3,377	3,3531	37,3578	15,8163
t	2	Taurine	3,4458	3,4102	48,6007	50,3871
-	-	Unknown 14	3,5415	3,5165	1,0803	4,6134
m	4	Glycerol	3,5685	3,5438	12,8101	13,8423
s	2	Glycine	3,5908	3,5687	9,1998	9,0171
d	1	Treonine	3,5976	3,5909	1,0953	1,5544
d	1	Valine	3,6236	3,6086	1,3731	3,3309
-	-	Unknown 15	3,6347	3,6244	1,9084	2,3883
m	4	Glycerol	3,664	3,6399	13,6380	11,7533
q	2	Ethanol	3,6888	3,664	13,2180	12,2475
-	-	Unknown 16	3,7264	3,7027	3,6373	1,7136
-	-	Unknown 17	3,7405	3,7346	1,4850	1,7473
m	1	Glutamate	3,7671	3,7415	9,5474	12,3032
dd + q	1	Serine + Alanine	3,818	3,7665	31,7652	34,4133
dd	1	Methionine	3,8593	3,8349	7,1732	8,0542
s	2	Betaine	3,8892	3,8839	0,8668	2,2653
-	-	Unknown 18	3,9138	3,8929	9,2410	11,6209
s	2	Creatine	3,9218	3,9141	3,0496	3,2101
dd	1	Tyrosine	3,9759	3,9389	6,3643	12,5504
m	2	Serine	4,0051	3,9761	22,8215	28,1422
s	2	Creatinine	4,0133	4,0054	1,9717	3,4886
m	2	Choline	4,0423	4,0356	0,7418	1,5467
-	-	Unknown 19	4,052	4,0427	0,0961	1,9787
-	-	Unknown 20	4,0608	4,0528	0,1201	1,6872
-	-	Unknown 21	4,0881	4,0611	0,7496	6,0993
-	-	Unknown 22	4,0972	4,0906	-0,1869	1,1374
q	1	Lactate	4,118	4,0972	5,0417	7,5201
q	1	Lactate	4,1289	4,1184	3,7067	4,3065

q	1	Lactate	4,1407	4,1291	1,1640	3,6659
-	-	Unknown 23	4,1519	4,1423	-0,1157	2,2216
m	1	Treonine	4,2436	4,1836	3,2720	14,3247
-	-	Unknown 24	4,2759	4,2442	0,7336	8,3916
-	-	Unknown 25	5,6375	5,6064	3,6313	-5,6111
d	1	Inosine	6,1597	6,1248	3,2541	-1,3890
		Noise	6,4595	6,4394	-0,4121	-2,3276
d	2	Tyrosine	6,9311	6,8779	-0,8939	-4,5652
dd	1	Xanthurenate	7,1227	7,0595	-0,0200	-5,3005
d	2	Tyrosine	7,2147	7,1804	0,3118	-2,7774
d	1	Phenylalanine	7,3525	7,2973	3,7032	-1,6777
d	1	Phenylalanine	7,4036	7,3753	1,8519	-2,4577
t	1	Phenylalanine	7,4547	7,4125	0,2267	-4,1929
-	-	Unknown	7,5018	7,4604	0,0815	-3,3722
s	1	Xanthine	7,8583	7,8134	-0,9190	-3,3071
s	2	GTP	8,0456	8,0185	-0,6420	-2,1632
s	1	Inosine	8,2025	8,1871	1,5764	0,3911
s	1	Inosine	8,2305	8,2049	1,2934	-0,3877
s	1	AMP	8,2851	8,2598	4,7003	3,3671
s	1	Oxypurinol	8,3917	8,3745	-0,2706	-1,6104
s	1	Formate	8,4729	8,4426	-0,5169	-1,2706
s	1	AMP	8,6282	8,5897	2,4053	1,6002
-	-	Noise	9,5201	9,5	-1,1603	-1,5623

Type	H's	Metabolite	ppm 1	ppm 2	Spectrum 17	Spectrum 18
s		TSP	0,0428	-0,0466	379,1548	387,5152
		Noise	0,5401	0,52	-0,3765	-0,0296
t	3	2-hydroxybutyrate	0,9229	0,8599	14,5719	14,7247
-	-	Unknown 1	0,9354	0,9247	2,9742	2,8091
t	3	Isoleucine	0,9479	0,9359	3,7952	3,6492
t	6	Leucine	0,9799	0,9503	7,9432	7,6382
d	3	Valine	1,0016	0,9799	3,1322	3,3073
d	3	Isoleucine	1,0248	1,0016	1,1497	1,9735
d	3	Valine	1,058	1,0337	2,3047	2,7351
-	-	Unknown 2	1,1007	1,0649	1,0043	0,7353
t	3	Ethanol	1,2478	1,1354	16,5008	24,1373
s	6	3-Hidroxyisovalerate	1,2576	1,2508	1,7886	2,1734
-	-	Unknown 3	1,3109	1,2737	14,1002	20,8984
d	3	Lactate	1,3503	1,3136	44,0803	54,2387
d	3	Alanine	1,5001	1,4645	16,9102	16,3040
m	2	2-hydroxybutyrate	1,6294	1,5908	2,4310	3,0556
m	2	2-hydroxybutyrate	1,6653	1,6299	4,1096	3,8548
m	2	Lysine	1,7974	1,6768	18,3369	21,5603
-	-	Unknown 4	1,8234	1,8096	1,4139	1,2492
-	-	Unknown 5	1,8582	1,8434	1,6454	1,4982
m	2	Lysine	1,9177	1,8747	4,6047	5,3937
s	3	Acetate	1,9285	1,9179	8,9245	9,3675
m	2	Glutamate	2,098	1,996	33,6086	31,2652
m	2	Glutamine	2,1356	2,1114	7,8968	7,3439
s	3	Hidroxyacetone	2,1498	2,1359	5,6456	5,0948
m	5	Methionine	2,1929	2,1506	16,5996	15,0136
s	-	Unknown 6	2,2326	2,2234	0,3915	0,1996
s	-	Unknown 7	2,2398	2,233	0,3944	1,0530
s	3	Acetoacetate	2,2706	2,258	6,2077	5,5445
m	1	Valine	2,2989	2,271	5,4889	6,2929
s	3	Methylacetoacetate	2,34	2,3302	0,7335	1,0922
-	-	Unknown 8	2,3522	2,3404	4,6769	4,5986
m	2	Glutamate	2,3651	2,3523	7,4587	6,4744
s	4	Succinate	2,3723	2,3652	2,4158	2,4151
s	3	Pyruvate	2,3801	2,3724	4,3175	3,6806
-	-	Unknown 9	2,4117	2,4035	0,6634	1,0935
-	-	Unknown 10	2,4674	2,4335	0,2643	1,3596
m	2	Glutamine	2,4983	2,4674	-1,3929	-0,8879
m	2	GSH+GSSG	2,5612	2,5522	4,9327	3,6854
d	2	Citrate	2,5952	2,5224	14,6379	13,8499
d	2	Citrate	2,6434	2,6285	0,3442	0,2745
t	2	Methionine	2,663	2,6435	5,0779	5,0584
-	-	Unknown 11	2,673	2,6631	3,1653	2,3822
m	1	Aspartate	2,7038	2,6726	6,1546	5,4982

s	3	Sarcosine	2,7196	2,7089	-0,2368	-0,0979
-	-	Unknown 12	2,7377	2,7293	1,0649	0,8885
m	1	Aspartate	2,8541	2,7938	5,7761	6,9192
s	9	Trimethylamine	2,8792	2,8591	-0,4336	0,8059
dd	2	GSH+GSSG	2,9859	2,9316	7,6863	5,0872
-	-	Unknown 13	3,0261	3,0147	2,8764	3,0256
s	3	Creatine	3,0391	3,0263	1,2237	1,1994
s	3	Creatinine	3,0539	3,0392	1,0993	1,1411
s	2	Malonate	3,1376	3,1285	0,7296	0,6577
m	1	Phenylalanine	3,1671	3,1378	19,5242	15,9896
s	9	Choline	3,2082	3,1984	14,7769	13,0062
s	9	O-Phosphocoline	3,2143	3,2082	0,5806	0,8418
s	9	Trimethylamine N-oxide	3,2245	3,2146	7,6778	7,7091
s	9	Carnitine	3,2343	3,2247	16,9862	15,6978
s	9	Betaine	3,2416	3,2342	4,0189	4,2167
t	2	Taurine	3,2803	3,2419	95,4094	83,6601
m	1	Phenylalanine	3,3122	3,2904	4,0098	4,2672
s	3	Methanol	3,377	3,3531	10,2922	19,2714
t	2	Taurine	3,4458	3,4102	83,9394	72,8560
-	-	Unknown 14	3,5415	3,5165	2,9389	4,1087
m	4	Glycerol	3,5685	3,5438	15,4498	15,9129
s	2	Glycine	3,5908	3,5687	6,6763	8,2510
d	1	Treonine	3,5976	3,5909	0,6276	0,7612
d	1	Valine	3,6236	3,6086	0,8722	1,7545
-	-	Unknown 15	3,6347	3,6244	1,7571	1,0780
m	4	Glycerol	3,664	3,6399	8,5041	10,6780
q	2	Ethanol	3,6888	3,664	7,5215	10,9418
-	-	Unknown 16	3,7264	3,7027	-0,3673	2,2495
-	-	Unknown 17	3,7405	3,7346	1,0091	1,3070
m	1	Glutamate	3,7671	3,7415	13,5217	13,3309
dd + q	1	Serine + Alanine	3,818	3,7665	43,5270	41,8352
dd	1	Methionine	3,8593	3,8349	10,6569	11,0227
s	2	Betaine	3,8892	3,8839	1,4209	1,6713
-	-	Unknown 18	3,9138	3,8929	12,4437	11,6973
s	2	Creatine	3,9218	3,9141	2,8908	2,5560
dd	1	Tyrosine	3,9759	3,9389	4,7031	7,5991
m	2	Serine	4,0051	3,9761	13,9006	16,8259
s	2	Creatinine	4,0133	4,0054	1,6501	2,2815
m	2	Choline	4,0423	4,0356	1,0390	1,3861
-	-	Unknown 19	4,052	4,0427	0,2536	0,4343
-	-	Unknown 20	4,0608	4,0528	-0,1761	0,5605
-	-	Unknown 21	4,0881	4,0611	2,8511	4,8837
-	-	Unknown 22	4,0972	4,0906	-0,3904	0,1648
q	1	Lactate	4,118	4,0972	4,9116	8,3583
q	1	Lactate	4,1289	4,1184	4,6344	6,1998

q	1	Lactate	4,1407	4,1291	2,6175	3,6316
-	-	Unknown 23	4,1519	4,1423	0,1193	0,5354
m	1	Treonine	4,2436	4,1836	8,6818	15,0571
-	-	Unknown 24	4,2759	4,2442	6,5844	8,8298
-	-	Unknown 25	5,6375	5,6064	1,0840	-1,9826
d	1	Inosine	6,1597	6,1248	4,8177	2,0284
		Noise	6,4595	6,4394	-0,6168	-1,2427
d	2	Tyrosine	6,9311	6,8779	-1,7815	-2,2404
dd	1	Xanthurenate	7,1227	7,0595	0,2369	-3,1220
d	2	Tyrosine	7,2147	7,1804	-1,0672	-0,5642
d	1	Phenylalanine	7,3525	7,2973	0,9590	-0,6315
d	1	Phenylalanine	7,4036	7,3753	-1,1940	-0,6317
t	1	Phenylalanine	7,4547	7,4125	-0,5770	-2,2913
-	-	Unknown	7,5018	7,4604	-1,3797	-1,4516
s	1	Xanthine	7,8583	7,8134	-1,6154	-2,6875
s	2	GTP	8,0456	8,0185	-0,4198	-1,1614
s	1	Inosine	8,2025	8,1871	0,5807	0,0002
s	1	Inosine	8,2305	8,2049	0,6803	-0,0072
s	1	AMP	8,2851	8,2598	6,9881	5,8953
s	1	Oxypurinol	8,3917	8,3745	-0,3885	-0,9676
s	1	Formate	8,4729	8,4426	-0,6577	-0,8063
s	1	AMP	8,6282	8,5897	-0,7233	-1,3281
-	-	Noise	9,5201	9,5	-0,4495	-0,7822

Type	H's	Metabolite	ppm 1	ppm 2	Spectrum 19	Spectrum 20
s		TSP	0,0428	-0,0466	120,3423	93,8941
		Noise	0,5401	0,52	-0,2420	0,0010
t	3	2-hydroxybutyrate	0,9229	0,8599	60,0879	52,8354
-	-	Unknown 1	0,9354	0,9247	13,6088	12,5096
t	3	Isoleucine	0,9479	0,9359	18,2525	17,0981
t	6	Leucine	0,9799	0,9503	50,6850	54,9601
d	3	Valine	1,0016	0,9799	19,6736	22,2558
d	3	Isoleucine	1,0248	1,0016	9,7893	11,5144
d	3	Valine	1,058	1,0337	15,3456	18,2493
-	-	Unknown 2	1,1007	1,0649	1,7793	2,0000
t	3	Ethanol	1,2478	1,1354	39,3093	33,3495
s	6	3-Hidroxyisovalerate	1,2576	1,2508	1,7878	1,6346
-	-	Unknown 3	1,3109	1,2737	5,8064	5,7027
d	3	Lactate	1,3503	1,3136	38,7266	39,0657
d	3	Alanine	1,5001	1,4645	56,8512	63,3593
m	2	2-hydroxybutyrate	1,6294	1,5908	11,9800	10,8943
m	2	2-hydroxybutyrate	1,6653	1,6299	17,5256	16,8326
m	2	Lysine	1,7974	1,6768	110,7014	111,8678
-	-	Unknown 4	1,8234	1,8096	5,9916	5,7891
-	-	Unknown 5	1,8582	1,8434	7,1345	6,8405
m	2	Lysine	1,9177	1,8747	39,0427	41,4587
s	3	Acetate	1,9285	1,9179	13,0875	12,9038
m	2	Glutamate	2,098	1,996	67,9089	67,3858
m	2	Glutamine	2,1356	2,1114	12,0335	12,3565
s	3	Hidroxyacetone	2,1498	2,1359	8,1665	8,9957
m	5	Methionine	2,1929	2,1506	10,1752	10,6401
s	-	Unknown 6	2,2326	2,2234	1,8162	1,7020
s	-	Unknown 7	2,2398	2,233	1,7953	1,6368
s	3	Acetoacetate	2,2706	2,258	86,8491	79,2393
m	1	Valine	2,2989	2,271	17,9148	17,2268
s	3	Methylacetoacetate	2,34	2,3302	2,3367	2,5389
-	-	Unknown 8	2,3522	2,3404	5,1550	5,6850
m	2	Glutamate	2,3651	2,3523	7,4361	8,3602
s	4	Succinate	2,3723	2,3652	5,7717	6,0281
s	3	Pyruvate	2,3801	2,3724	4,7738	4,4738
-	-	Unknown 9	2,4117	2,4035	1,1604	1,1078
-	-	Unknown 10	2,4674	2,4335	4,7508	5,0469
m	2	Glutamine	2,4983	2,4674	0,9019	1,3426
m	2	GSH+GSSG	2,5612	2,5522	2,2146	2,1843
d	2	Citrate	2,5952	2,5224	8,9521	9,2539
d	2	Citrate	2,6434	2,6285	1,7352	1,6654
t	2	Methionine	2,663	2,6435	4,2678	4,2844
-	-	Unknown 11	2,673	2,6631	1,1960	1,4766
m	1	Aspartate	2,7038	2,6726	3,7578	4,0257

s	3	Sarcosine	2,7196	2,7089	1,1756	1,2645
-	-	Unknown 12	2,7377	2,7293	0,7764	0,6805
m	1	Aspartate	2,8541	2,7938	4,1835	4,6864
s	9	Trimethylamine	2,8792	2,8591	1,9658	2,5279
dd	2	GSH+GSSG	2,9859	2,9316	8,5420	8,6609
-	-	Unknown 13	3,0261	3,0147	9,0705	9,2813
s	3	Creatine	3,0391	3,0263	9,9025	10,9585
s	3	Creatinine	3,0539	3,0392	5,7242	6,8687
s	2	Malonate	3,1376	3,1285	2,2718	2,3656
m	1	Phenylalanine	3,1671	3,1378	4,4136	4,9112
s	9	Choline	3,2082	3,1984	2,2846	2,0547
s	9	O-Phosphocoline	3,2143	3,2082	0,9865	0,9471
s	9	Trimethylamine N-oxide	3,2245	3,2146	6,2349	6,3957
s	9	Carnitine	3,2343	3,2247	7,2401	7,6000
s	9	Betaine	3,2416	3,2342	4,1951	4,7347
t	2	Taurine	3,2803	3,2419	31,2536	34,3611
m	1	Phenylalanine	3,3122	3,2904	2,6011	3,2285
s	3	Methanol	3,377	3,3531	12,7385	3,4225
t	2	Taurine	3,4458	3,4102	14,8859	16,7515
-	-	Unknown 14	3,5415	3,5165	2,0549	1,8587
m	4	Glycerol	3,5685	3,5438	9,0189	8,4949
s	2	Glycine	3,5908	3,5687	3,8198	3,3995
d	1	Treonine	3,5976	3,5909	1,1580	1,2358
d	1	Valine	3,6236	3,6086	2,2774	2,8168
-	-	Unknown 15	3,6347	3,6244	1,3971	1,5784
m	4	Glycerol	3,664	3,6399	8,5359	7,8911
q	2	Ethanol	3,6888	3,664	11,8264	11,7703
-	-	Unknown 16	3,7264	3,7027	4,5866	3,8891
-	-	Unknown 17	3,7405	3,7346	2,0743	1,8825
m	1	Glutamate	3,7671	3,7415	12,4621	11,4457
dd + q	1	Serine + Alanine	3,818	3,7665	15,9817	12,8684
dd	1	Methionine	3,8593	3,8349	6,1595	8,6378
s	2	Betaine	3,8892	3,8839	1,5151	1,7401
-	-	Unknown 18	3,9138	3,8929	6,2912	7,0385
s	2	Creatine	3,9218	3,9141	1,9814	2,1296
dd	1	Tyrosine	3,9759	3,9389	8,7804	7,9158
m	2	Serine	4,0051	3,9761	8,1718	5,5653
s	2	Creatinine	4,0133	4,0054	0,4366	0,0815
m	2	Choline	4,0423	4,0356	-0,1112	-0,0762
-	-	Unknown 19	4,052	4,0427	-0,1879	-0,0898
-	-	Unknown 20	4,0608	4,0528	-0,2039	-0,0551
-	-	Unknown 21	4,0881	4,0611	-0,8443	-0,0042
-	-	Unknown 22	4,0972	4,0906	0,0536	0,1170
q	1	Lactate	4,118	4,0972	0,6967	1,0088
q	1	Lactate	4,1289	4,1184	0,7346	0,9883

q	1	Lactate	4,1407	4,1291	1,0561	1,5320
-	-	Unknown 23	4,1519	4,1423	0,7536	0,8863
m	1	Treonine	4,2436	4,1836	2,1608	0,9243
-	-	Unknown 24	4,2759	4,2442	-0,2719	-1,0057
-	-	Unknown 25	5,6375	5,6064	1,5886	0,7396
d	1	Inosine	6,1597	6,1248	-1,0409	-0,5088
		Noise	6,4595	6,4394	-0,5874	-0,4228
d	2	Tyrosine	6,9311	6,8779	3,3602	4,3478
dd	1	Xanthurenate	7,1227	7,0595	2,3174	2,5362
d	2	Tyrosine	7,2147	7,1804	5,2349	6,3499
d	1	Phenylalanine	7,3525	7,2973	12,9373	12,3787
d	1	Phenylalanine	7,4036	7,3753	2,0853	2,5907
t	1	Phenylalanine	7,4547	7,4125	2,7826	3,9283
-	-	Unknown	7,5018	7,4604	3,0093	2,7312
s	1	Xanthine	7,8583	7,8134	3,5234	3,6382
s	2	GTP	8,0456	8,0185	3,1671	3,0703
s	1	Inosine	8,2025	8,1871	3,3780	3,2343
s	1	Inosine	8,2305	8,2049	3,7811	3,4804
s	1	AMP	8,2851	8,2598	0,9555	0,9785
s	1	Oxypurinol	8,3917	8,3745	0,7886	1,0394
s	1	Formate	8,4729	8,4426	0,5714	0,3966
s	1	AMP	8,6282	8,5897	-0,5446	0,0495
-	-	Noise	9,5201	9,5	-0,2815	-0,2192

Type	H's	Metabolite	ppm 1	ppm 2	Spectrum 21	Spectrum 22
s		TSP	0,0428	-0,0466	123,7200	197,7768
		Noise	0,5401	0,52	-0,1949	0,3355
t	3	2-hydroxybutyrate	0,9229	0,8599	55,6409	11,7789
-	-	Unknown 1	0,9354	0,9247	12,9865	3,7400
t	3	Isoleucine	0,9479	0,9359	17,5936	6,2382
t	6	Leucine	0,9799	0,9503	52,0152	38,8653
d	3	Valine	1,0016	0,9799	20,5701	19,3647
d	3	Isoleucine	1,0248	1,0016	10,5736	14,2173
d	3	Valine	1,058	1,0337	16,1226	20,9351
-	-	Unknown 2	1,1007	1,0649	1,5738	71,9936
t	3	Ethanol	1,2478	1,1354	35,7589	11476,4915
s	6	3-Hidroxyisovalerate	1,2576	1,2508	1,6075	21,8122
-	-	Unknown 3	1,3109	1,2737	5,3793	83,5559
d	3	Lactate	1,3503	1,3136	38,5764	50,5789
d	3	Alanine	1,5001	1,4645	58,9220	48,0496
m	2	2-hydroxybutyrate	1,6294	1,5908	11,7743	10,8604
m	2	2-hydroxybutyrate	1,6653	1,6299	17,2371	13,1784
m	2	Lysine	1,7974	1,6768	110,2407	74,8758
-	-	Unknown 4	1,8234	1,8096	5,9776	3,1147
-	-	Unknown 5	1,8582	1,8434	7,1657	3,1614
m	2	Lysine	1,9177	1,8747	39,3069	23,5198
s	3	Acetate	1,9285	1,9179	12,7151	14,7567
m	2	Glutamate	2,098	1,996	66,4639	19,8925
m	2	Glutamine	2,1356	2,1114	11,7235	6,5117
s	3	Hidroxyacetone	2,1498	2,1359	8,2811	6,9286
m	5	Methionine	2,1929	2,1506	10,1795	8,8162
s	-	Unknown 6	2,2326	2,2234	1,6698	1,5725
s	-	Unknown 7	2,2398	2,233	1,6416	1,7389
s	3	Acetoacetate	2,2706	2,258	82,9950	89,7348
m	1	Valine	2,2989	2,271	17,1512	15,7463
s	3	Methylacetoacetate	2,34	2,3302	2,1452	1,0345
-	-	Unknown 8	2,3522	2,3404	5,1785	3,4167
m	2	Glutamate	2,3651	2,3523	7,4887	5,1666
s	4	Succinate	2,3723	2,3652	5,7075	4,4685
s	3	Pyruvate	2,3801	2,3724	4,7729	2,9420
-	-	Unknown 9	2,4117	2,4035	1,0513	0,4010
-	-	Unknown 10	2,4674	2,4335	4,3591	3,7344
m	2	Glutamine	2,4983	2,4674	0,8267	7,8831
m	2	GSH+GSSG	2,5612	2,5522	2,2149	1,3624
d	2	Citrate	2,5952	2,5224	8,5424	5,2851
d	2	Citrate	2,6434	2,6285	1,5121	0,6237
t	2	Methionine	2,663	2,6435	4,0078	3,2461
-	-	Unknown 11	2,673	2,6631	1,4567	0,9647
m	1	Aspartate	2,7038	2,6726	3,9229	2,2920

s	3	Sarcosine	2,7196	2,7089	1,0183	0,0375
-	-	Unknown 12	2,7377	2,7293	0,5226	0,0377
m	1	Aspartate	2,8541	2,7938	3,8964	2,6931
s	9	Trimethylamine	2,8792	2,8591	2,1487	1,3753
dd	2	GSH+GSSG	2,9859	2,9316	8,0935	2,8586
-	-	Unknown 13	3,0261	3,0147	9,2395	5,0812
s	3	Creatine	3,0391	3,0263	10,0870	7,4473
s	3	Creatinine	3,0539	3,0392	6,1457	5,4493
s	2	Malonate	3,1376	3,1285	2,4055	2,1118
m	1	Phenylalanine	3,1671	3,1378	4,3869	3,7206
s	9	Choline	3,2082	3,1984	2,2471	2,4027
s	9	O-Phosphocoline	3,2143	3,2082	0,9587	0,8532
s	9	Trimethylamine N-oxide	3,2245	3,2146	6,5623	6,9166
s	9	Carnitine	3,2343	3,2247	7,6349	8,4960
s	9	Betaine	3,2416	3,2342	4,5954	5,0302
t	2	Taurine	3,2803	3,2419	34,0235	48,5632
m	1	Phenylalanine	3,3122	3,2904	2,6431	4,5632
s	3	Methanol	3,377	3,3531	33,5485	5,7547
t	2	Taurine	3,4458	3,4102	16,2849	21,9269
-	-	Unknown 14	3,5415	3,5165	1,5841	14,8890
m	4	Glycerol	3,5685	3,5438	7,5259	48,0094
s	2	Glycine	3,5908	3,5687	2,8495	45,6525
d	1	Treonine	3,5976	3,5909	1,0847	3,0265
d	1	Valine	3,6236	3,6086	2,4109	3,1816
-	-	Unknown 15	3,6347	3,6244	1,5970	0,4235
m	4	Glycerol	3,664	3,6399	8,1575	1574,5118
q	2	Ethanol	3,6888	3,664	11,9983	1706,5488
-	-	Unknown 16	3,7264	3,7027	3,9067	103,6723
-	-	Unknown 17	3,7405	3,7346	1,8412	7,4306
m	1	Glutamate	3,7671	3,7415	10,3428	21,9564
dd + q	1	Serine + Alanine	3,818	3,7665	12,3125	50,9028
dd	1	Methionine	3,8593	3,8349	7,5957	2,6585
s	2	Betaine	3,8892	3,8839	1,7030	0,2270
-	-	Unknown 18	3,9138	3,8929	6,7362	2,7614
s	2	Creatine	3,9218	3,9141	1,9796	0,8431
dd	1	Tyrosine	3,9759	3,9389	7,2648	4,7615
m	2	Serine	4,0051	3,9761	5,2795	7,4456
s	2	Creatinine	4,0133	4,0054	0,0621	0,3130
m	2	Choline	4,0423	4,0356	-0,1976	-0,3163
-	-	Unknown 19	4,052	4,0427	-0,2137	-0,4476
-	-	Unknown 20	4,0608	4,0528	-0,1685	-0,2158
-	-	Unknown 21	4,0881	4,0611	-0,3715	-1,1039
-	-	Unknown 22	4,0972	4,0906	0,1148	-0,1954
q	1	Lactate	4,118	4,0972	1,1371	-0,1508
q	1	Lactate	4,1289	4,1184	0,9588	0,1390

q	1	Lactate	4,1407	4,1291	1,2769	0,3141
-	-	Unknown 23	4,1519	4,1423	0,7631	-0,1004
m	1	Treonine	4,2436	4,1836	0,5972	-0,4797
-	-	Unknown 24	4,2759	4,2442	-1,2401	-1,1374
-	-	Unknown 25	5,6375	5,6064	1,2782	0,9764
d	1	Inosine	6,1597	6,1248	-0,6668	0,1528
		Noise	6,4595	6,4394	-0,5215	0,0075
d	2	Tyrosine	6,9311	6,8779	3,8880	4,3812
dd	1	Xanthurenate	7,1227	7,0595	2,6680	1,7355
d	2	Tyrosine	7,2147	7,1804	5,9264	5,1439
d	1	Phenylalanine	7,3525	7,2973	12,2960	8,7068
d	1	Phenylalanine	7,4036	7,3753	2,0543	1,9969
t	1	Phenylalanine	7,4547	7,4125	3,1672	4,3396
-	-	Unknown	7,5018	7,4604	2,8342	3,4090
s	1	Xanthine	7,8583	7,8134	3,5847	2,3961
s	2	GTP	8,0456	8,0185	3,0187	1,3828
s	1	Inosine	8,2025	8,1871	3,4693	2,9238
s	1	Inosine	8,2305	8,2049	3,7547	2,9013
s	1	AMP	8,2851	8,2598	0,7191	1,8471
s	1	Oxypurinol	8,3917	8,3745	1,0014	0,4172
s	1	Formate	8,4729	8,4426	0,4972	0,3782
s	1	AMP	8,6282	8,5897	-0,0449	1,5825
-	-	Noise	9,5201	9,5	-0,2422	0,2699

Type	H's	Metabolite	ppm 1	ppm 2	Spectrum 23	Spectrum 24
s		TSP	0,0428	-0,0466	255,6522	232,2115
		Noise	0,5401	0,52	-0,0515	-0,5586
t	3	2-hydroxybutyrate	0,9229	0,8599	10,0769	6,6196
-	-	Unknown 1	0,9354	0,9247	3,6683	3,3402
t	3	Isoleucine	0,9479	0,9359	6,6493	5,6298
t	6	Leucine	0,9799	0,9503	44,0776	41,7347
d	3	Valine	1,0016	0,9799	19,0344	17,4456
d	3	Isoleucine	1,0248	1,0016	13,1220	10,9346
d	3	Valine	1,058	1,0337	20,9137	17,7623
-	-	Unknown 2	1,1007	1,0649	70,8992	64,5350
t	3	Ethanol	1,2478	1,1354	11757,7006	11485,2418
s	6	3-Hidroxyisovalerate	1,2576	1,2508	5,2734	6,2099
-	-	Unknown 3	1,3109	1,2737	66,6877	71,7146
d	3	Lactate	1,3503	1,3136	56,3161	57,4246
d	3	Alanine	1,5001	1,4645	56,8895	58,9760
m	2	2-hydroxybutyrate	1,6294	1,5908	8,4994	12,5582
m	2	2-hydroxybutyrate	1,6653	1,6299	11,8488	15,0615
m	2	Lysine	1,7974	1,6768	80,1228	87,3203
-	-	Unknown 4	1,8234	1,8096	3,5414	3,8867
-	-	Unknown 5	1,8582	1,8434	4,1329	4,8273
m	2	Lysine	1,9177	1,8747	26,1869	28,2678
s	3	Acetate	1,9285	1,9179	19,4295	16,3932
m	2	Glutamate	2,098	1,996	22,3349	26,0362
m	2	Glutamine	2,1356	2,1114	7,6590	8,5160
s	3	Hidroxyacetone	2,1498	2,1359	8,4188	8,8412
m	5	Methionine	2,1929	2,1506	9,3956	10,0475
s	-	Unknown 6	2,2326	2,2234	1,3036	1,5823
s	-	Unknown 7	2,2398	2,233	2,8753	3,0516
s	3	Acetoacetate	2,2706	2,258	115,0150	114,1685
m	1	Valine	2,2989	2,271	14,8427	13,3872
s	3	Methylacetoacetate	2,34	2,3302	1,0516	1,0085
-	-	Unknown 8	2,3522	2,3404	4,0194	4,4380
m	2	Glutamate	2,3651	2,3523	6,3601	6,5771
s	4	Succinate	2,3723	2,3652	4,8520	4,7863
s	3	Pyruvate	2,3801	2,3724	3,2617	3,4264
-	-	Unknown 9	2,4117	2,4035	0,4589	0,4225
-	-	Unknown 10	2,4674	2,4335	3,6902	4,7379
m	2	Glutamine	2,4983	2,4674	4,8259	8,1915
m	2	GSH+GSSG	2,5612	2,5522	1,6635	1,9136
d	2	Citrate	2,5952	2,5224	4,5172	6,9373
d	2	Citrate	2,6434	2,6285	0,5495	1,0200
t	2	Methionine	2,663	2,6435	3,7185	4,3993
-	-	Unknown 11	2,673	2,6631	1,3539	1,1876
m	1	Aspartate	2,7038	2,6726	2,0552	2,9756

s	3	Sarcosine	2,7196	2,7089	0,0228	0,2820
-	-	Unknown 12	2,7377	2,7293	0,0311	0,0624
m	1	Aspartate	2,8541	2,7938	2,6401	3,9554
s	9	Trimethylamine	2,8792	2,8591	1,0692	1,9837
dd	2	GSH+GSSG	2,9859	2,9316	2,6131	3,9761
-	-	Unknown 13	3,0261	3,0147	5,8430	6,5698
s	3	Creatine	3,0391	3,0263	8,5898	8,8424
s	3	Creatinine	3,0539	3,0392	5,9067	6,0484
s	2	Malonate	3,1376	3,1285	2,3119	2,4819
m	1	Phenylalanine	3,1671	3,1378	3,5817	4,6586
s	9	Choline	3,2082	3,1984	3,0365	3,1151
s	9	O-Phosphocoline	3,2143	3,2082	0,9707	1,1510
s	9	Trimethylamine N-oxide	3,2245	3,2146	8,0320	8,5068
s	9	Carnitine	3,2343	3,2247	10,7904	11,1505
s	9	Betaine	3,2416	3,2342	5,9158	6,3062
t	2	Taurine	3,2803	3,2419	53,6646	55,7892
m	1	Phenylalanine	3,3122	3,2904	3,6382	3,8935
s	3	Methanol	3,377	3,3531	5,9697	6,8961
t	2	Taurine	3,4458	3,4102	26,3960	26,9607
-	-	Unknown 14	3,5415	3,5165	14,8809	15,6004
m	4	Glycerol	3,5685	3,5438	46,1237	34,3855
s	2	Glycine	3,5908	3,5687	40,8531	22,5764
d	1	Treonine	3,5976	3,5909	2,0450	1,6516
d	1	Valine	3,6236	3,6086	0,8173	2,3852
-	-	Unknown 15	3,6347	3,6244	-1,3120	0,8370
m	4	Glycerol	3,664	3,6399	1746,3784	1770,6694
q	2	Ethanol	3,6888	3,664	1793,4109	1765,7391
-	-	Unknown 16	3,7264	3,7027	23,4438	15,9245
-	-	Unknown 17	3,7405	3,7346	1,7812	1,2975
m	1	Glutamate	3,7671	3,7415	11,5020	10,1572
dd + q	1	Serine + Alanine	3,818	3,7665	43,9391	32,0203
dd	1	Methionine	3,8593	3,8349	2,4473	2,8659
s	2	Betaine	3,8892	3,8839	0,3628	0,3283
-	-	Unknown 18	3,9138	3,8929	3,2352	2,9153
s	2	Creatine	3,9218	3,9141	0,8889	0,8084
dd	1	Tyrosine	3,9759	3,9389	6,1535	5,7879
m	2	Serine	4,0051	3,9761	9,3924	8,5149
s	2	Creatinine	4,0133	4,0054	0,3217	0,3215
m	2	Choline	4,0423	4,0356	-0,5087	-0,4646
-	-	Unknown 19	4,052	4,0427	-0,1802	-0,6618
-	-	Unknown 20	4,0608	4,0528	-0,4753	-0,3476
-	-	Unknown 21	4,0881	4,0611	-1,1420	-0,7527
-	-	Unknown 22	4,0972	4,0906	-0,0112	-0,2862
q	1	Lactate	4,118	4,0972	0,0758	0,3481
q	1	Lactate	4,1289	4,1184	0,3605	0,3716

q	1	Lactate	4,1407	4,1291	0,0662	0,1160
-	-	Unknown 23	4,1519	4,1423	-0,2337	0,0617
m	1	Treonine	4,2436	4,1836	-1,4341	-0,8897
-	-	Unknown 24	4,2759	4,2442	-1,9492	-1,3645
-	-	Unknown 25	5,6375	5,6064	0,4655	0,3662
d	1	Inosine	6,1597	6,1248	0,1333	0,6336
		Noise	6,4595	6,4394	-0,4752	-0,4572
d	2	Tyrosine	6,9311	6,8779	4,3224	3,8938
dd	1	Xanthurenate	7,1227	7,0595	1,3500	0,6294
d	2	Tyrosine	7,2147	7,1804	5,6237	5,6600
d	1	Phenylalanine	7,3525	7,2973	8,7498	8,0614
d	1	Phenylalanine	7,4036	7,3753	1,9003	1,6043
t	1	Phenylalanine	7,4547	7,4125	4,2265	3,6848
-	-	Unknown	7,5018	7,4604	2,5577	2,3672
s	1	Xanthine	7,8583	7,8134	1,5462	1,7961
s	2	GTP	8,0456	8,0185	1,3707	1,1711
s	1	Inosine	8,2025	8,1871	3,1408	3,0355
s	1	Inosine	8,2305	8,2049	3,3337	2,4635
s	1	AMP	8,2851	8,2598	1,8862	2,0586
s	1	Oxypurinol	8,3917	8,3745	0,0045	0,0174
s	1	Formate	8,4729	8,4426	0,2662	0,6006
s	1	AMP	8,6282	8,5897	2,4454	2,4185
-	-	Noise	9,5201	9,5	-0,2340	-0,0688

Type	H's	Metabolite	ppm 1	ppm 2	Spectrum 25	Spectrum 26
s		TSP	0,0428	-0,0466	379,6985	350,2510
		Noise	0,5401	0,52	-0,7410	-1,0425
t	3	2-hydroxybutyrate	0,9229	0,8599	19,5332	32,9497
-	-	Unknown 1	0,9354	0,9247	4,0129	7,1363
t	3	Isoleucine	0,9479	0,9359	5,8961	10,2276
t	6	Leucine	0,9799	0,9503	12,3592	26,8341
d	3	Valine	1,0016	0,9799	4,3062	8,9256
d	3	Isoleucine	1,0248	1,0016	1,5072	4,5576
d	3	Valine	1,058	1,0337	2,7491	5,8819
-	-	Unknown 2	1,1007	1,0649	-0,4654	-0,2469
t	3	Ethanol	1,2478	1,1354	52,2745	40,3317
s	6	3-Hidroxyisovalerate	1,2576	1,2508	2,0479	2,0681
-	-	Unknown 3	1,3109	1,2737	6,2646	8,3846
d	3	Lactate	1,3503	1,3136	49,5184	56,5673
d	3	Alanine	1,5001	1,4645	22,7680	28,8906
m	2	2-hydroxybutyrate	1,6294	1,5908	4,1697	7,7665
m	2	2-hydroxybutyrate	1,6653	1,6299	5,0716	9,4503
m	2	Lysine	1,7974	1,6768	29,3321	52,9601
-	-	Unknown 4	1,8234	1,8096	5,2482	4,4551
-	-	Unknown 5	1,8582	1,8434	6,3285	5,8329
m	2	Lysine	1,9177	1,8747	8,3416	15,3128
s	3	Acetate	1,9285	1,9179	12,3978	13,8315
m	2	Glutamate	2,098	1,996	38,9936	43,6197
m	2	Glutamine	2,1356	2,1114	6,5436	7,9908
s	3	Hidroxyacetone	2,1498	2,1359	4,5776	5,7677
m	5	Methionine	2,1929	2,1506	16,0585	12,5481
s	-	Unknown 6	2,2326	2,2234	1,0354	0,9981
s	-	Unknown 7	2,2398	2,233	1,7835	1,1681
s	3	Acetoacetate	2,2706	2,258	196,0152	135,3169
m	1	Valine	2,2989	2,271	16,8745	25,9099
s	3	Methylacetoacetate	2,34	2,3302	0,8991	0,8367
-	-	Unknown 8	2,3522	2,3404	4,8310	3,6283
m	2	Glutamate	2,3651	2,3523	8,0515	5,7868
s	4	Succinate	2,3723	2,3652	9,8242	6,8903
s	3	Pyruvate	2,3801	2,3724	4,3606	4,4987
-	-	Unknown 9	2,4117	2,4035	1,5444	1,1758
-	-	Unknown 10	2,4674	2,4335	0,9417	0,8047
m	2	Glutamine	2,4983	2,4674	0,0249	-0,7359
m	2	GSH+GSSG	2,5612	2,5522	5,7941	2,9521
d	2	Citrate	2,5952	2,5224	18,8562	10,1648
d	2	Citrate	2,6434	2,6285	0,5030	0,3808
t	2	Methionine	2,663	2,6435	5,0616	3,7737
-	-	Unknown 11	2,673	2,6631	2,1080	1,3569
m	1	Aspartate	2,7038	2,6726	4,2495	3,7195

s	3	Sarcosine	2,7196	2,7089	-0,0133	0,1289
-	-	Unknown 12	2,7377	2,7293	0,5757	-0,1027
m	1	Aspartate	2,8541	2,7938	3,0158	1,2909
s	9	Trimethylamine	2,8792	2,8591	-0,7897	-0,0537
dd	2	GSH+GSSG	2,9859	2,9316	9,6814	5,0580
-	-	Unknown 13	3,0261	3,0147	2,6169	4,5903
s	3	Creatine	3,0391	3,0263	1,6485	4,1816
s	3	Creatinine	3,0539	3,0392	2,1867	2,9053
s	2	Malonate	3,1376	3,1285	1,3302	0,8757
m	1	Phenylalanine	3,1671	3,1378	3,0975	2,0058
s	9	Choline	3,2082	3,1984	2,7619	2,6004
s	9	O-Phosphocoline	3,2143	3,2082	0,3780	0,9389
s	9	Trimethylamine N-oxide	3,2245	3,2146	13,5740	8,5341
s	9	Carnitine	3,2343	3,2247	14,1913	11,0956
s	9	Betaine	3,2416	3,2342	5,3091	5,3952
t	2	Taurine	3,2803	3,2419	68,9440	44,3943
m	1	Phenylalanine	3,3122	3,2904	4,0650	3,4544
s	3	Methanol	3,377	3,3531	16,3805	4,1207
t	2	Taurine	3,4458	3,4102	40,9527	26,8160
-	-	Unknown 14	3,5415	3,5165	4,0804	2,4936
m	4	Glycerol	3,5685	3,5438	9,4276	8,8015
s	2	Glycine	3,5908	3,5687	3,8694	5,3065
d	1	Treonine	3,5976	3,5909	1,1960	1,4438
d	1	Valine	3,6236	3,6086	3,1923	2,9595
-	-	Unknown 15	3,6347	3,6244	3,3700	2,1174
m	4	Glycerol	3,664	3,6399	19,9388	13,7102
q	2	Ethanol	3,6888	3,664	23,6797	17,5674
-	-	Unknown 16	3,7264	3,7027	8,1566	6,2759
-	-	Unknown 17	3,7405	3,7346	2,6386	1,8333
m	1	Glutamate	3,7671	3,7415	10,6761	8,4303
dd + q	1	Serine + Alanine	3,818	3,7665	14,1801	14,9953
dd	1	Methionine	3,8593	3,8349	13,6249	9,9573
s	2	Betaine	3,8892	3,8839	1,6989	1,5059
-	-	Unknown 18	3,9138	3,8929	10,5623	7,7126
s	2	Creatine	3,9218	3,9141	3,2593	2,4937
dd	1	Tyrosine	3,9759	3,9389	5,4355	6,1887
m	2	Serine	4,0051	3,9761	6,3476	7,2272
s	2	Creatinine	4,0133	4,0054	-0,1438	0,1796
m	2	Choline	4,0423	4,0356	-0,4359	-0,3039
-	-	Unknown 19	4,052	4,0427	-0,0537	-0,6636
-	-	Unknown 20	4,0608	4,0528	0,1048	-0,3373
-	-	Unknown 21	4,0881	4,0611	0,0285	-1,0230
-	-	Unknown 22	4,0972	4,0906	0,0108	-0,4933
q	1	Lactate	4,118	4,0972	1,6138	0,5573
q	1	Lactate	4,1289	4,1184	2,0057	1,0385

q	1	Lactate	4,1407	4,1291	1,4492	0,7760
-	-	Unknown 23	4,1519	4,1423	1,0315	0,5448
m	1	Treonine	4,2436	4,1836	-0,4978	-2,6985
-	-	Unknown 24	4,2759	4,2442	-1,8870	-3,0262
-	-	Unknown 25	5,6375	5,6064	-0,6115	-0,1518
d	1	Inosine	6,1597	6,1248	3,6126	1,2104
		Noise	6,4595	6,4394	-1,1044	-0,3583
d	2	Tyrosine	6,9311	6,8779	-0,3794	0,7404
dd	1	Xanthurenate	7,1227	7,0595	-0,5279	1,2754
d	2	Tyrosine	7,2147	7,1804	0,4805	1,5584
d	1	Phenylalanine	7,3525	7,2973	8,1405	10,2984
d	1	Phenylalanine	7,4036	7,3753	0,1232	1,4648
t	1	Phenylalanine	7,4547	7,4125	0,9344	2,2196
-	-	Unknown	7,5018	7,4604	4,6854	4,6406
s	1	Xanthine	7,8583	7,8134	1,5786	2,9298
s	2	GTP	8,0456	8,0185	0,1530	1,6610
s	1	Inosine	8,2025	8,1871	1,1723	2,4544
s	1	Inosine	8,2305	8,2049	2,0947	3,4132
s	1	AMP	8,2851	8,2598	6,2240	3,3678
s	1	Oxypurinol	8,3917	8,3745	0,1815	0,3412
s	1	Formate	8,4729	8,4426	1,6196	2,4665
s	1	AMP	8,6282	8,5897	3,1974	3,5508
-	-	Noise	9,5201	9,5	-0,2406	-0,1031

Type	H's	Metabolite	ppm 1	ppm 2	Spectrum 27
s		TSP	0,0428	-0,0466	235,9037
		Noise	0,5401	0,52	-0,7687
t	3	2-hydroxybutyrate	0,9229	0,8599	40,5403
-	-	Unknown 1	0,9354	0,9247	8,8544
t	3	Isoleucine	0,9479	0,9359	12,4226
t	6	Leucine	0,9799	0,9503	31,4843
d	3	Valine	1,0016	0,9799	11,6102
d	3	Isoleucine	1,0248	1,0016	6,1276
d	3	Valine	1,058	1,0337	6,5039
-	-	Unknown 2	1,1007	1,0649	0,5743
t	3	Ethanol	1,2478	1,1354	36,7740
s	6	3-Hidroxyisovalerate	1,2576	1,2508	1,9921
-	-	Unknown 3	1,3109	1,2737	8,7462
d	3	Lactate	1,3503	1,3136	50,9004
d	3	Alanine	1,5001	1,4645	31,9104
m	2	2-hydroxybutyrate	1,6294	1,5908	10,4422
m	2	2-hydroxybutyrate	1,6653	1,6299	11,9274
m	2	Lysine	1,7974	1,6768	66,7672
-	-	Unknown 4	1,8234	1,8096	5,5158
-	-	Unknown 5	1,8582	1,8434	7,3346
m	2	Lysine	1,9177	1,8747	18,4343
s	3	Acetate	1,9285	1,9179	12,2249
m	2	Glutamate	2,098	1,996	52,8573
m	2	Glutamine	2,1356	2,1114	9,8215
s	3	Hidroxyacetone	2,1498	2,1359	6,2092
m	5	Methionine	2,1929	2,1506	14,3370
s	-	Unknown 6	2,2326	2,2234	1,5178
s	-	Unknown 7	2,2398	2,233	1,4073
s	3	Acetoacetate	2,2706	2,258	130,3161
m	1	Valine	2,2989	2,271	29,9068
s	3	Methylacetoacetate	2,34	2,3302	2,2652
-	-	Unknown 8	2,3522	2,3404	4,9085
m	2	Glutamate	2,3651	2,3523	6,8897
s	4	Succinate	2,3723	2,3652	6,0777
s	3	Pyruvate	2,3801	2,3724	4,4956
-	-	Unknown 9	2,4117	2,4035	1,2781
-	-	Unknown 10	2,4674	2,4335	1,8609
m	2	Glutamine	2,4983	2,4674	-0,2533
m	2	GSH+GSSG	2,5612	2,5522	3,5554
d	2	Citrate	2,5952	2,5224	13,5500
d	2	Citrate	2,6434	2,6285	1,0851
t	2	Methionine	2,663	2,6435	3,9803
-	-	Unknown 11	2,673	2,6631	1,7891
m	1	Aspartate	2,7038	2,6726	4,9936

s	3	Sarcosine	2,7196	2,7089	0,6686
-	-	Unknown 12	2,7377	2,7293	0,3243
m	1	Aspartate	2,8541	2,7938	3,1749
s	9	Trimethylamine	2,8792	2,8591	0,4687
dd	2	GSH+GSSG	2,9859	2,9316	6,5030
-	-	Unknown 13	3,0261	3,0147	6,0872
s	3	Creatine	3,0391	3,0263	5,1078
s	3	Creatinine	3,0539	3,0392	3,8895
s	2	Malonate	3,1376	3,1285	1,4173
m	1	Phenylalanine	3,1671	3,1378	3,0440
s	9	Choline	3,2082	3,1984	3,0643
s	9	O-Phosphocoline	3,2143	3,2082	0,9211
s	9	Trimethylamine N-oxide	3,2245	3,2146	10,0418
s	9	Carnitine	3,2343	3,2247	11,6181
s	9	Betaine	3,2416	3,2342	5,6585
t	2	Taurine	3,2803	3,2419	51,0256
m	1	Phenylalanine	3,3122	3,2904	5,7047
s	3	Methanol	3,377	3,3531	3,3930
t	2	Taurine	3,4458	3,4102	30,7037
-	-	Unknown 14	3,5415	3,5165	3,3423
m	4	Glycerol	3,5685	3,5438	7,6398
s	2	Glycine	3,5908	3,5687	5,3722
d	1	Treonine	3,5976	3,5909	1,6107
d	1	Valine	3,6236	3,6086	4,3385
-	-	Unknown 15	3,6347	3,6244	3,1090
m	4	Glycerol	3,664	3,6399	14,5847
q	2	Ethanol	3,6888	3,664	17,9634
-	-	Unknown 16	3,7264	3,7027	7,1295
-	-	Unknown 17	3,7405	3,7346	1,6836
m	1	Glutamate	3,7671	3,7415	7,7349
dd + q	1	Serine + Alanine	3,818	3,7665	15,6881
dd	1	Methionine	3,8593	3,8349	13,8762
s	2	Betaine	3,8892	3,8839	2,4252
-	-	Unknown 18	3,9138	3,8929	10,4167
s	2	Creatine	3,9218	3,9141	3,0703
dd	1	Tyrosine	3,9759	3,9389	6,8889
m	2	Serine	4,0051	3,9761	6,0768
s	2	Creatinine	4,0133	4,0054	0,4435
m	2	Choline	4,0423	4,0356	0,2256
-	-	Unknown 19	4,052	4,0427	0,4132
-	-	Unknown 20	4,0608	4,0528	0,3750
-	-	Unknown 21	4,0881	4,0611	1,6477
-	-	Unknown 22	4,0972	4,0906	0,1587
q	1	Lactate	4,118	4,0972	2,4947
q	1	Lactate	4,1289	4,1184	2,0715

q	1	Lactate	4,1407	4,1291	1,6811
-	-	Unknown 23	4,1519	4,1423	0,9759
m	1	Treonine	4,2436	4,1836	0,5921
-	-	Unknown 24	4,2759	4,2442	-0,8667
-	-	Unknown 25	5,6375	5,6064	0,0075
d	1	Inosine	6,1597	6,1248	0,3893
		Noise	6,4595	6,4394	-0,9308
d	2	Tyrosine	6,9311	6,8779	0,3777
dd	1	Xanthurenate	7,1227	7,0595	0,6349
d	2	Tyrosine	7,2147	7,1804	1,0601
d	1	Phenylalanine	7,3525	7,2973	8,6940
d	1	Phenylalanine	7,4036	7,3753	1,4072
t	1	Phenylalanine	7,4547	7,4125	1,6067
-	-	Unknown	7,5018	7,4604	1,8081
s	1	Xanthine	7,8583	7,8134	2,6251
s	2	GTP	8,0456	8,0185	1,5643
s	1	Inosine	8,2025	8,1871	2,6984
s	1	Inosine	8,2305	8,2049	3,7525
s	1	AMP	8,2851	8,2598	3,1584
s	1	Oxypurinol	8,3917	8,3745	0,2716
s	1	Formate	8,4729	8,4426	2,0872
s	1	AMP	8,6282	8,5897	2,4352
-	-	Noise	9,5201	9,5	-0,2449
