# Supplementary figures and images for: Development of an optimized method for processing peripheral blood mononuclear cells for 1H-nuclear magnetic resonance-based metabolomic profiling
Source: PLoS One. 2021 Feb 25;16(2):e0247668. doi: 10.1371/journal.pone.0247668 (PMC7906414; doi:10.1371/journal.pone.0247668)

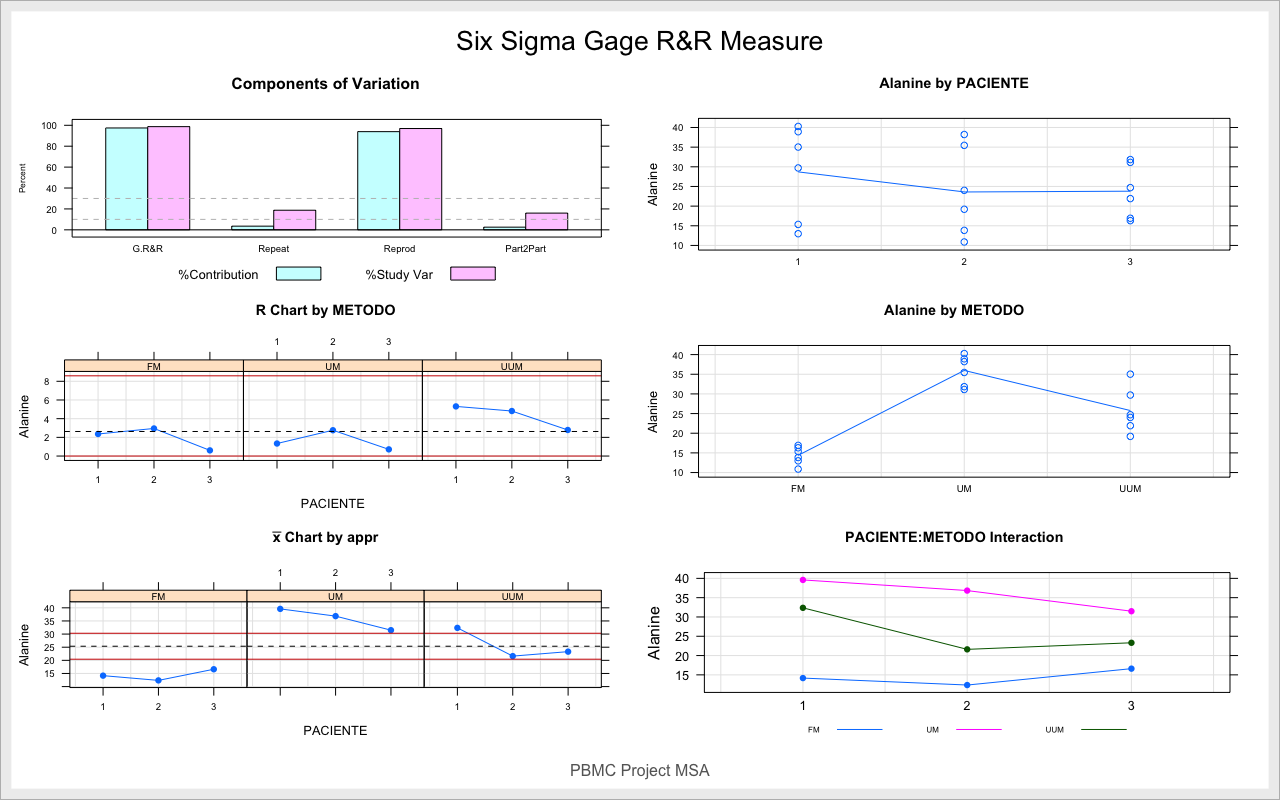

Supplement: S1 Fig — Comparative repeatability and reproducibility analysis between FM, UM and UUM, for the normalized concentration of Alanine. (TIF) [file pone.0247668.s003.tif]

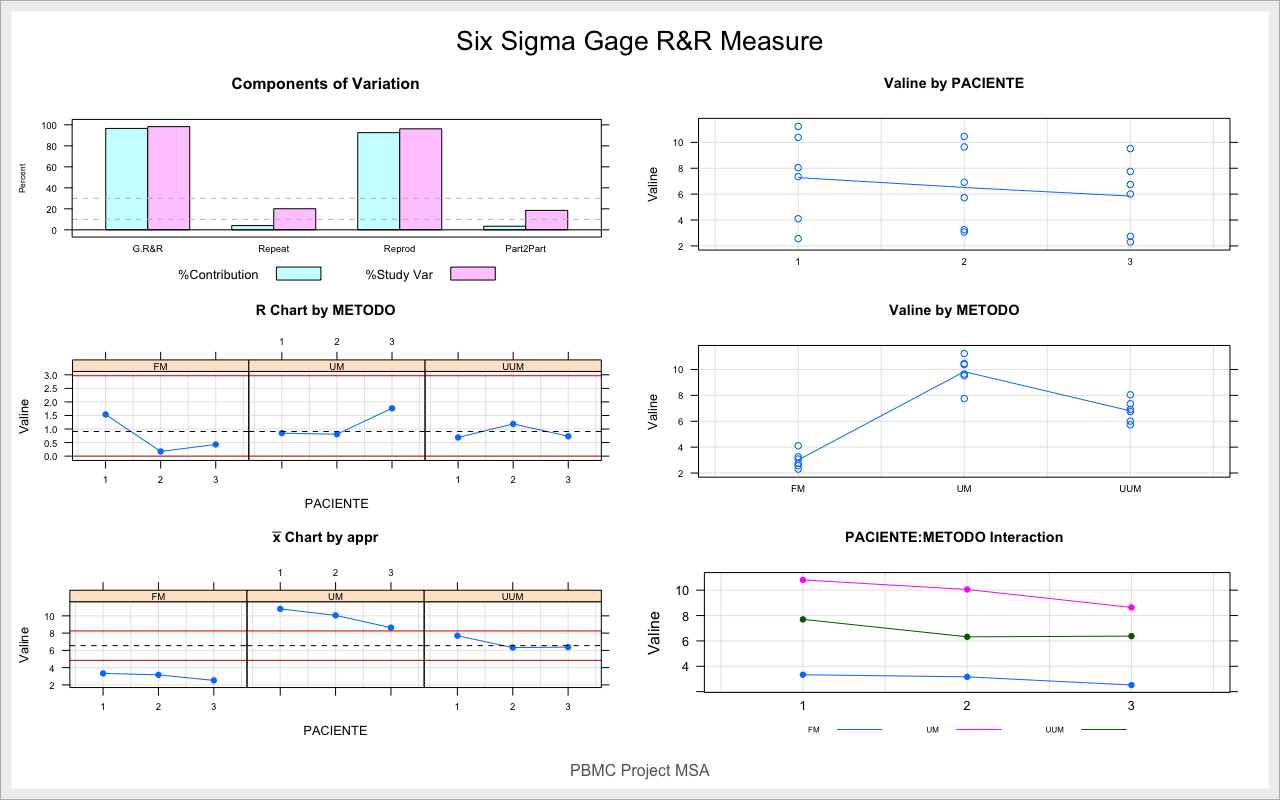

Supplement: S2 Fig — Comparative repeatability and reproducibility analysis between FM, UM and UUM, for the normalized concentration of Valine. (TIF) [file pone.0247668.s004.tif]

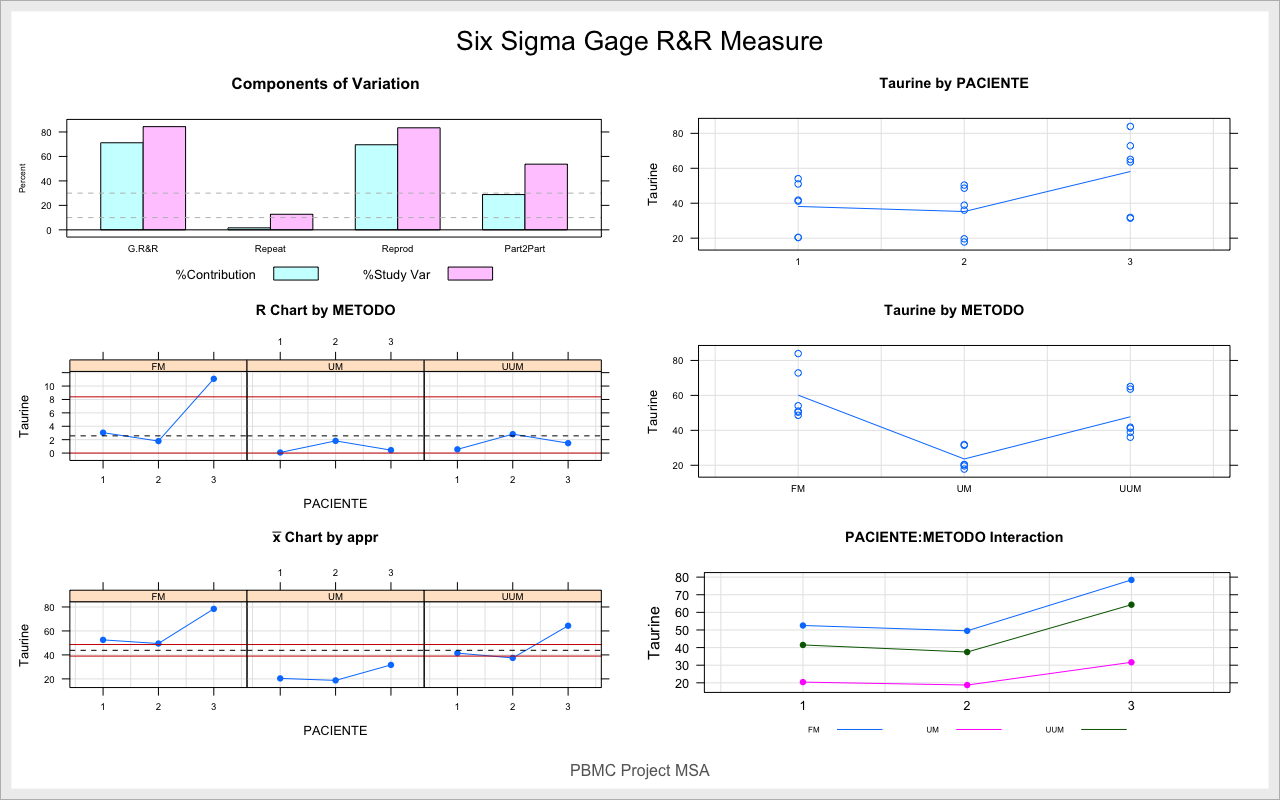

Supplement: S3 Fig — Comparative repeatability and reproducibility analysis between FM, UM and UUM, for the normalized concentration of Taurine. (TIF) [file pone.0247668.s005.tif]

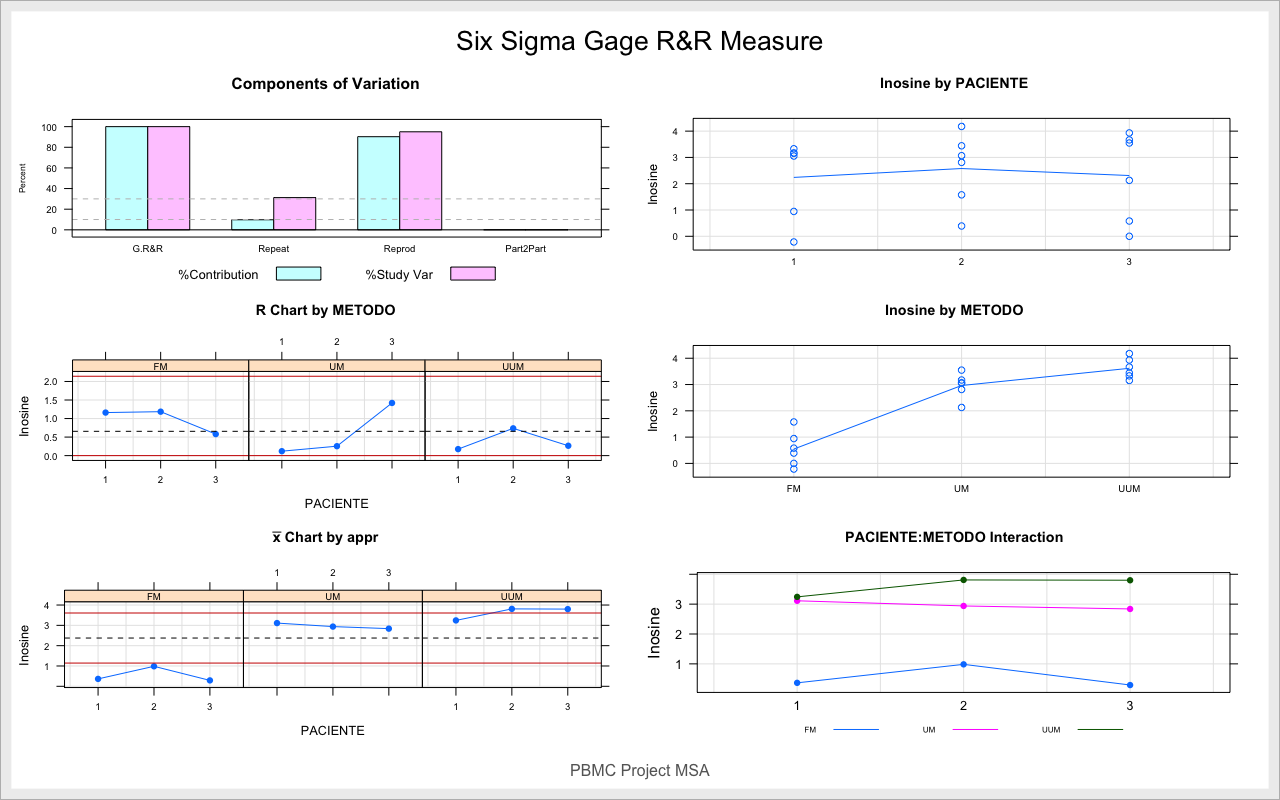

Supplement: S4 Fig — Comparative repeatability and reproducibility analysis between FM, UM and UUM, for the normalized concentration of Inosine. (TIF) [file pone.0247668.s006.tif]

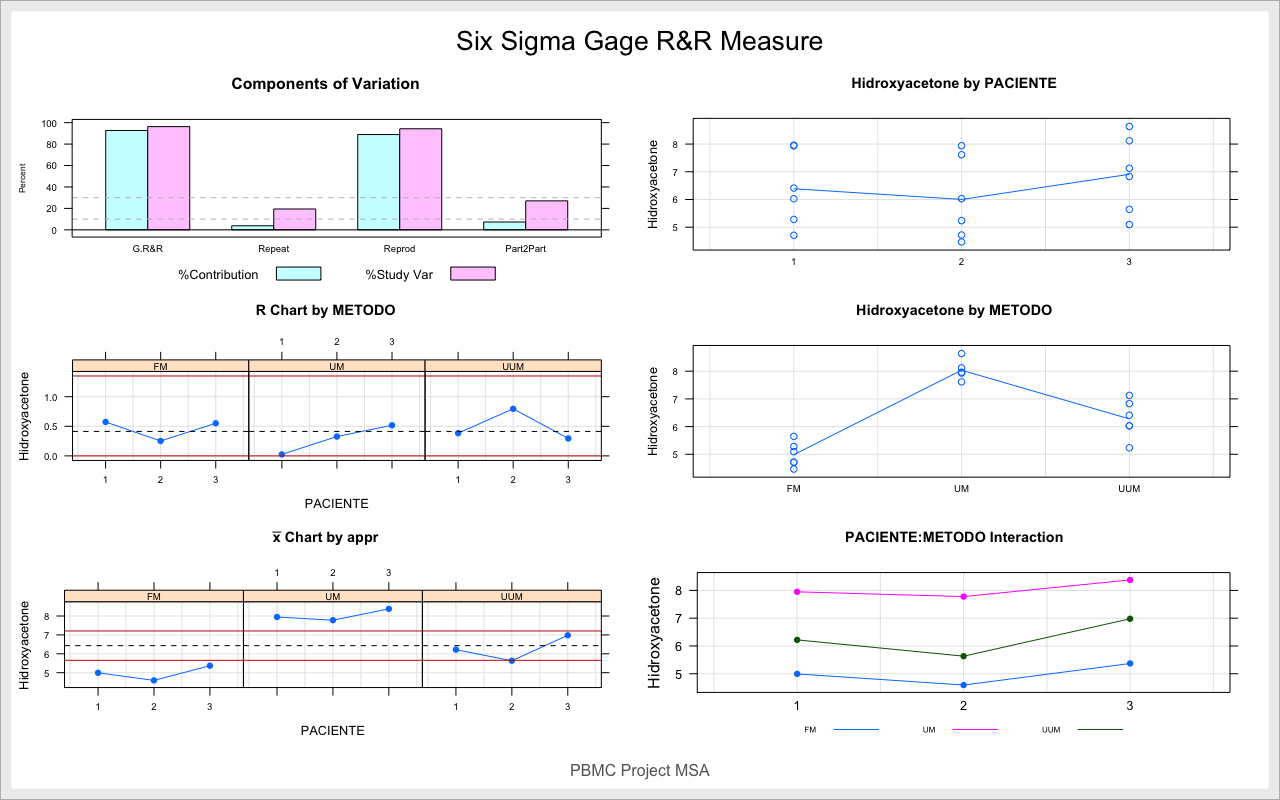

Supplement: S5 Fig — Comparative repeatability and reproducibility analysis between FM, UM and UUM, for the normalized concentration of Hidroxyacetone. (TIF) [file pone.0247668.s007.tif]

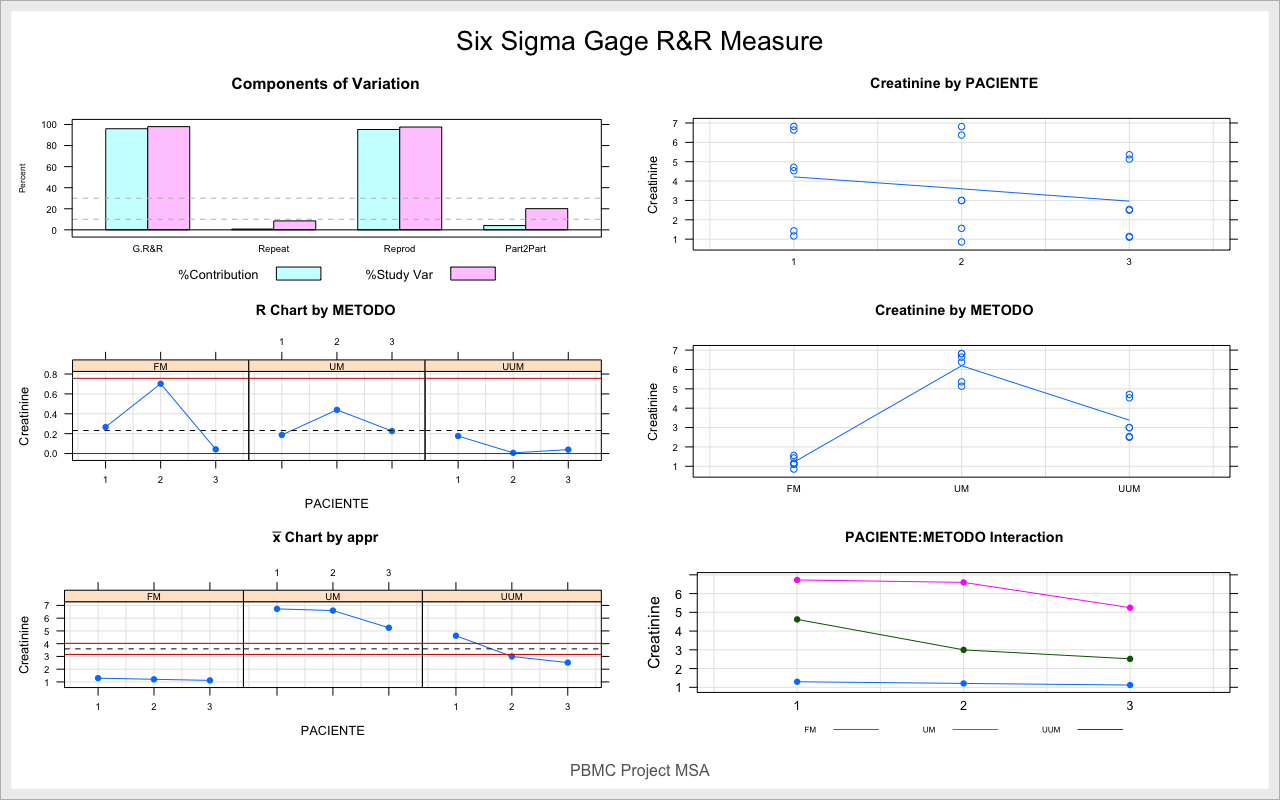

Supplement: S6 Fig — Comparative repeatability and reproducibility analysis between FM, UM and UUM, for the normalized concentration of Creatinine. (TIF) [file pone.0247668.s008.tif]

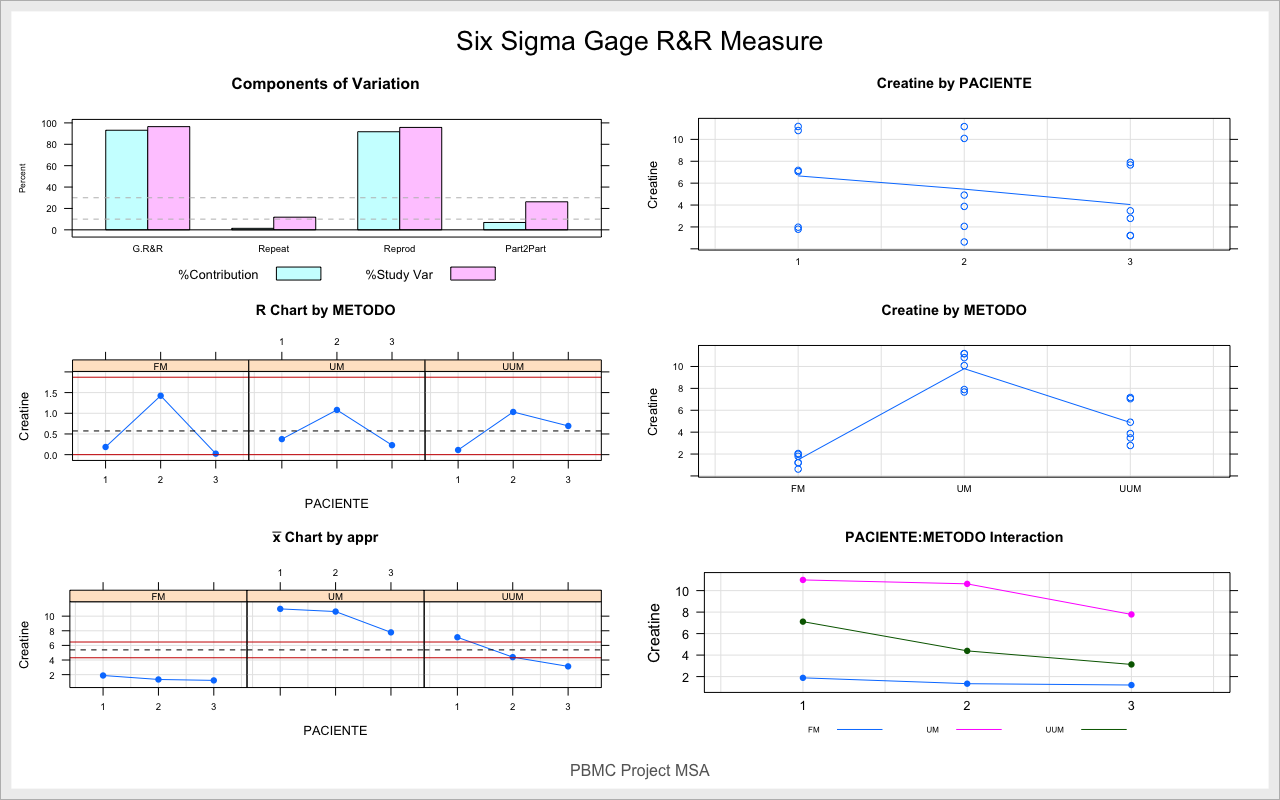

Supplement: S7 Fig — Comparative repeatability and reproducibility analysis between FM, UM and UUM, for the normalized concentration of Creatine. (TIF) [file pone.0247668.s009.tif]

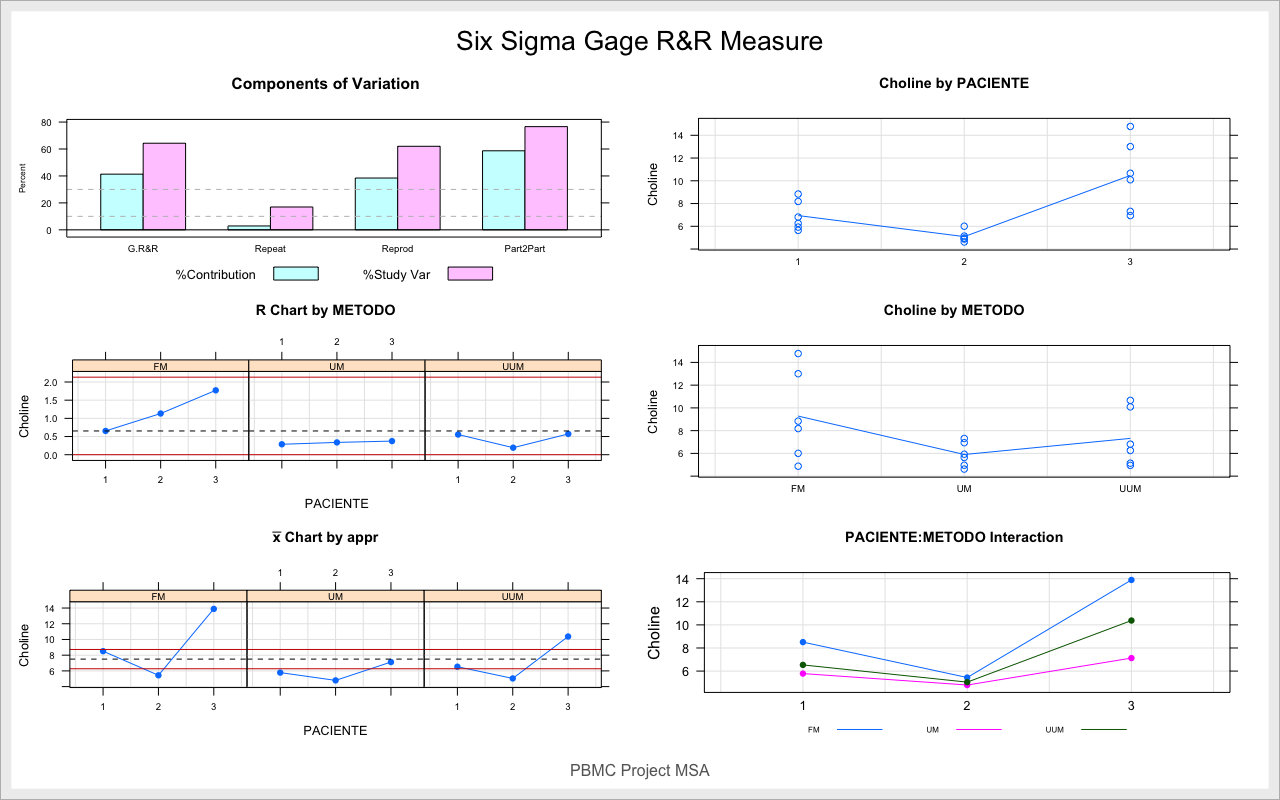

Supplement: S8 Fig — Comparative repeatability and reproducibility analysis between FM, UM and UUM, for the normalized concentration of Choline. (TIF) [file pone.0247668.s010.tif]
