# Supplementary material for: Development of an optimized method for processing peripheral blood mononuclear cells for 1H-nuclear magnetic resonance-based metabolomic profiling
Source: PLoS One. 2021 Feb 25;16(2):e0247668. doi: 10.1371/journal.pone.0247668 (PMC7906414; doi:10.1371/journal.pone.0247668)
Supplement: Attachment — Submitted filename: Response to Reviewers.pdf [file pone.0247668.s011.pdf]

Dear Dr. Oscar Millet
Academic Editor
PLOS ONE

Please, find enclosed our manuscript entitled *" Development of an optimized method for processing Peripheral Blood Mononuclear Cells for 1H-Nuclear Magnetic Resonance-based metabolomic profiling "* that we want to submit to *PlosOne*. The manuscript corresponds to reviewed version of the manuscript submitted previously with ID “[PONE-D-20-14510] - [EMID:615d9b8c13efb189]” and we include a point to point answer to the reviewer and the editor in this cover letter. Neither the manuscript nor any parts of its content are currently under consideration or published in another journal. All authors have seen and approved the new version of the manuscript and they declare no conflict of interest.

We feel we have fully addressed the concerns/comments raised by the reviewer and we thank her/him for her/his insights. Following her/his advice, we have included samples from different patients in our analysis and acquired independent replicate samples at two different days for each patients. We have also performed a more quantitative approach to compare the repeatability, reproducibility and sensitivity of the three methods, that confirms the improvement of our new method. Taken together, we think the manuscript is now more robust and consisting, and it would be of interest to the readers of *PlosOne*. We sincerely hope the manuscript is now ready to be accepted.

We thank you for your consideration and look forward to your decision.

Yours sincerely,

León Gabriel Gómez-Archila, Martina Palomino-Schätzlein (co-corresponding authors) on behalf of all the authors (Wildeman Zapata-Builes, Elkin Galeano)

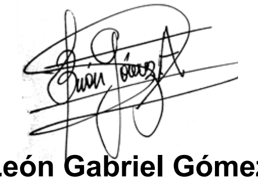

León Gabriel Gómez-Archila, Pharmaceutical Chemist
PhD student, Grupo de Investigación en Sustancias Bioactivas
Universidad de Antioquia
Calle 70 # 52-21
Medellín, Colombia.

Journal Requirements:

When submitting your revision, we need you to address these additional requirements.

1. Please ensure that your manuscript meets PLOS ONE's style requirements, including those for file naming. The PLOS ONE style templates can be found at

https://journals.plos.org/plosone/s/file?id=wjVg/PLOSOne_formatting_sample_main_body.pdf and

https://journals.plos.org/plosone/s/file?id=ba62/PLOSOne_formatting_sample_title_authors_affiliations.pdf

We have now revised the PLOS ONE's style requirement, to ensure that the new version of the manuscripts fulfils these requirements.

2. Please provide further details on participant numbers, demographics and recruitment. Please also provide the IRB approval number in the ethics statement."

We have now provided the required details in the methods section.

3. We note that this submission includes NMR spectroscopy data. We would recommend that you include the following information in your methods section or as Supporting Information files:

a) The make/source of the NMR instrument used in your study, as well as the magnetic field strength. For each individual experiment, please also list: the nucleus being measured; the sample concentration; the solvent in which the sample is dissolved and if solvent signal suppression was used; the reference standard and the temperature.

To our knowledge, all this information was already included in the methods section. We grateful if you could give us more detail if there is still any information missing.

b. A list of the chemical shifts for all compounds characterised by NMR spectroscopy, specifying, where relevant: the chemical shift (δ), the multiplicity and the coupling constants (in Hz), for the appropriate nuclei used for assignment.

We have now included the chemical shift information of each compound in Table 1. As we have only assigned already known compounds, the information about multiplicity and coupling constants can be easily found in databases (e. g. Human Metabolome Database https://hmdb.ca/), wherefore we think there is no need to include it in our work.

c. The full integrated NMR spectrum, clearly labelled with the compound name and chemical structure.
We also strongly encourage authors to provide primary NMR data files, in particular for new compounds which have not been characterised in the existing literature. Authors should provide the acquisition data, FID files and processing parameters for each experiment, clearly labelled with the compound name and identifier, as well as a structure file for each provided dataset. See our list of recommended repositories here: https://journals.plos.org/plosone/s/recommended-repositories

NMR spectra obtained with the three extraction methods are depicted in Figures 2 and 3. As these spectra are spectra from mixtures and not from pure compounds, it would be a bit confusing to include an integration of the signals in the spectra. However, we have now included a data table with the raw integration values in the supporting information.

4. We noticed you have some minor occurrence of overlapping text with previous publications, which needs to be addressed. In your revision ensure you cite all your sources (including your own works), and quote or rephrase any duplicated text outside the methods section. Further consideration is dependent on these concerns being addressed.

We have now rephrased the text in the first paragraph of the introduction and the lasts paragraphs of the discussion in order to avoid overlapping with previous publication. We have also added a new reference in the discussion section.

5.

We suggest you thoroughly copyedit your manuscript for language usage, spelling, and grammar. If you do not know anyone who can help you do this, you may wish to consider employing a professional scientific editing service.

The language usage in the new version of the manuscript has been revised by the professional service of Helen Warburton, member of the AEPD.

5. Review Comments to the Author

Please use the space provided to explain your answers to the questions above. You may also include additional comments for the author, including concerns about dual publication, research ethics, or publication ethics. (Please upload your review as an attachment if it exceeds 20,000 characters)

Reviewer #1: Gómez Archila et al. have evaluated two new methods for PBMCs metabolomic profiling by using NMR spectroscopy: Ultrasound Method (UM) and Ultrasound and Ultrafiltration Method (UUM). They have compared them against the gold standard Folch method in terms of sensitivity, processing time, spectrum quality, metabolite numbers and reproducibility.

The present study aims to apply a previously validated methodology based on high intensity ultrasounds sample processing to obtain PBMC extracted matrixes for NMR metabolomics profiling. The authors argue a higher degree of robustness and reproducibility by using these new methods, and reduced sample handling time compared to the traditional Folch extraction methods.

The sample size is not described, the conclusions are only qualitative, there's no statistical treatment of results, as there's not an analytical approach. The methods are interesting, although they should address considerably the study approach.
My major concern -considering the objective and nature of the study- is the absolutely lack of quantitative comparison.
This study should include the analysis of a sample set representing a wider range of biological conditions, to be able to report the analytical performance of the new approach compared against the gold standard. The authors do not report quantitative measurements

of robustness, reproducibility, fold changes, sensibility and variability. Neither replicates… as it appears to be 1 sample analysis.

We thank the reviewer for the evaluation. We have now completed our work with additional experiments and a more quantitative approach. In our initial study, we analysed three replicate samples from one patient for each method. In the study presented in the new manuscript, we have added an analysis of three more patients, acquire duplicates at two different measuring days. Furthermore, we now have done a quantitative statistical comparison of concentration values of all metabolites, and have calculated repeatability and reproducibility for selected, representative metabolites. We have also calculated the detection and quantification limits of the three methods. All this information has been included in the new results and discussion sections.

Minor: There are some aspects deserving a better approach:
In the abstract the authors should include methodological aspects.

We have now added more methodological details in the abstract.

Line 68- 70 Please, include references and clarify:
“For example in the case of, immunosuppressive diseases (HIV type), cancer and primary immunodeficiencies the availability of PBMCs will be very low.”

We have now rephrased the sentence and added new references.

Line 70 -74 To Consider Rewriting: I do not understand the construction.
“Therefore, taking into account cross-sectional or longitudinal studies, death, loss of contact, etc., it would be optimal to work with smaller blood and cell samples by changing the isolation method, the processing time (sample handling) delays and/or the cryopreservation technique that can affect the metabolic profile of the PBMCs obtained”.

We thank the reviewer for this comment, indeed the construction was a bit confusing. We have rephrased the sentence in the new version of the manuscript.

Methods: Please, include some details in the Human subjects description. It should include some complementary information. I do understand that healthy volunteers accepted to donate blood for research purposes, but how was created the validation cohort? There was some inclusion / exclusion criteria. % hematocrite, fasting, non-fasting, volume extraction, extraction tubes, etc…

We have now provided the required details about the volunteers in the methods section.

General comments: English review is mandatory

The language usage in the new version of the manuscript has been revised by the professional service of Helen Warburton, member of the AEPD.
