# Supplementary material for: Development of an optimized method for processing peripheral blood mononuclear cells for 1H-nuclear magnetic resonance-based metabolomic profiling
Source: PLoS One. 2021 Feb 25;16(2):e0247668. doi: 10.1371/journal.pone.0247668 (PMC7906414; doi:10.1371/journal.pone.0247668)
Supplement: Attachment — Submitted filename: Response to Reviewers.docx [file pone.0247668.s012.docx]

Reviewers' comments:

Reviewer's Responses to Questions

Comments to the Author

1. If the authors have adequately addressed your comments raised in a previous round of review and you feel that this manuscript is now acceptable for publication, you may indicate that here to bypass the “Comments to the Author” section, enter your conflict of interest statement in the “Confidential to Editor” section, and submit your "Accept" recommendation.

Reviewer #1: (No Response)

2. Is the manuscript technically sound, and do the data support the conclusions?

The manuscript must describe a technically sound piece of scientific research with data that supports the conclusions. Experiments must have been conducted rigorously, with appropriate controls, replication, and sample sizes. The conclusions must be drawn appropriately based on the data presented.

Reviewer #1: Yes

3. Has the statistical analysis been performed appropriately and rigorously?

Reviewer #1: I Don't Know

4. Have the authors made all data underlying the findings in their manuscript fully available?

The PLOS Data policy requires authors to make all data underlying the findings described in their manuscript fully available without restriction, with rare exception (please refer to the Data Availability Statement in the manuscript PDF file). The data should be provided as part of the manuscript or its supporting information, or deposited to a public repository. For example, in addition to summary statistics, the data points behind means, medians and variance measures should be available. If there are restrictions on publicly sharing data—e.g. participant privacy or use of data from a third party—those must be specified.

Reviewer #1: Yes

5. Is the manuscript presented in an intelligible fashion and written in standard English?

PLOS ONE does not copyedit accepted manuscripts, so the language in submitted articles must be clear, correct, and unambiguous. Any typographical or grammatical errors should be corrected at revision, so please note any specific errors here.

Reviewer #1: Yes

6. Review Comments to the Author

Please use the space provided to explain your answers to the questions above. You may also include additional comments for the author, including concerns about dual publication, research ethics, or publication ethics. (Please upload your review as an attachment if it exceeds 20,000 characters)

Reviewer #1: Gómez Archila et al. have evaluated two new methods for PBMCs metabolomic profiling by using NMR spectroscopy: Ultrasound Method (UM) and Ultrasound and Ultrafiltration Method (UUM). They have compared them against the gold standard Folch method in terms of sensitivity, processing time, spectrum quality, metabolite numbers and reproducibility.

The present study has addressed previous concerns and has really improved validating their methodology for NMR metabolomics profiling. Although some minor questions remained opened:

Line 83: Please, consider clarifying the highlighted term:

For example, with immunosuppressive diseases such as HIV [39], cancer [40] and primary immunodeficiencies, characterized by lower leukocyte production, the availability of PBMCs reduces.

We have now rephrased the sentence to make it clearer.

Line 199:

Please indicate the nuclear Overhauser Effect spectroscopy.

We have now indicated the term Nuclear Overhauser Effect Spectroscopy at the recommended place.

Line 218: Please, consider including into the discussion section the limitation associated with integrative methods to obtain metabolite concentration as the one presented in the manuscript; and the potential benefits of new UUM / UM methods on alternative mathematical treatment of the raw spectra based on methods (deconvolution processes). Would this approach differently improve quantification in any of the described experimental methods?

We have now included limitations and improvements of the new UUM/UM methods concerning the integration procedure in the third paragraph of the discussion.

Line 223: The statistical analysis should be carefully reviewed. There are different objectives, as it can be easily seen in the Results, but not in the Data analysis and statistics methods section. I would recommend to emphasize the different objectives (to evaluate the reproducibility, by using the variation coefficient, to evaluate the differences in the number between methods, and also in the area by difference on central tendency, ANOVA, etc.... It looks like it has been evaluated together. So, clarify how do you address the differences between central tendences? And how do you treat the duplicates/triplicates (not as independent / nor paired measurements).

We have now restructured the data analysis and statistics Materials and Methods section and added more details in order to clarify your questions about data analysis and statistics.

Line 224 Please, consider using the term mean in Table 2 instead of Average.

We have now changed the term “average” for “mean” in Table 2 according to your recommendation.

Line 227: Please review the use of central tendency instead of mean. Mean differences for t-tests, and median differences for Wilcoxon test.

We have now changed the term “measures of central tendency” for “means”, specifying “mean difference” for t-test and “median difference” for Wilcoxon test.

Statistics general comment: There's a problem with this particularly low sample size regarding the power of the test. The authors should state that in the discussion, groups are not large enough to have high power of detecting a meaningful difference say delta between the two means, but artifacts. You should add this limitation for the t test and the normality test.

We have now included the limitations mentioned by the referee in the first paragraph of the discussion.

Line 233: central difference instead of mean (as it depends on the test)

We have now changed the term “central (measures of central tendency)” instead of “mean”.

Line 254: From figure it is not easy to identify differences between Ultrasound and Ultrafiltration methods. The two methods are explained or only the UUM? please clarify as there’s a plural form in 256.

We have now changed the figure caption to make clear that only the UUM method is represented on the figure.

7. PLOS authors have the option to publish the peer review history of their article (what does this mean?). If published, this will include your full peer review and any attached files.

If you choose “no”, your identity will remain anonymous but your review may still be made public.

Do you want your identity to be public for this peer review? For information about this choice, including consent withdrawal, please see our Privacy Policy.

Reviewer #1: Yes: Dr. Nuria Amigo Grau
